# Biologic Phenotyping of the Human Small Airway Epithelial Response to Cigarette Smoking

**DOI:** 10.1371/journal.pone.0022798

**Published:** 2011-07-28

**Authors:** Ann E. Tilley, Timothy P. O'Connor, Neil R. Hackett, Yael Strulovici-Barel, Jacqueline Salit, Nancy Amoroso, Xi Kathy Zhou, Tina Raman, Larsson Omberg, Andrew Clark, Jason Mezey, Ronald G. Crystal

**Affiliations:** 1 Department of Genetic Medicine, Weill Cornell Medical College, New York, New York, United States of America; 2 Division of Pulmonary and Critical Care Medicine, Weill Cornell Medical College, New York, New York, United States of America; 3 Department of Public Health, Weill Cornell Medical College, New York, New York, United States of America; 4 Department of Molecular Biology and Genetics, Cornell University, Ithaca, New York, United States of America; 5 Department of Biological Statistics and Computational Biology, Cornell University, Ithaca, New York, United States of America; Comprehensive Pneumology Center, Germany

## Abstract

**Background:**

The first changes associated with smoking are in the small airway epithelium (SAE). Given that smoking alters SAE gene expression, but only a fraction of smokers develop chronic obstructive pulmonary disease (COPD), we hypothesized that assessment of SAE genome-wide gene expression would permit biologic phenotyping of the smoking response, and that a subset of healthy smokers would have a “COPD-like” SAE transcriptome.

**Methodology/Principal Findings:**

SAE (10th–12th generation) was obtained via bronchoscopy of healthy nonsmokers, healthy smokers and COPD smokers and microarray analysis was used to identify differentially expressed genes. Individual responsiveness to smoking was quantified with an index representing the % of smoking-responsive genes abnormally expressed (I_SAE_), with healthy smokers grouped into “high” and “low” responders based on the proportion of smoking-responsive genes up- or down-regulated in each smoker. Smokers demonstrated significant variability in SAE transcriptome with I_SAE_ ranging from 2.9 to 51.5%. While the SAE transcriptome of “low” responder healthy smokers differed from both “high” responders and smokers with COPD, the transcriptome of the “high” responder healthy smokers was indistinguishable from COPD smokers.

**Conclusion/Significance:**

The SAE transcriptome can be used to classify clinically healthy smokers into subgroups with lesser and greater responses to cigarette smoking, even though these subgroups are indistinguishable by clinical criteria. This identifies a group of smokers with a “COPD-like” SAE transcriptome.

## Introduction

Cigarette smoke, composed of >10^3^ xenobiotics and 10^14^ free radicals per puff, places a significant stress on the lung [1]–[4]. A particularly vulnerable cell population is the airway epithelium, the endoderm-derived, pseudostratified layer of cells lining the tracheobronchial tree [5], [6]. The airway epithelium is the first line of defense against cigarette smoke, and it is the epithelium of the small airways (<2 mm diameter, ≥6 generations) that shows the first morphologic changes in smokers [7]–[10]. With continued smoking, 15 to 20% of smokers progress and develop chronic obstructive pulmonary disease (COPD) [11]–[13]. The earliest smoking-induced abnormalities in the small airway epithelium include alterations of the cell cycle, repair and apoptosis, and a variety of changes resulting from oxidative stress [5], [7], [14]. In smokers who develop disease, there is epithelial dysfunction, leading to impaired mucociliary clearance, abnormalities in host defense, chronic colonization by pathogens, and mucus obstruction [14]–[17].

The airway epithelium of apparently healthy smokers demonstrates marked changes in gene expression compared to nonsmokers [18]–[22]. With the knowledge that, on average, smokers have an abnormal biologic phenotype of the small airway epithelium [21], but smoking causes disease in only a fraction of smokers, we asked two questions. First, is the gene expression profile of the small airway epithelium consistent among smokers, or do smokers exhibit variable gene expression profiles of the small airway epithelium, and if so, can this be used to categorize healthy smokers into biologic phenotypes? Second, to what extent, if any, do these biologic phenotypes of the small airway epithelium of healthy smokers overlap with those of the small airway epithelium of COPD smokers?

To assess these concepts, Affymetrix HG-U133 Plus 2.0 microarrays were used to evaluate small airway gene expression of healthy nonsmokers, healthy smokers and smokers with COPD. Comparison of the average small airway epithelium gene expression in healthy smokers *vs* nonsmokers identified 647 probe sets representing 375 unique known genes significantly differentially expressed in the healthy smokers. To quantify the variability observed among healthy smokers and allow biologic subcategorization of smokers, an index was calculated for overall small airway epithelium gene expression (I_SAE_) for each individual, representing the percentage of the 375 differentially expressed genes for which that individual's expression was abnormal (increased or suppressed), defined as more than 2 standard deviations from the mean in healthy nonsmokers. Not only did the I_SAE_ clearly discriminate smokers from nonsmokers, but it allowed subcategorization of healthy smokers based on the magnitude of the response to the stress of smoking, ranging from a “high” response with differential expression of hundreds of genes, to a “low” response with a gene expression profile close to that of nonsmokers. Interestingly, although high *vs* low responder healthy smokers have different small airway epithelial transcriptomes, and low responder healthy smokers have different small airway epithelial transcriptomes from COPD smokers, comparison of the gene expression profiles of the COPD smokers and high responder healthy smokers showed no differences. Together, these findings indicate that small airway epithelial gene expression can be used to phenotype clinically healthy smokers at a biologic level, a strategy that should be useful in helping to identify those smokers that may progress to develop airway disease.

Some of these results have been previously reported in the form of an abstract [23].

## Methods

### Study Population

All individuals were evaluated at the Weill Cornell NIH Clinical and Translational Science Center and Department of Genetic Medicine Clinical Research Facility, under clinical protocols approved by the Weill Cornell Medical College Institutional Review Board. All subjects provided written consent before any study procedures were undertaken. Healthy nonsmokers (n = 63; 47 in a primary set and 16 in a validation set) and healthy current cigarette smokers (n = 72; 58 in a primary set and 14 in a validation set) were recruited from the general population in New York City by posting advertisements in local newspapers and on electronic bulletin boards. Individuals with COPD (n = 36, 22 in a primary set and 14 in a validation set; all current smokers) were recruited in the same way and also from the outpatient clinics of the Division of Pulmonary and Critical Care Medicine.

Individuals were determined to be phenotypically normal or to have COPD based on standard history, physical exam, complete blood count, coagulation studies, liver function tests, urine studies, chest X-ray (and, where relevant, chest CT scan), EKG and pulmonary function tests. The GOLD criteria [13], based on post-bronchodilator FEV1/FVC ratio <70%, were used to define and stage COPD. Full inclusion/exclusion criteria are detailed in Text S1. This study is registered under the ClinicalTrials.gov identifiers NCT00224185 and NCT00224198.

### Sampling Airway Epithelium, RNA and Microarray Processing

Collection of small airway epithelial cells by fiberoptic bronchoscopy and extraction and processing of RNA for microarray analysis were carried out as previously described [21] (for full details, see Text S1).

### Web Deposition of Data

The raw data are all publicly available at the MIAME-compliant Gene Expression Omnibus (GEO) site (http://www.ncbi.nlm.nih.gov/geo/), a high-throughput gene expression/molecular abundance data repository curated by the National Center for Bioinformatics (NCBI) site, accession number GSE11784. Subsets of these samples have been used in unrelated analyses in other published manuscripts [21], [24]–[29]. The GEO accession numbers for the samples in the validation set (n = 16 nonsmokers, n = 14 healthy smokers and n = 14 COPD smokers) are shown in Table S1.

### Microarray Data Analysis - General

Microarray data were processed using the MAS5 algorithm (Affymetrix Microarray Suite Version 5 software), which takes into account the perfect match and mismatch probes. MAS5-processed data were normalized using GeneSpring by setting measurements <0.01 to 0.01 and by normalizing per chip to the median expression value on the array. Genes that were significantly modified by smoking were selected from the primary set of nonsmokers (n = 47) and healthy smokers (n = 58) according to the following criteria: (1) P call of “Present” in ≥20% of samples [19], [21], [22], [30]; (2) magnitude of fold change in average expression value for healthy smokers *vs* nonsmokers ≥1.5 [31]–[35]; and (3) p<0.01 using a t-test with a Benjamini-Hochberg correction to limit the false positive rate [36], except for the analysis of COPD smokers *vs* high and low responder healthy smokers in the validation set, in which due to lower sample size p<0.05 with a Benjamini-Hochberg correction was considered significant. Functional annotation was carried out using the NetAffx Analysis Center (www.affymetrix.com) to retrieve the Gene Ontology (GO) annotations from the National Center for Biotechnology (NCBI) databases. For genes without GO annotations, other public databases were searched (Human Protein Reference Database, Kyoto Encyclopedia of Genes and Genomes, PubMed).

This analysis generated a list of 647 probe sets representing 375 known genes that were significantly differentially expressed on average in healthy smokers *vs* healthy nonsmokers. This gene list was validated using unsupervised cluster analysis on these genes in the validation samples from nonsmokers (n = 16) and healthy smokers (n = 14). Additionally, the selection of genes was verified to be stable to normalization and specific technique by using singular value decomposition (SVD) [37] and prediction analysis of microarrays (PAM) [38] as additional methods to select genes that discriminated smokers from nonsmokers, to verify that the genes discriminating smokers from nonsmokers were robust with respect to method. Further details of these methods are in Text S1.

TaqMan PCR was used to confirm expression levels of selected smoking-responsive genes. For full details, see Text S1.

### Index of Airway Gene Expression

The gene expression index for small airway epithelium (I_SAE_) was calculated using the 375 smoking-responsive genes. For genes represented by more than one probe set, the probe set with the lowest p value was used. Expression values were log_2_ transformed. For each gene, a mean and standard deviation were calculated from the values in nonsmokers, and the normal range was defined as within 2 standard deviations of the mean, in the direction of the smoking-induced change (i.e., for smoking-suppressed genes, the threshold for normal equals the mean minus 2 standard deviations and for smoking-induced genes the threshold for normal equals the mean plus 2 standard deviations). The number of genes expressed outside the normal range was summed for each individual and divided by 375, the number of genes represented by the index. For the small airway epithelium, therefore,
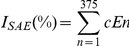
where E1 has a value of 1 if the expression level for gene 1 is >2 SD above or below that of healthy nonsmokers or has a value of 0 if the expression level is ≤2 SD above or below that of healthy nonsmokers; E2 is the index for gene 2, etc., and the constant (c = 100/375) normalizes the index to the percent of the 375 genes that are outside of the range of healthy nonsmokers.

To establish “high” and “low” responder phenotypes of healthy smokers, the smokers were divided into two groups based on the I_SAE_ values. The healthy smokers with index values less than or equal to the median were categorized as “low” responders and those above the median were labeled “high” responders to the stress of smoking. To assess the stability of the I_SAE_ over time, a subset of healthy nonsmokers (n = 7) and healthy smokers (n = 6) underwent bronchoscopy at a second time point 1 to 25 months from the 1^st^ bronchoscopy. Only data from the first bronchoscopy were used in the analysis of smoking-responsive genes and the development of the I_SAE_ metric. To evaluate stability over time, the I_SAE_ was calculated separately on data from each individual's 2^nd^ bronchoscopy. The I_SAE_ was further evaluated in terms of distribution among phenotypic groups, potential confounders, robustness and sampling properties, and robustness of the classification of high and low responder smokers; these analyses are described in Text S1.

### Subgroup Analyses

Having defined subgroups of low and high responder smokers based on the I_SAE_, the genome-wide gene expression of the subgroups of low and high responder healthy smokers was compared to each other and to the independent group of COPD smokers. Criteria for significantly changed genes were as described above. These analyses were carried out using the groups of high and low responders defined by the median I_SAE_, as described above, and were repeated using a different classification in which the highest 20% of I_SAE_ values were considered high responders and the remaining 20% considered low responders.

Principal components analysis was performed using R (The R Foundation for Statistical Computing, version 2.5.1) to compare small airway epithelial gene expression for high *vs* low responder healthy smokers using log2-transformed expression values for the differentially expressed genes. The data were visualized by plotting on a two-dimensional graph representing the first two principal components.

## Results

### Study Population and Biologic Samples

The primary study population of 127 individuals included 47 nonsmokers, 58 healthy smokers and 22 smokers with COPD (GOLD I, n = 9; GOLD II, n = 11; and GOLD III, n = 2; Table 1). The three groups were of similar distribution of gender (predominately male, p>0.3, Chi-square) and race (mostly of African or European descent, p>0.2, Chi-square). The COPD group was older (p<0.001) than the two other groups. The lung function of the normal nonsmokers and normal smokers was similar (p>0.07, all comparisons). On the average, smokers with COPD reported more pack-yr of smoking (p<0.05). Smokers with COPD had a reduced FEV1 (% predicted) and FEV1/FVC (% observed), consistent with the definition of COPD [13], and a reduced DLCO (% predicted) compared to the normal nonsmokers and smokers (p<0.0001, all comparisons). Among the 22 COPD smokers, 7 were on medications for COPD (2 of 9 GOLD I, 3 of 11 GOLD II, and 2 of 2 GOLD III). The classes of medications included short- and long-acting β-agonists, short- and long-acting anticholinergics, inhaled corticosteroids, systemic corticosteroids and theophylline; several of those treated were on multiple classes of medications. The total number of cells recovered by brushing was similar in all groups (p>0.07). The percent epithelial cells recovered was, on average, ≥98% in all groups. Smokers with and without COPD had fewer ciliated cells, more secretory cells, and more undifferentiated cells than nonsmokers (p<0.01 for all). Among all samples, the average RNA yield was 24.1±10.8 µg/subject. The validation population of nonsmokers, healthy smokers and COPD smokers was similar to the primary population in all of these respects (Table 1).

**Table 1:** Demographics of the Study Population and Biologic Samples^1^.

	Primary Set			Validation Set		
Parameter	Healthy nonsmokers	Healthy smokers	Smokers with COPD^2^	Healthy nonsmokers	Healthy smokers	Smokers with COPD
n	47	58	22	16	14	14
Sex (male/female)	33/14	38/20	18/4	7/9	14/0	10/4
Age (yr)	42.4±11.2	42.9±7.2	51.5±8.5	36.5±12.8	40.7±9.1	50.2±5.4
Race (B/W/O)^3^	23/18/6	35/14/9	8/9/5	4/6/6	9/2/3	5/4/5
Smoking history (pack-yr)	-	27.5±16.7	41.0±28.2	-	23.8±12.1	34.9±13.5
Urine nicotine (ng/ml)	-	1283±1580	1122±773	-	1596±1359	2132±1658
Urine cotinine (ng/ml)	-	1303±988	2410±1436	-	1568±1257	1732±743
Blood carboxyhemoglobin (%)	-	2±2	3±2	-	1±1	3±2
Pulmonary function parameters^4^						
FVC	107±13	109±13	97±21	107±10	112±11	115±17
FEV1	107±14	109±15	71±21	104±11	107±11	95±16
FEV1/FVC	82±6	81±5	61±8	82±5	78±4	66±4
TLC	100±14	100±12	101±22	94±11	102±14	111±14
DLCO	99±14	93±11	75±19	94±12	93±10	80±12
Gold stage (I/II/III)^2^	-	-	9/11/2	-	-	10/2/0
Medication use						
β-agonist	-	-	7	-	-	1
Anticholinergic	-	-	2	-	-	0
Inhaled corticosteroid	-	-	3	-	-	0
Epithelial cells^5^						
Number recovered ×10^6^	6.0±1.9	7.2±3.0	6.8±3.6	7.4±3.1	8.5±4.1	5.7±1.7
% epithelial cells	99.3±1.1	99.1±1.3	98.9±1.4	97.8±1.6	98.0±1.8	97.8±1.3
% inflammatory cells	0.7±1.1	0.9±1.3	1.1±1.4	2.2±1.6	1.9±1.8	2.1±1.4
Differential cell count^6^						
Ciliated (%)	74.3±7.4	65.6±12.6	63.5±10.9	65.0±9.2	57.0±6.8	56.1±8.4
Secretory (%)	6.6±3.5	9.1±4.6	11.9±5.6	6.9±4.0	5.8±2.8	10.7±5.5
Basal (%)	11.1±5.3	12.8±6.7	11.9±6.3	16.6±8.1	20.5±10.2	19.6±9.1
Undifferentiated columnar (%)	7.3±3.2	11.9±6.7	11.6±3.7	9.1±3.8	14.4±6.9	11.4±4.7

1 Data are presented as mean ± standard deviation.

2 Smokers with “established COPD” defined by the GOLD criteria (13); the COPD smoker group included: GOLD I n = 9, GOLD II n = 11, and GOLD III n = 2.

3 B = black, W = white, O = other.

4 Pulmonary function testing parameters are given as % of predicted value with the exception of FEV1/FVC, which is reported as % observed; FVC - forced vital capacity, FEV1 - forced expiratory volume in 1 sec, TLC - total lung capacity, DLCO - diffusing capacity. For individuals with COPD, FVC, FEV1, and FEV1/FVC are post-bronchodilator values.

5 Small airway epithelium.

6 As a % of small airway epithelium recovered.

### Effect of Smoking on Gene Expression in the Small Airway Epithelium

**Figure 1:**
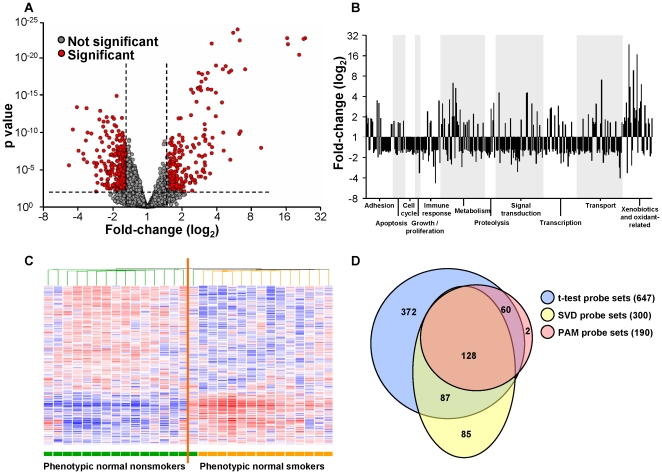
Differential gene expression profiles in the small airway epithelium in nonsmokers and healthy smokers. Expression levels normalized by array were compared for n = 58 healthy smokers and n = 47 healthy nonsmokers for all probe sets “present” in at least 20% of samples. **A.** Volcano plot. The mean expression level for healthy smokers *vs* healthy nonsmokers was assessed for fold-change (abscissa) *vs* p value (ordinate) by t-test. Each probe set is represented by a filled circle, with probe sets that are not significantly different in healthy smokers compared to healthy nonsmokers in gray and those that are significantly different in the 2 groups in red. Probe sets with a higher expression level in healthy smokers are to the top right and those with a lower expression level in healthy smokers are to the top left. There are 647 probe sets representing 375 known genes that are significantly up- and down-regulated by smoking. Only probe sets corresponding to known genes were used to construct the index for small airway epithelium gene expression (I_SAE_). When there is more than one probe set for a given gene, the decision as to which probe set is used for further analysis was made as described in Methods. **B.** Categories of genes differentially modified by smoking in the small airway epithelium. Shown is a skyscraper plot of fold-changes (log_2_ scale) for probe sets significantly differentially expressed in healthy smokers *vs* nonsmokers. Known genes significantly up-regulated in healthy smokers have fold-changes >1; those significantly down-regulated in healthy smokers have fold-changes <1. Alternating gray and white bands highlight the probe sets belonging to specific functional categories. **C.** Unsupervised cluster analysis. Probe sets expressed above average are represented in red, below average in blue, and average in white. Each row represents one of the 375 smoking-responsive genes used in the index, and each column represents an individual subject from the validation set of healthy nonsmokers and healthy smokers. Healthy smokers (n = 14) are indicated by orange, healthy nonsmokers (n = 16) by green. **D.** Venn diagram. Smoking-responsive probe sets identified by the main, t-test analysis (647 probe sets) are represented in blue, probe sets identified by singular value decomposition (SVD, 300 probe sets) in yellow, and probe sets identified by prediction analysis of microarrays (PAM, 190 probe sets) in pink. The intersection of the ovals represents the overlap between genes selected using each method, i.e., 87 genes are smoking-responsive in the t-test and SVD analyses (green), 60 probe sets overlap between the t-test and PAM analyses (purple), and 128 genes were significant in all 3 analyses (brown).

Comparing healthy smokers to healthy nonsmokers, 647 probe sets were identified as having expression levels significantly responsive to smoking (Figure 1A, Table S2). The identified probe sets were grouped into functional categories based on annotations in public databases and literature review. The specific categories containing the greatest numbers of changed genes were metabolism and transport. In contrast, the xenobiotic and oxidant-related category contained the genes displaying the greatest magnitude of change in expression levels in healthy smokers (Figure 1B).

Three methods were used to validate the smoking signature. First, unsupervised cluster analysis was carried out using this signature on the independent validation set of normal nonsmokers (n = 16) and normal smokers (n = 14). This analysis showed strong separation of smokers from nonsmokers based on expression of the smoking signature genes (Figure 1C). In addition, both singular value decomposition (SVD) and prediction analysis of microarrays (PAM) were used to independently select genes differentially expressed between smokers and nonsmokers (Figure 1D). Genes identified by these methods showed significant overlap with the initial 647 probe sets identified by t-test. Of 300 probe sets identified by SVD, 215 (72%) overlapped with the t-test probe sets, including 96 of the top 100 genes identified by SVD. Of 190 probe sets identified by PAM, 188 (99%) were identified by t-test.

TaqMan real-time RT-PCR was used to confirm expression levels of six representative smoking-responsive genes identified in the healthy smoker *vs* nonsmoker analysis, including cytochrome P450, family 1, subfamily B, polypeptide 1 (CYP1B1), transcription factor 7-like 1 (TCF7L1), aldo-keto reductase family 1, member B10 (AKR1B10), ubiquitin carboxyl-terminal esterase L1 (UCHL1), calcitonin-related polypeptide alpha (CALCA) and NAD(P)H dehydrogenase, quinone 1 (NQO1). For each, RT-PCR confirmed the smoking-induced change in expression observed using the microarrays (Table S3).

To evaluate the difference between the smoking response of the small airways compared to the large airways, the 375-gene smoking signature was evaluated for overlap with other published smoking signature gene lists from the large airway epithelium. Spira et al [19] described genes differentially expressed in the large airway epithelium of 34 current smokers and 23 never smokers. Of the 375 SAE smoking signature genes, 38 (10%) were also present in the Spira et al [19] large airway epithelium analysis (Table S3). Zhang et al [39] described 145 unique genes differentially expressed in the large airway epithelium of 56 current smokers *vs* 24 former smokers, and 92 genes differentially expressed in 56 current *vs* 19 never smokers. Of the 375 SAE smoking signature genes, 53 (14%) were present in current/former smoker analysis, and 46 (12%) were identified in the current/never smoker analysis (Table S4). Thus, while there is some overlap among these studies, the SAE transcriptome in healthy smokers and healthy nonsmokers revealed a large set of novel genes different from that observed in the large airway epithelium.

### Variability of Gene Expression in Healthy Smokers

Significant variability in gene expression was seen among the healthy smokers (Figure S1). For example, for protein phosphatase 1, regulatory (inhibitor) subunit 16B (PPP1R16B), the variance of the log_2_-transformed expression values in healthy smokers was 1.8 *vs* 0.4 for nonsmokers (p<0.0001); for chondroitin sulfate N-acetylgalactosaminyltransferase 1 (CSGALNACT1), the variance in healthy smokers was 1.1 *vs* 0.5 for nonsmokers (p<0.01); for glutathione peroxidase 2 (GPX2), the variance in healthy smokers was 1.0 *vs* 0.3 for nonsmokers (p<0.0002); and for cytochrome P450, family 1, subfamily A, polypeptide 1 (CYP1A1), the variance in healthy smokers was 9.1 *vs* 1.2 for nonsmokers (p<0.0001). Consequent to this variation, each individual healthy smoker expressed some genes above or below 2 standard deviations from the mean of the healthy nonsmokers, but this varied from gene to gene for each individual. In the examples shown, one individual healthy smoker (indicated by the arrow) expressed CSGALNACT1 and CYP1A1 within the range of the nonsmokers, but expressed PPP1R16B and GPX2 outside of the range of the nonsmokers.

### I_SAE_ in Healthy Smokers *vs* Healthy Nonsmokers

**Figure 2:**
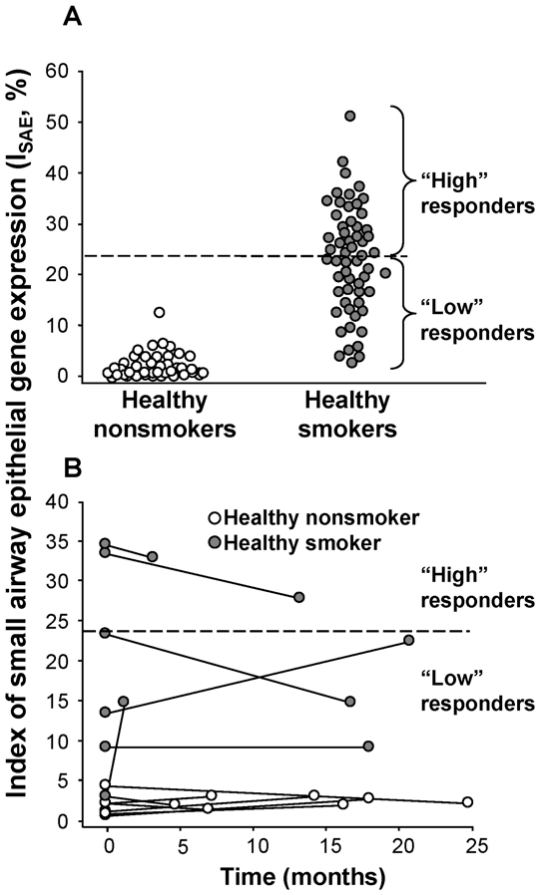
Index for small airway epithelial gene expression (I_SAE_). **A.** I_SAE_ values for nonsmokers (white circles, n = 47) and healthy smokers (gray circles, n = 58). The dashed line demarcates the median I_SAE_ value for the healthy smokers. Individuals with lower I_SAE_ demonstrate abnormal expression of relatively few smoking-responsive genes, and individuals with the highest I_SAE_ values abnormally express the greatest numbers of smoking-responsive genes. Individuals below the median are termed “low” responders and those above the median “high” responders to the stress of smoking. **B.** Assessment of stability of the I_SAE_ over time. Healthy nonsmokers (n = 7, white circles) and healthy smokers (n = 6, gray circles) had assessments of I_SAE_ at time 0 and again at times up to nearly 25 months. All nonsmokers had I_SAE_ values that remained <5%. Among the healthy smokers, 4 low responders remained low responders and 2 high responders remained high responders over time.

The 375 smoking-responsive genes were used to establish the small airway gene expression index (I_SAE_) by assessing for each individual the percent of these genes that were abnormally expressed by that individual. For healthy nonsmokers, the I_SAE_ ranged from 0 to 12.8% (median 1.1%; Figure 2A; Table S5). These index values ranged from 0% (minimum) to 14.7% (maximum) in the K-fold analysis, indicating that the range of I_SAE_ for this group was robust to the effects of sampling. In contrast, healthy smokers demonstrated significantly higher I_SAE_ values, ranging from 2.9% to 51.5% (median 23.6%, p<0.0001 healthy smokers *vs* nonsmokers; Figure 2A; Table S4). The range of these values was likewise robust to sampling effects, from 1.9% (minimum) to 60.0% (maximum) in the K-fold analysis, in which I_SAE_ values were computed using normal gene expression ranges calculated from subgroups of nonsmokers. The variability in I_SAE_ among healthy smokers was significantly greater than that among nonsmokers (variance for healthy smokers 109.4, for nonsmokers 5.8, p<0.0001). For further analysis, the healthy smokers were divided by I_SAE_ value into low responders, those with I_SAE_ values at or below the median, and high responders, those with I_SAE_ values above the median.

There was no relationship of the I_SAE_ values with gender, ancestry, pack-yr smoked, smoking duration in years, FEV1, FEV1/FVC, or FEF25–75. There was a relationship between the I_SAE_ of the healthy nonsmoker and healthy smoker groups and age using a Kruskal-Wallis test (p<0.02), but a linear regression model was not significant (p>0.1) and the amount of variation in I_SAE_ with age was minimal (<3%) and thus it is unlikely that age was an important factor influencing the observed trends. Assessment of possible effects of hybridization date among healthy nonsmokers and healthy smokers using a Kruskal-Wallis test showed no significance. In addition, demographic information, smoking-related parameters, lung function parameters, numbers of cells collected and differential cell counts were examined for low responder *vs* high responder healthy smokers (Table S6; data for pack-yr and years of smoking is shown in Figure S2). There were no statistically significant differences in the two groups for any of these parameters adjusting for multiple testing.

### I_SAE_ Stability Over Time

To evaluate the stability of the I_SAE_ over time, a subset of nonsmokers (n = 7) and healthy smokers (n = 6) underwent repeat bronchoscopies at times ranging from 1 to 25 months (13±7 months) and I_SAE_ was calculated at these subsequent time points (Figure 2B). All nonsmokers' I_SAE_ values at time 0 were <5%, and I_SAE_ remained <5% for each nonsmoker at the repeat bronchoscopy. Among the healthy smokers, 4 individuals were low responders at time 0 and remained low responders at the 2^nd^ evaluation, and 2 individuals were high responders at time 0 and remained high responders at the 2^nd^ evaluation.

### I_SAE_ Allows Meaningful Phenotyping of Healthy Smokers

**Figure 3:**
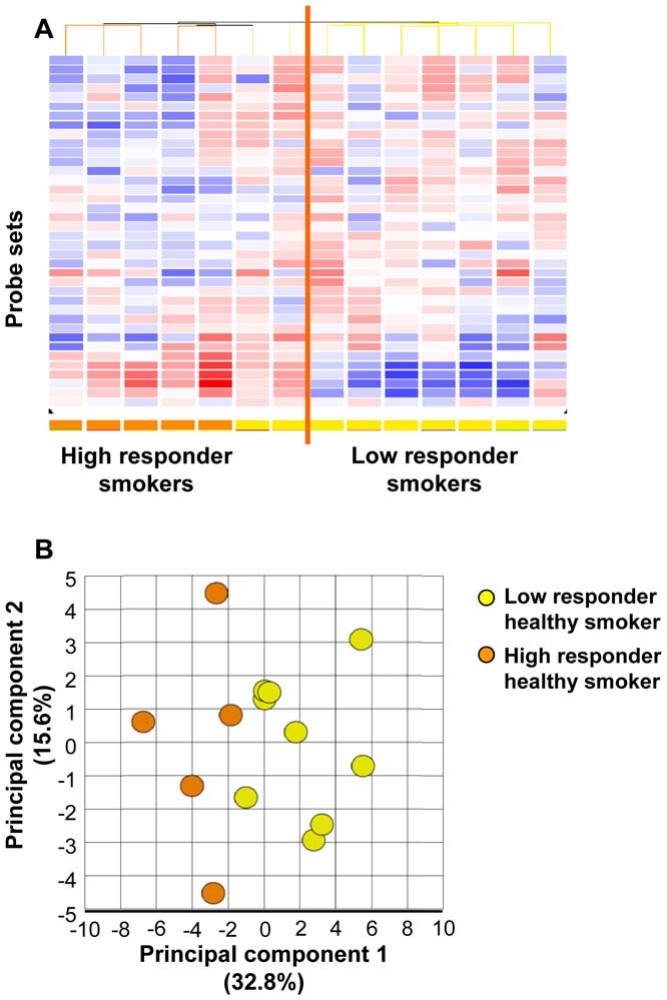
Genome-wide gene expression in the small airway epithelium of high *vs* low responder healthy smokers. Differentially expressed genes were evaluated in the primary set of healthy smokers (n = 29 in each group) and the signature evaluated in the validation set of healthy smokers (n = 14 total; 9 low responders and 5 high responders based on the median I_SAE_ observed in the primary analysis). **A.** Cluster plot. Probe sets expressed above average are represented in red, below average in blue, and average in white. Each row represents one probe set, or gene, and each column represents an individual subject. High responder healthy smokers are indicated by orange, low responder healthy smokers by yellow. **B.** Principal components analysis of gene expression in high responder and low responder healthy smokers. Each axis represents one principal component (PC), with PC1 on the x axis and PC2 on the y axis. Low responder healthy smokers are represented by yellow dots and high responder healthy smokers by orange dots.

To evaluate whether high responder smokers represent a group with small airway epithelial gene expression that is clearly distinct from that of low responder smokers, genome-wide expression analysis (i.e., using all genes, not only the smoking-responsive genes) was used to compare these two groups. Thirty-eight probe sets representing 29 genes were found to be significantly differentially expressed. As expected, 26 probe sets (representing 21 genes) were members of the smoking-responsive set of genes used to categorize smokers as low and high responders. However, this analysis also revealed 12 independent probe sets representing 8 genes significantly differentially expressed in the two groups (Table S7). Cluster analysis using the 38 significant probe sets showed clear separation of the independent group of high *vs* low responder smokers in the validation set (n = 14; 9 low responders and 5 high responders) (Figure 3A). Principal components analysis was consistent with cluster analysis and also showed clear separation of the high and low responders from the independent test set (Figure 3B). The K-fold analysis of these groups indicated that individual classification was robust to sampling effects, with 90% of smoking individuals consistently classified as “high” or “low” responders in >75% of samples.

To evaluate whether the classification of high and low responder smokers was robust to the analytic method used, we assessed three additional methods to subcategorize healthy smokers and evaluated whether those classifications were similar to the index-based classification. First, singular value decomposition was performed using all 647 smoking-responsive probe sets as a principal components analysis on the 58 smokers. In this analysis, the first principal component corresponded to the separation of the high and low responder smokers. These two groups showed significant separation (p<0.0001) and this principal component captured 26.7% of the variability in the data. Second, prediction analysis of microarrays (PAM) was carried out using the 647 smoking-responsive probe sets for all healthy smokers. This assessment showed correct classification of 79.5% of samples, which could be increased to 87.5% accuracy using a reduced centroid of 53 genes. Finally, we performed a support vector machine (SVM) analysis and were able to categorize high and low responder smokers with 76.0% accuracy.

### Comparison of Small Airway Epithelial Gene Expression in Healthy Smokers *vs* COPD Smokers

**Figure 4:**
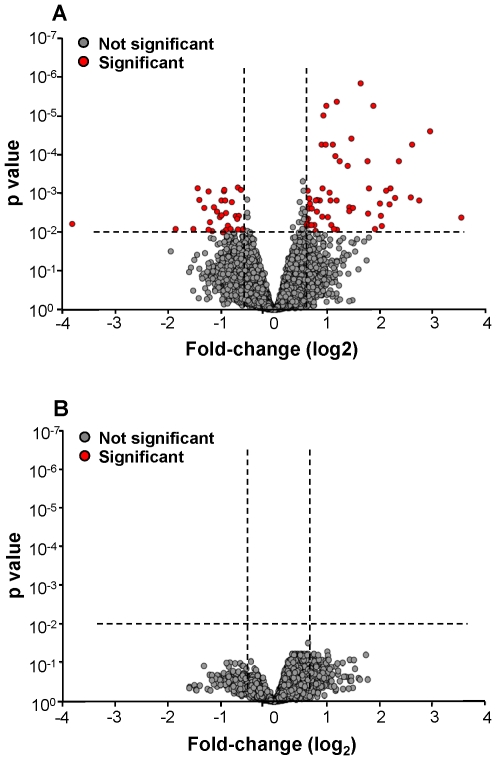
Genome-wide gene expression in the small airway epithelium of COPD smokers (n = 22) *vs* high and low responder subgroups of healthy smokers (n = 29 in each group). **A.** Volcano plot of COPD smokers *vs* low responder healthy smokers. The mean expression level for COPD smokers *vs* low responder healthy smokers was assessed for fold-change (abscissa) *vs* p value (ordinate) by t-test. Each probe set is represented by a filled circle, with probe sets that are not significantly different in COPD smokers compared to low responder healthy smokers in gray and those that are significantly different in the 2 groups in red. Probe sets with a higher expression level in COPD smokers are to the top right and those with a lower expression level in COPD smokers are to the top left. There are 92 probe sets representing 75 known genes that are significantly up- and down-regulated in COPD smokers *vs* low responder healthy smokers. **B.** Volcano plot of COPD smokers *vs* high responder healthy smokers. The mean expression level for COPD smokers *vs* high responder healthy smokers was assessed for fold-change (abscissa) *vs* p value (ordinate) by t-test. Probe sets that are not significantly different in COPD smokers compared to high responder healthy smokers are shown in gray and those that are significantly different in the 2 groups in red. Probe sets with a higher expression level in COPD smokers are to the top right and those with a lower expression level in COPD smokers are to the top left. Genome-wide, no probe sets were significantly different in COPD smokers compared to high responder healthy smokers.

Global gene expression was compared between COPD smokers and the two subgroups of healthy smokers, low and high responders (Figure 4). When COPD smokers were compared to only the subgroup of low responder smokers, a total of 92 probe sets, representing 75 genes, were significantly differentially expressed (Figure 4A, Table S8). In marked contrast, analysis of COPD smokers *vs* high responder smokers revealed no differences in genome-wide gene expression (Figure 4B). This trend was also reflected in the I_SAE_ of COPD smokers, as 88.0% of COPD smokers had an I_SAE_ value within the range of high responder smokers, and 95.5% were similarly classified in >75% of the K-fold samples.

Based on the finding that COPD smokers had gene expression in the small airway similar to high responder smokers, and on the knowledge that only 15 to 20% of smokers develop COPD, genome-wide gene expression was also compared for COPD smokers *vs* the top 20% of smokers based on I_SAE_, and for COPD smokers *vs* the bottom 20% of smokers based on I_SAE_. Consistent with the analysis with the high and low responders based on separation by the median, there were no significant differences between COPD smokers and the top 20% of healthy smokers based on I_SAE_. The analysis of expression of COPD smokers *vs* the bottom 80 20% of smokers revealed 85 probe sets representing 65 unique genes significantly differentially expressed between these two groups (Table S8). This observation was replicated in an independent test set of COPD smokers (n = 14) compared to high responder smokers (n = 5) and low responder smokers (n = 5). While this test set had reduced power compared to the larger primary set, consistent with the data from the primary set, there were no expression differences between the COPD smokers and high responder healthy smokers, whereas 18 probe sets representing 16 genes were differentially expressed between COPD smokers and low responder healthy smokers.

To evaluate whether gene expression can be used as a biomarker to distinguish low responder smokers from high responder and COPD smokers, we combined high responder and COPD smokers and compared genome-wide gene expression in this group *vs.* in the low responder healthy smokers. This analysis revealed 294 probe sets representing 238 unique genes significantly differentially expressed between these two groups (Table S9). Cluster analysis was carried out using this 294 probe set signature on the independent validation set of healthy smokers (n = 14; n = 5 high responders and n = 9 low responders) and COPD smokers (n = 14). The cluster analysis showed no separation between high responder smokers and COPD smokers and separation of those groups from the low responder smokers, with the exception of one high responder smoker who clustered with low responders, and one low responder who clustered with high responder/COPD smokers (Figure S3A). Similarly, principal components analysis on the independent test set using this 294 probe set signature showed overlap of high responder and COPD smokers with this combined group clearly separating from the low responder healthy smokers (Figure S3B).

## Discussion

Cigarette smoking is the major cause of COPD, yet only a minority of smokers develop the disease. Based on the knowledge that cigarette smoking can induce changes in the expression of hundreds of genes in the airway epithelium [18]–[22], and that the small airway epithelium is the earliest site of smoking-induced COPD [21], we hypothesized that gene expression in the small airway epithelium could be used to construct a biologic phenotype that quantifies the individual smoker's response to cigarette smoking. The analysis identified genes that distinguished healthy smokers from nonsmokers and validated that smoking signature using unsupervised cluster analysis on an independent group of samples, as well as by using singular value decomposition and prediction analysis of microarrays methods to independently select smoking signature genes and confirm that the smoking signature genes were nearly the same regardless of method. The data demonstrate that although healthy smokers clearly segregate from healthy nonsmokers, the healthy smokers have variable gene expression patterns. To quantify this observed variability, we developed an index of small airway epithelial gene expression (I_SAE_) that quantifies the number of smoking-responsive genes up- or down-regulated in any given individual. The I_SAE_ separates healthy smokers from healthy nonsmokers and numerically captures the considerable variability in gene expression in the small airway epithelium among healthy smokers. This permits identification of “high” responders to smoking, who show up- and down-regulation of hundreds of genes, and “low” responders with gene expression profiles closer to those of nonsmokers. These categories were robust to the effects of sampling, and reassessment of a subset of these individuals showed stability of the small airway epithelial transcriptome for each individual over time. Genome-wide analysis of the small airway epithelium of high *vs* low responding healthy smokers revealed differences in both smoking-responsive and smoking-independent genes. Interestingly, genome-wide analysis of COPD smokers *vs* low responder healthy smokers identified differences in expression of a significant number of genes, but genome-wide analysis of COPD smokers *vs* high responder healthy smokers identified no significant differences in expression, i.e., the small airway epithelial transcriptomes of COPD smokers and high responder healthy smokers are indistinguishable. This was corroborated by analysis in the independent test set of COPD smokers and healthy smokers, although that data set was smaller and had less power to detect differences. Because of this limitation, we further corroborated this finding by constructing a gene expression biomarker in the primary set to differentiate low responder healthy smokers from COPD and high responder smokers, and used that signature to differentiate these groups in the independent test set of subjects. Although it will take long term followup of large numbers of healthy smokers over many years to determine the fate of “high” and “low” responder healthy smokers, the data demonstrate that analysis of the small airway epithelial transcriptome can be used to subclassify clinically healthy smokers into biologic phenotypes with lesser and greater responses to the insult of cigarette smoke, even though these subgroups are indistinguishable by conventional clinical criteria.

### Smoking and the Airway Epithelial Transcriptome

Several studies have demonstrated that smoking significantly affects the transcriptome of the airway epithelium. Most studies have used the large airway epithelium (0 to 5^th^ generations) as the source of the biologic material. Spira et al [19], and studies from our laboratory [18], have shown variable up- and down-regulation of a number of genes in the large airway epithelium of smokers compared to nonsmokers, while Zhang et al [39] described differences in gene expression in the large airway epithelium of current smokers *vs* both never smokers and former smokers. Beane et al [20] showed that while the large airway epithelial expression of many smoking-responsive genes is reversible upon smoking cessation, there are a number of smoking-responsive genes with persistently abnormal expression after smoking cessation. Pierrou et al [22] found significant changes in the expression of oxidant-related genes in the large airway epithelium of nonsmokers, healthy smokers and COPD smokers, as did the study by Hackett et al [18] of nonsmokers *vs* healthy smokers. A number of studies have examined gene expression in whole lung samples of individuals with COPD, rather than airway epithelium *per se*. Ning et al [40] used both serial analysis of gene expression and microarray technology to detect gene expression differences between GOLD-2 and GOLD-0 lung. Wang et al [41] found altered expression of genes relating to tissue remodeling and repair in samples of lung parenchyma of individuals with COPD compared to nonsmokers. Spira et al [30] and Golpon et al [42] identified changes in gene expression in emphysematous lung tissue compared to normal or mildly emphysematous lung. Finally, the use of airway epithelial gene expression as a biomarker for cancer risk has been explored by Spira et al [43], who developed an 80-gene biomarker that distinguishes between smokers with and without lung cancer based on large airway epithelial gene expression patterns.

While all of these large airway epithelial and whole lung transcriptome studies provide useful information, the small airway epithelium (airways <2 mm, 6^th^ to 23^rd^ generations) is the site of the earliest abnormalities associated with smoking relevant to COPD, including morphologic changes and alterations of cell cycle, repair, apoptosis and response to oxidative stress [5], [7], [15]–[17]. Hogg et al [5], [44] have shown that the small airways are the earliest site of morphologic changes in COPD, and that progression of COPD is strongly associated with local changes in the small airways. Therefore, in strategizing to develop a biologic phenotype to subcategorize healthy smokers we assessed the transcriptome of the small airway epithelium, the site where the disease begins [21].

### I_SAE_ as a Biologic Phenotype

The I_SAE_ is a metric of gene expression for the small airway epithelium that describes, for each individual, the percent of smoking-responsive genes that are abnormally expressed. The I_SAE_ separates healthy smokers from healthy nonsmokers and provides a descriptor by which to quantify the variability in responsiveness to cigarette smoking at the level of small airway epithelial gene expression. Interestingly, some healthy smokers have gene expression profiles quite similar to those of healthy nonsmokers (“low” responders) whereas others have remarkably different patterns of gene expression compared to healthy nonsmokers (“high” responders). The I_SAE_ appears to be robust to the effects of sampling and stable over time, with 6 of 6 healthy smokers retaining their original designation as high or low responders over time periods ranging from 1 to >20 months. When healthy smokers are subgrouped into high and low responders, interesting patterns emerge in genome-wide expression. Significant differences are found between high and low responder healthy smokers, and between COPD smokers and low responder healthy smokers, but no differences are found between COPD smokers and high responder healthy smokers.

The data in the present study support the hypothesis that there is biologic variation at the level of gene expression in the small airway epithelium among a population of healthy smokers, and that a subpopulation of the healthy smokers have a small airway epithelial transcriptome similar to that of smokers with clinical evidence of COPD. If this is true, how can this be reconciled with data showing differences in gene expression in COPD smokers compared to healthy smokers? One explanation, supported by the data in the present study, is that while there are differences with COPD smokers when considering healthy smokers as a homogenous group, when the healthy smokers are subgrouped as high responders and low responders, there are differences only between the COPD smokers and low responders, but not between COPD smokers and high responders. Consistent with this hypothesis, we re-analyzed data from the subjects in a previous publication in which we demonstrated differences in expression of genes related to the Notch pathway between healthy smokers and smokers with COPD [29]. Calculation of I_SAE_ in this population revealed that the majority (60%) of the healthy smoker population were low responders, who would be expected to have greater differences in gene expression compared to COPD smokers than would high responder smokers.

Caveats do apply to our study. First, while the I_SAE_ appears to be robust to the effects of sampling, and consistent in the subset of individuals reevaluated at a later time, to our knowledge, there are no other publicly available data sets of gene expression in the small airway epithelium, and thus our observations will need to be replicated by other investigators. Second, the I_SAE_, reflecting only gene expression changes in epithelial cells, likely does not capture all of the biology of COPD, which involves other cell types, including endothelial cells and inflammatory cells beneath the epithelial basement membrane. However, since small airway epithelial cells show the first morphologic changes relevant to COPD [5], [44], we chose to develop the I_SAE_ based on gene expression changes in those cells. Finally, the concept that high responder smokers might be at higher risk for COPD is a hypothesis; proof will require large numbers of subjects to be followed for decades. It has recently been suggested that variations in individual responses to cigarette smoking may underlie the different clinical and molecular phenotypes and variable natural history associated with COPD [45]. We believe this to be true, and we suggest that the small airway epithelial gene expression phenotype quantified in the I_SAE_ may have biologic significance, i.e., that the group of smokers that manifests the highest response of gene expression in the small airway epithelium, though clinically healthy, are biologically different from individuals with low responses to smoking, may respond to therapy differently, and may have different prognoses. In this context, the I_SAE_ represents a tool for characterizing phenotype among smokers that could be prospectively examined in epidemiologic studies. This may prove useful for risk assessment and prognosis for individual patients, as well as in therapeutic trials as a surrogate outcome measure.

## Supporting Information

Figure S1 **Examples of variable response of the human small airway epithelium to smoking.** Arrows indicate how this is used to construct the index for small airway epithelium gene expression (I_SAE_). Each circle represents log_2_ transformed gene expression for one individual, with healthy nonsmokers (n = 47) on the left and healthy smokers (n = 58) on the right in each graph. The gray shaded area represents the mean expression value in healthy nonsmokers ±2 standard deviations. Open circles represent expression values within the 2 standard deviations of the mean in healthy nonsmokers, which did not contribute to the overall I_SAE_ score. Black circles represent values considered abnormal, i.e., more than 2 standard deviations from the mean, in the direction of the smoking-induced change, and which did contribute to the I_SAE_. As an example of how the data were used to calculate the I_SAE_, one healthy smoker is indicated by an arrow in each of the 4 panels, representing how that individual expressed the 4 genes chosen as examples. **A.** Expression of protein phosphatase 1, regulatory (inhibitor) subunit 16B (PPP1R16B). The healthy smoker marked with the arrow has abnormal expression for this gene and received a “1” toward the I_SAE_. **B.** Expression of chondroitin sulfate N-acetylgalactosaminyltransferase 1 (CSGALNACT1). The representative healthy smoker (arrow) had normal expression for this gene and thus had a “0” toward the index for this gene. **C.** Expression of glutathione peroxidase 2 (GPX2). The representative healthy smoker (arrow) had abnormal expression for this gene and thus had a “1” toward the index for this gene. **D.** Expression of cytochrome P450, family 1, subfamily A, polypeptide 1 (CYP1A1). The representative individual (arrow) had normal expression for this gene and received a “0” toward the index. Note that this healthy smoker individual has normal expression within 2 standard deviations of the mean in healthy nonsmokers for CSGALNACT1 and CYP1A1, but abnormal expression for PPP1R16B and GPX2.
(TIFF)

Figure S2 **Distribution of smoking exposure parameters in low responder and high responder healthy smokers.** The abscissa displays the two groups. Each individual is represented by a black diamond. **A.** Smoking history in pack-yr is represented on the ordinate. There is no significant difference between the two groups for pack-yr (p>0.1). **B.** Smoking duration in years is represented on the ordinate. There is no significant difference between the two groups for years of smoking (p>0.2).
(TIF)

Figure S3 **Genome-wide gene expression in the small airway epithelium of high responder and COPD smokers** ***vs.*** **low responder healthy smokers.** Differentially expressed genes were evaluated in the primary set of subjects (n = 51 in the combined high responder/COPD group; n = 29 low responder healthy smokers) and the signature evaluated in the independent validation set (n = 9 low responder healthy smokers, n = 5 high responder healthy smokers, n = 14 COPD smokers). **A.** Cluster plot. Probe sets expressed above average are represented in red, below average in blue, and average in white. Each row represents one probe set, or gene, and each column represents an individual subject. COPD smokers are represented by red, high responder healthy smokers by orange, and low responder healthy smokers by yellow. **B.** Principal components analysis of gene expression in COPD smoker, high responder and low responder healthy smokers. Each axis represents one principal component (PC), with PC1 on the x axis and PC2 on the y axis. Low responder healthy smokers are represented by yellow dots, high responder healthy smokers by orange dots and COPD smokers by red dots.
(TIF)

Table S1 **Identity of validation set subjects.**
(DOC)

Table S2 **Smoking-related differentially expressed genes in the small airway epithelium of healthy nonsmokers and healthy smokers.**
(DOC)

Table S3 **TaqMan confirmation of selected genes.**
(DOC)

Table S4 **Overlap of genes differentially expressed in the SAE of healthy smokers** ***vs*** **nonsmokers with other reported smoking responsive genes.**
(DOC)

Table S5 **Characteristics of the I_SAE_ among the study population.**
(DOC)

Table S6 **Demographics of low responder and high responder healthy smokers.**
(DOC)

Table S7 **Genes differentially expressed in the small airway epithelium of high responder healthy smokers** ***vs*** **low responder healthy smokers.**
(DOC)

Table S8 **Genes differentially expressed in the small airway epithelium of COPD smokers** ***vs*** **low responder healthy smokers.**
(DOC)

Table S9 **Genes differentially expressed in the small airway epithelium of COPD smokers and high responder smokers** ***vs*** **low responder smokers.**
(DOC)

Text S1 (DOC)

## References

[1] MacNee W (2000). Oxidants/antioxidants and COPD.. Chest.

[2] MacNee W (2001). Oxidative stress and lung inflammation in airways disease.. Eur J Pharmacol.

[3] Hecht SS (2003). Tobacco carcinogens, their biomarkers and tobacco-induced cancer.. Nat Rev Cancer.

[4] Yoshida T, Tuder RM (2007). Pathobiology of cigarette smoke-induced chronic obstructive pulmonary disease.. Physiol Rev.

[5] Hogg JC (2004). Pathophysiology of airflow limitation in chronic obstructive pulmonary disease.. The Lancet.

[6] Puchelle E, Zahm JM, Tournier JM, Coraux C (2006). Airway epithelial repair, regeneration, and remodeling after injury in chronic obstructive pulmonary disease.. Proc Am Thorac Soc.

[7] Hogg JC, Macklem PT, Thurlbeck WM (1968). Site and nature of airway obstruction in chronic obstructive lung disease.. N Engl J Med.

[8] Saetta M, Finkelstein R, Cosio MG (1994). Morphological and cellular basis for airflow limitation in smokers.. Eur Respir J.

[9] Thompson AB, Robbins RA, Romberger DJ, Sisson JH, Spurzem JR (1995). Immunological functions of the pulmonary epithelium.. Eur Respir J.

[10] Maestrelli P, Saetta M, Mapp CE, Fabbri LM (2001). Remodeling in response to infection and injury . Airway inflammation and hypersecretion of mucus in smoking subjects with chronic obstructive pulmonary disease.. Am J Respir Crit Care Med.

[11] Fletcher C, Peto R (1977). The natural history of chronic airflow obstruction.. Br Med J.

[12] Sethi JM, Rochester CL (2000). Smoking and chronic obstructive pulmonary disease.. Clin Chest Med.

[13] Rabe KF, Hurd S, Anzueto A, Barnes PJ, Buist SA (2007). Global strategy for the diagnosis, management, and prevention of chronic obstructive pulmonary disease: GOLD executive summary.. Am J Respir Crit Care Med.

[14] Shapiro SD, Ingenito EP (2005). The pathogenesis of chronic obstructive pulmonary disease: advances in the past 100 years.. Am J Respir Cell Mol Biol.

[15] Barnes PJ, Shapiro SD, Pauwels RA (2003). Chronic obstructive pulmonary disease: molecular and cellular mechanisms.. Eur Respir J.

[16] Sethi S, Maloney J, Grove L, Wrona C, Berenson CS (2006). Airway inflammation and bronchial bacterial colonization in chronic obstructive pulmonary disease.. Am J Respir Crit Care Med.

[17] Curtis JL, Freeman CM, Hogg JC (2007). The immunopathogenesis of chronic obstructive pulmonary disease: insights from recent research.. Proc Am Thorac Soc.

[18] Hackett NR, Heguy A, Harvey BG, O'Connor TP, Luettich K (2003). Variability of antioxidant-related gene expression in the airway epithelium of cigarette smokers.. Am J Respir Cell Mol Biol.

[19] Spira A, Beane J, Shah V, Liu G, Schembri F (2004). Effects of cigarette smoke on the human airway epithelial cell transcriptome.. Proc Natl Acad Sci U S A.

[20] Beane J, Sebastiani P, Liu G, Brody JS, Lenburg ME (2007). Reversible and permanent effects of tobacco smoke exposure on airway epithelial gene expression.. Genome Biol.

[21] Harvey BG, Heguy A, Leopold PL, Carolan BJ, Ferris B (2007). Modification of gene expression of the small airway epithelium in response to cigarette smoking.. J Mol Med.

[22] Pierrou S, Broberg P, O'Donnell RA, Pawlowski K, Virtala R (2007). Expression of genes involved in oxidative stress responses in airway epithelial cells of smokers with chronic obstructive pulmonary disease.. Am J Respir Crit Care Med.

[23] Tilley AE, O'Connor TP, Hackett NR, Zhou XK, Strulovici Y (2008). Variability of the small airway epithelium gene expression in response to cigarette smoke among the healthy population and individuals with COPD.. Am J Respir Crit Care Med.

[24] Ammous Z, Hackett NR, Butler MW, Raman T, Dolgalev I (2008). Variability in small airway epithelial gene expression among normal smokers.. Chest.

[25] Carolan BJ, Harvey BG, De BP, Vanni H, Crystal RG (2008). Decreased expression of intelectin 1 in the human airway epithelium of smokers compared to nonsmokers.. J Immunol.

[26] Carolan BJ, Harvey BG, Hackett NR, O'Connor TP, Cassano PA (2009). Disparate oxidant gene expression of airway epithelium compared to alveolar macrophages in smokers.. Respir Res.

[27] Hubner RH, Schwartz JD, De BP, Ferris B, Omberg L (2009). Coordinate control of expression of Nrf2-modulated genes in the human small airway epithelium is highly responsive to cigarette smoking.. Mol Med.

[28] Raman T, O'Connor TP, Hackett NR, Wang W, Harvey BG (2009). Quality control in microarray assessment of gene expression in human airway epithelium.. BMC Genomics.

[29] Tilley AE, Harvey BG, Heguy A, Hackett NR, Wang R (2009). Down-regulation of the notch pathway in human airway epithelium in association with smoking and chronic obstructive pulmonary disease.. Am J Respir Crit Care Med.

[30] Spira A, Beane J, Pinto-Plata V, Kadar A, Liu G (2004). Gene expression profiling of human lung tissue from smokers with severe emphysema.. Am J Respir Crit Care Med.

[31] DeRuisseau KC, Shanely RA, Akunuri N, Hamilton MT, van Gammeren D (2005). Diaphragm unloading via controlled mechanical ventilation alters the gene expression profile.. Am J Respir Crit Care Med.

[32] Lu BS, Yu AD, Zhu X, Garrity ER, Vigneswaran WT (2006). Sequential gene expression profiling in lung transplant recipients with chronic rejection.. Chest.

[33] Kramer EL, Deutsch GH, Sartor MA, Hardie WD, Ikegami M (2007). Perinatal increases in TGF-{alpha} disrupt the saccular phase of lung morphogenesis and cause remodeling: microarray analysis.. Am J Physiol Lung Cell Mol Physiol.

[34] Bittel DC, Kibiryeva N, Butler MG (2007). Whole genome microarray analysis of gene expression in subjects with fragile X syndrome.. Genet Med.

[35] Miller WR, Larionov AA, Renshaw L, Anderson TJ, White S (2007). Changes in breast cancer transcriptional profiles after treatment with the aromatase inhibitor, letrozole.. Pharmacogenet Genomics.

[36] Benjamini Y, Hochberg Y (1995). Controlling the false discovery rate: a practical and powerful approach to multiple testing.. J R Stat Soc.

[37] Alter O, Golub GH (2006). Singular value decomposition of genome-scale mRNA lengths distribution reveals asymmetry in RNA gel electrophoresis band broadening.. Proc Natl Acad Sci U S A.

[38] Tibshirani R, Hastie T, Narasimhan B, Chu G (2002). Diagnosis of multiple cancer types by shrunken centroids of gene expression.. Proc Natl Acad Sci U S A.

[39] Zhang LI, Lee J, Tang H, Fan YH, Xiao L (2008). Impact of smoking cessation on global gene expression in the bronchial epithelium of chronic smokers.. Cancer Prev Res.

[40] Ning W, Lee J, Kaminski N, Feghali-Bostwick CA, Watkins SC (2006). Comprehensive analysis of gene expression on GOLD-2 Versus GOLD-0 smokers reveals novel genes important in the pathogenesis of COPD.. Proc Am Thorac Soc.

[41] Wang IM, Stepaniants S, Boie Y, Mortimer JR, Kennedy B (2008). Gene expression profiling in patients with chronic obstructive pulmonary disease and lung cancer.. Am J Respir Crit Care Med.

[42] Golpon HA, Coldren CD, Zamora MR, Cosgrove GP, Moore MD (2004). Emphysema lung tissue gene expression profiling.. Am J Respir Cell Mol Biol.

[43] Spira A, Beane JE, Shah V, Steiling K, Liu G (2007). Airway epithelial gene expression in the diagnostic evaluation of smokers with suspect lung cancer.. Nat Med.

[44] Hogg JC, Chu F, Utokaparch S, Woods R, Elliott WM (2004). The nature of small-airway obstruction in chronic obstructive pulmonary disease.. N Engl J Med.

[45] Steiling K, Lenburg ME, Spira A (2009). Airway gene expression in chronic obstructive pulmonary disease.. Proc Am Thorac Soc.

