# Supplementary figures and images for: Biologic Phenotyping of the Human Small Airway Epithelial Response to Cigarette Smoking
Source: PLoS One. 2011 Jul 28;6(7):e22798. doi: 10.1371/journal.pone.0022798 (PMC3145669; doi:10.1371/journal.pone.0022798)

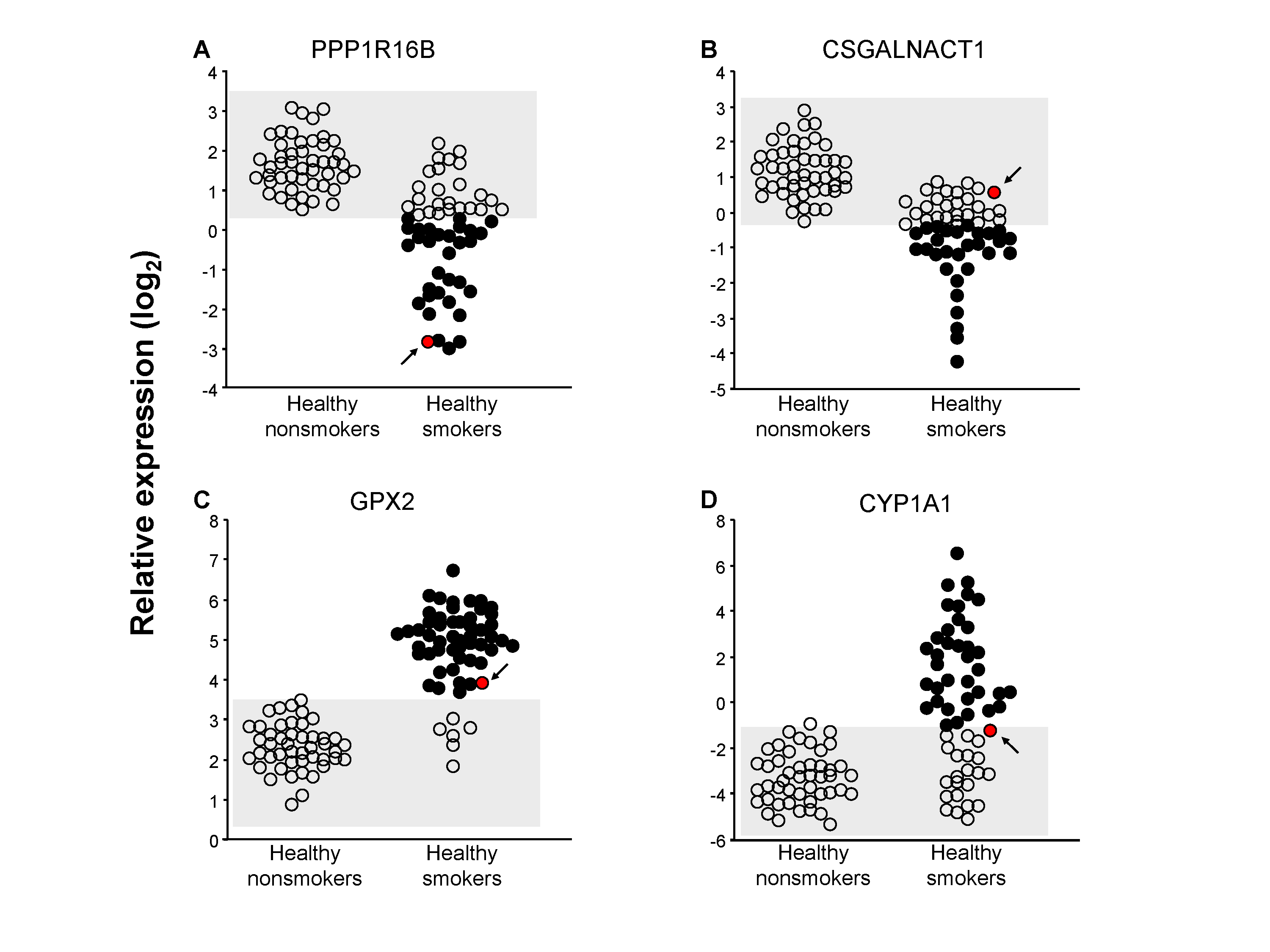

Supplement: Figure S1 — Examples of variable response of the human small airway epithelium to smoking. Arrows indicate how this is used to construct the index for small airway epithelium gene expression (ISAE). Each circle represents log2 transformed gene expression for one individual, with healthy nonsmokers (n = 47) on the left and healthy smokers (n = 58) on the right in each graph. The gray shaded area represents the mean expression value in healthy nonsmokers ±2 standard deviations. Open circles represent expression values within the 2 standard deviations of the mean in healthy nonsmokers, which did not contribute to the overall ISAE score. Black circles represent values considered abnormal, i.e., more than 2 standard deviations from the mean, in the direction of the smoking-induced change, and which did contribute to the ISAE. As an example of how the data were used to calculate the ISAE, one healthy smoker is indicated by an arrow in each of the 4 panels, representing how that individual expressed the 4 genes chosen as examples. A. Expression of protein phosphatase 1, regulatory (inhibitor) subunit 16B (PPP1R16B). The healthy smoker marked with the arrow has abnormal expression for this gene and received a “1” toward the ISAE. B. Expression of chondroitin sulfate N-acetylgalactosaminyltransferase 1 (CSGALNACT1). The representative healthy smoker (arrow) had normal expression for this gene and thus had a “0” toward the index for this gene. C. Expression of glutathione peroxidase 2 (GPX2). The representative healthy smoker (arrow) had abnormal expression for this gene and thus had a “1” toward the index for this gene. D. Expression of cytochrome P450, family 1, subfamily A, polypeptide 1 (CYP1A1). The representative individual (arrow) had normal expression for this gene and received a “0” toward the index. Note that this healthy smoker individual has normal expression within 2 standard deviations of the mean in healthy nonsmokers for CSGALNACT1 and CYP1A1, but abnormal expression for [file pone.0022798.s001.tif]

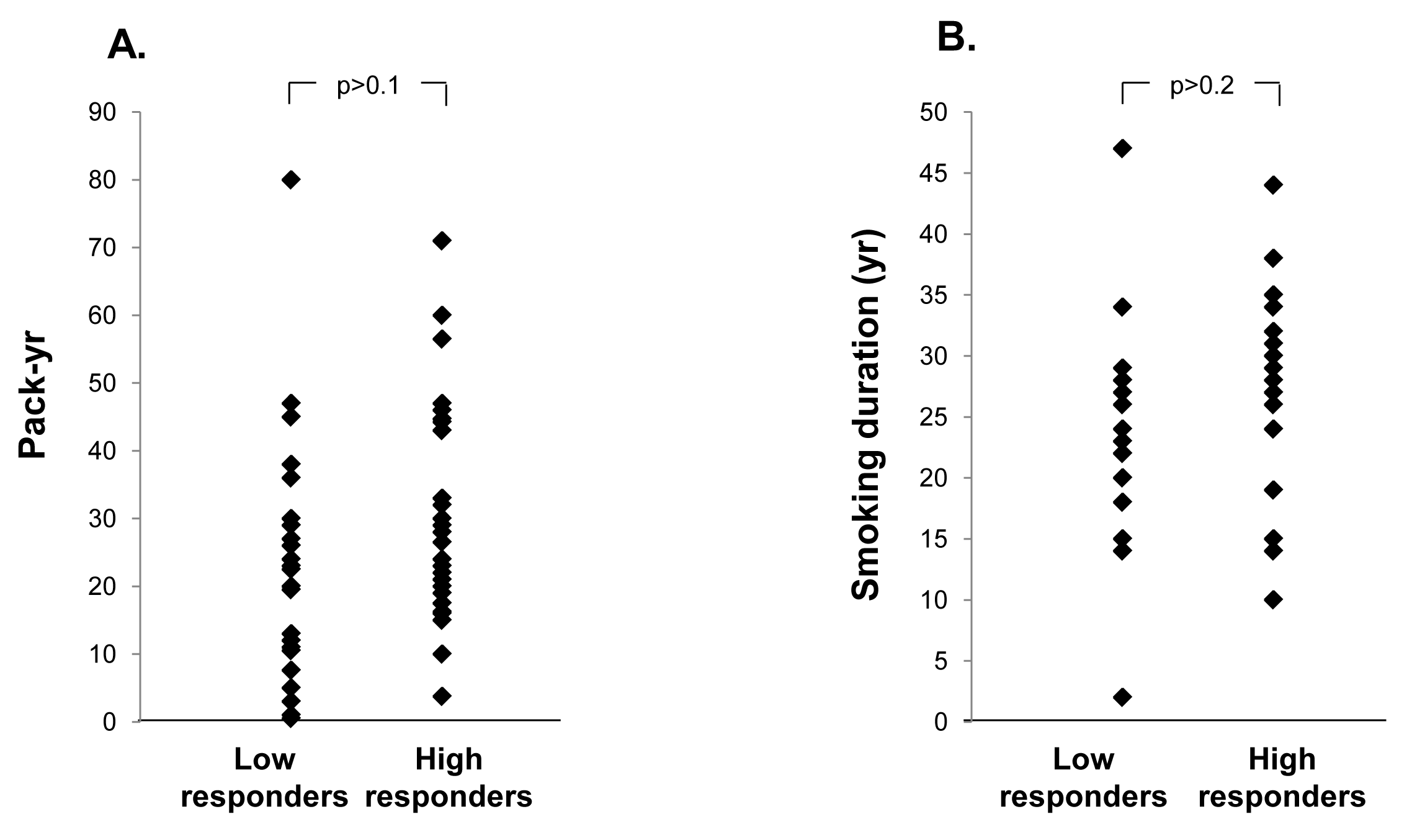

Supplement: Figure S2 — Distribution of smoking exposure parameters in low responder and high responder healthy smokers. The abscissa displays the two groups. Each individual is represented by a black diamond. A. Smoking history in pack-yr is represented on the ordinate. There is no significant difference between the two groups for pack-yr (p>0.1). B. Smoking duration in years is represented on the ordinate. There is no significant difference between the two groups for years of smoking (p>0.2). (TIF) [file pone.0022798.s002.tif]

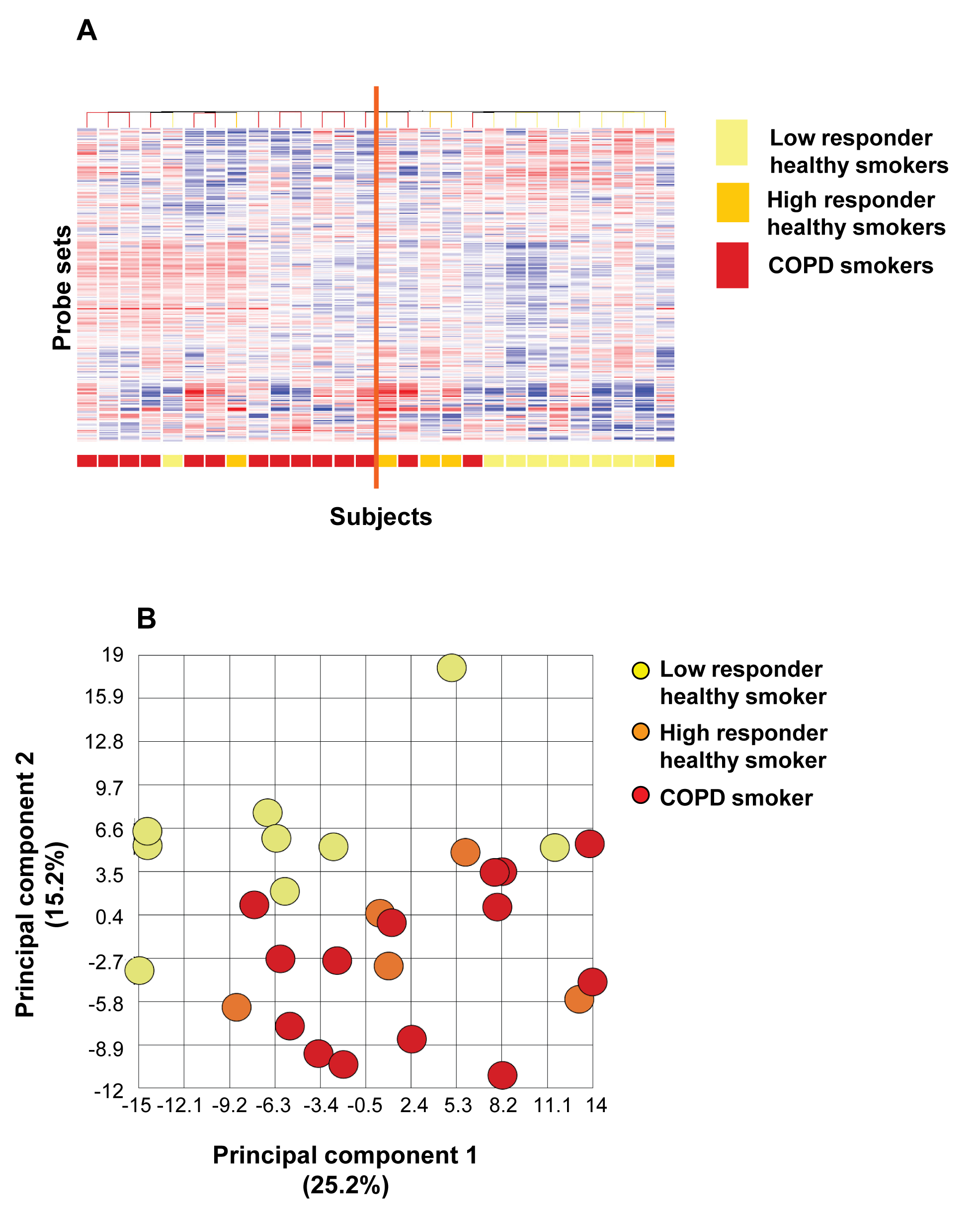

Supplement: Figure S3 — Genome-wide gene expression in the small airway epithelium of high responder and COPD smokers vs. low responder healthy smokers. Differentially expressed genes were evaluated in the primary set of subjects (n = 51 in the combined high responder/COPD group; n = 29 low responder healthy smokers) and the signature evaluated in the independent validation set (n = 9 low responder healthy smokers, n = 5 high responder healthy smokers, n = 14 COPD smokers). A. Cluster plot. Probe sets expressed above average are represented in red, below average in blue, and average in white. Each row represents one probe set, or gene, and each column represents an individual subject. COPD smokers are represented by red, high responder healthy smokers by orange, and low responder healthy smokers by yellow. B. Principal components analysis of gene expression in COPD smoker, high responder and low responder healthy smokers. Each axis represents one principal component (PC), with PC1 on the x axis and PC2 on the y axis. Low responder healthy smokers are represented by yellow dots, high responder healthy smokers by orange dots and COPD smokers by red dots. (TIF) [file pone.0022798.s003.tif]
