# Supplementary material for: Biologic Phenotyping of the Human Small Airway Epithelial Response to Cigarette Smoking
Source: PLoS One. 2011 Jul 28;6(7):e22798. doi: 10.1371/journal.pone.0022798 (PMC3145669; doi:10.1371/journal.pone.0022798)
Supplement: Table S1 — Identity of validation set subjects. (DOC) [file pone.0022798.s004.doc]

**Supplemental Table I. Identity of Validation Set Subjects^1^**

| **GSM Number** | **Subject Identifier** | **Phenotype** |
| --- | --- | --- |
| GSM469989 | nonsmoker 091 | Normal nonsmoker |
| GSM458579 | nonsmoker 132 | Normal nonsmoker |
| GSM410162 | nonsmoker 138 | Normal nonsmoker |
| GSM469990 | nonsmoker 140 | Normal nonsmoker |
| GSM469991 | nonsmoker 147 | Normal nonsmoker |
| GSM469992 | nonsmoker 151 | Normal nonsmoker |
| GSM458580 | nonsmoker 155 | Normal nonsmoker |
| GSM469993 | nonsmoker 156 | Normal nonsmoker |
| GSM458581 | nonsmoker 163 | Normal nonsmoker |
| GSM458582 | nonsmoker 169 | Normal nonsmoker |
| GSM469994 | nonsmoker 175 | Normal nonsmoker |
| GSM469995 | nonsmoker 179 | Normal nonsmoker |
| GSM469996 | nonsmoker 181 | Normal nonsmoker |
| GSM469997 | nonsmoker 193 | Normal nonsmoker |
| GSM469998 | nonsmoker 199 | Normal nonsmoker |
| GSM469999 | nonsmoker 212 | Normal nonsmoker |
| GSM470000 | healthy smoker 206 | Healthy smoker |
| GSM470001 | healthy smoker 215 | Healthy smoker |
| GSM470002 | healthy smoker 220 | Healthy smoker |
| GSM470003 | healthy smoker 269 | Healthy smoker |
| GSM470004 | healthy smoker 276 | Healthy smoker |
| GSM470005 | healthy smoker 321 | Healthy smoker |
| GSM470006 | healthy smoker 327 | Healthy smoker |
| GSM470007 | healthy smoker 328 | Healthy smoker |
| GSM470008 | healthy smoker 330 | Healthy smoker |
| GSM470009 | healthy smoker 341 | Healthy smoker |
| GSM470010 | healthy smoker 353 | Healthy smoker |
| GSM470011 | healthy smoker 354 | Healthy smoker |
| GSM470012 | healthy smoker 377 | Healthy smoker |
| GSM470013 | healthy smoker 392 | Healthy smoker |
| GSM549651 | DGM-00167 [gene_expr] | COPD smoker |
| GSM549667 | DGM-10135 [gene_expr] | COPD smoker |
| GSM549669 | DGM-10144 [gene_expr] | COPD smoker |
| GSM549670 | DGM-10130 [gene_expr] | COPD smoker |
| GSM549672 | DGM-01607 [gene_expr] | COPD smoker |
| GSM549673 | DGM-01737 [gene_expr] | COPD smoker |
| GSM549674 | DGM-01749 [gene_expr] | COPD smoker |
| GSM549675 | DGM-01772 [gene_expr] | COPD smoker |
| GSM549676 | DGM-01081 [gene_expr] | COPD smoker |
| GSM549677 | DGM-01827 [gene_expr] | COPD smoker |
| GSM549678 | DGM-02093 [gene_expr] | COPD smoker |
| GSM549679 | DGM-02109 [gene_expr] | COPD smoker |
| GSM549680 | DGM-02124 [gene_expr] | COPD smoker |
| GSM549782 | DGM-00544 [gene_expr] | COPD smoker |

^1^ GSM numbers and sample identifiers are shown for the subjects used as the validation set for the analyses as described in Methods and Results. The validation set consisted of normal nonsmokers (n=16), healthy smokers (n=14) and COPD smokers (n=14) Demographic data for these subjects is presented in Table 1.
