# Supplementary material for: Biologic Phenotyping of the Human Small Airway Epithelial Response to Cigarette Smoking
Source: PLoS One. 2011 Jul 28;6(7):e22798. doi: 10.1371/journal.pone.0022798 (PMC3145669; doi:10.1371/journal.pone.0022798)
Supplement: Table S2 — Smoking-related differentially expressed genes in the small airway epithelium of healthy nonsmokers and healthy smokers. (DOC) [file pone.0022798.s005.doc]

| **Probe set ID** | **Gene symbol** | **Gene title** | **Fold-change (smoker/nonsmoker)^2^** | **p value^3^** | **P call (%)**^4^ |
| --- | --- | --- | --- | --- | --- |
|  |  |  |  |  |  |
| 223593_at | AADAT | aminoadipate aminotransferase | -1.78 | 6.52 x 10^-11^ | 97 |
| 1553295_at | ABCA13 | ATP-binding cassette, sub-family A (ABC1), member 13 | -1.61 | 1.31 x 10^-4^ | 97 |
| 155364_at | ABCA13 | ATP-binding cassette, sub-family A (ABC1), member 13 | -1.82 | 2.39 x 10^-8^ | 100 |
| 1553605_a_at | ABCA13 | ATP-binding cassette, sub-family A (ABC1), member 13 | -1.73 | 3.91 x 10^-7^ | 100 |
| 203192_at | ABCB6 | ATP-binding cassette, sub-family B (MDR/TAP), member 6 | 1.87 | 2.50 x 10^-9^ | 99 |
| 28161_s_at | ABCC3 | ATP-binding cassette, sub-family C (CFTR/MRP), member 3 | 1.68 | 4.42 x 10^-4^ | 86 |
| 205566_at | ABHD2 | abhydrolase domain containing 2 | 1.59 | 1.21 x 10^-6^ | 100 |
| 221815_at | ABHD2 | abhydrolase domain containing 2 | 1.83 | 7.22 x 10^-6^ | 100 |
| 228490_at | ABHD2 | abhydrolase domain containing 2 | 1.56 | 2.84 x 10^-9^ | 100 |
| 63825_at | ABHD2 | abhydrolase domain containing 2 | 1.65 | 8.43 x 10^-7^ | 100 |
| 87100_at | ABHD2 | abhydrolase domain containing 2 | 1.60 | 6.52 x 10^-3^ | 98 |
| 220013_at | ABHD9 | abhydrolase domain containing 9 | -1.60 | 1.65 x 10^-4^ | 54 |
| 214913_at | ADAMTS3 | ADAM metallopeptidase with thrombospondin type 1 motif, 3 | -1.65 | 7.66 x 10^-4^ | 61 |
| 203865_s_at | ADARB1 | adenosine deaminase, RNA-specific, B1 (RED1 homolog rat) | -1.70 | 1.34 x 10^-7^ | 57 |
| 210505_at | ADH7 | alcohol dehydrogenase 7 (class IV), mu or sigma polypeptide | 6.07 | 1.69 x 10^-24^ | 100 |
| 226955_at | AFAP1L1 | actin filament associated protein 1-like 1 | -1.74 | 6.67 x 10^-8^ | 95 |
| 224480_s_at | AGPAT9 | 1-acylglycerol-3-phosphate O-acyltransferase 9 | 2.21 | 1.02 x 10^-8^ | 100 |
| 228969_at | AGR2 | anterior gradient homolog 2 (Xenopus laevis) | 1.54 | 1.24 x 10^-3^ | 100 |
| 229354_at | AHRR /// PDCD6 | aryl-hydrocarbon receptor repressor /// programmed cell death 6 | 2.64 | 1.12 x 10^-6^ | 50 |
| 26460_at | AJAP1 | adherens junctions associated protein 1 | 1.85 | 3.78 x 10^-3^ | 37 |
| 205771_s_at | AKAP7 | A kinase (PRKA) anchor protein 7 | -1.56 | 3.27 x 10^-8^ | 100 |
| 201272_at | AKR1B1 | aldo-keto reductase family 1, member B1 (aldose reductase) | 1.71 | 6.33 x 10^-9^ | 100 |
| 26561_s_at | AKR1B10 | aldo-keto reductase family 1, member B10 (aldose reductase) | 16.62 | 1.55 x 10^-22^ | 87 |
| 1562102_at | AKR1C1 | Aldo-keto reductase family 1, member C1 (dihydrodiol dehydrogenase 1; 20-alpha (3-alpha)-hydroxysteroid dehydrogenase), mRNA (cDNA clone MGC:42600 IMAGE:4825338) | 1.78 | 2.98 x 10^-3^ | 90 |
| 24151_ x _at | AKR1C1 | aldo-keto reductase family 1, member C1 (dihydrodiol dehydrogenase 1; 20-alpha (3-alpha)-hydroxysteroid dehydrogenase) | 2.39 | 1.82 x 10^-16^ | 100 |
| 216594_ x _at | AKR1C1 | aldo-keto reductase family 1, member C1 (dihydrodiol dehydrogenase 1; 20-alpha (3-alpha)-hydroxysteroid dehydrogenase) | 2.95 | 1.63 x 10^-17^ | 100 |
| 217626_at | AKR1C1 | aldo-keto reductase family 1, member C1 (dihydrodiol dehydrogenase 1; 20-alpha (3-alpha)-hydroxysteroid dehydrogenase) | 3.93 | 7.36 x 10^-13^ | 85 |
| 209699_ x _at | AKR1C2 | aldo-keto reductase family 1, member C2 (dihydrodiol dehydrogenase 2; bile acid binding protein; 3-alpha hydroxysteroid dehydrogenase, type III) | 3.34 | 1.14 x 10^-18^ | 100 |
| 211653_ x _at | AKR1C2 | aldo-keto reductase family 1, member C2 (dihydrodiol dehydrogenase 2; bile acid binding protein; 3-alpha hydroxysteroid dehydrogenase, type III) | 2.77 | 1.50 x 10^-16^ | 100 |
| 209160_at | AKR1C3 | aldo-keto reductase family 1, member C3 (3-alpha hydroxysteroid dehydrogenase, type II) | 2.67 | 8.42 x 10^-15^ | 100 |
| 205623_at | ALDH3A1 | aldehyde dehydrogenase 3 family, memberA1 | 5.34 | 5.40 x 10^-19^ | 100 |
| 28498_s_at | AMY1A /// AMY1B /// AMY1C /// AMY2A /// AMY2B | amylase, alpha 1A (salivary) /// amylase, alpha 1B (salivary) /// amylase, alpha 1C (salivary) /// amylase, alpha 2A (pancreatic) /// amylase, alpha 2B (pancreatic) | -1.52 | 2.67 x 10^-4^ | 100 |
| 20568_s_at | ANGPT1 | angiopoietin 1 | -1.59 | 2.90 x 10^-7^ | 75 |
| 205609_at | ANGPT1 | angiopoietin 1 | -2.56 | 5.54 x 10^-10^ | 50 |
| 215241_at | ANO3 | anoctamin 3 | -1.69 | 4.74 x 10^-4^ | 77 |
| 236420_s_at | ANO4 | anoctamin 4 | -2.80 | 4.40 x 10^-6^ | 39 |
| 202888_s_at | ANPEP | alanyl (membrane) aminopeptidase | -1.64 | 3.34 x 10^-6^ | 100 |
| 225524_at | ANTXR2 | anthrax toxin receptor 2 | -1.55 | 1.89 x 10^-6^ | 100 |
| 24894_s_at | AOC3 | amine oxidase, copper containing 3 (vascular adhesion protein 1) | -1.70 | 6.32 x 10^-3^ | 33 |
| 218218_at | APPL2 | adaptor protein, phosphotyrosine interaction, PH domain and leucine zipper containing 2 | -1.57 | 1.43 x 10^-5^ | 100 |
| 24425_at | ARHGAP4 | Rho GTPase activating protein 4 | -1.64 | 7.43 x 10^-4^ | 31 |
| 233724_at | ARNT | aryl hydrocarbon receptor nuclear translocator | 1.66 | 6.00 x 10^-3^ | 36 |
| 218832_ x _at | ARRB1 | arrestin, beta 1 | -1.94 | 8.22 x 10^-4^ | 37 |
| 221161_at | ASCL3 | achaete-scute complex homolog 3 (Drosophila) | 1.53 | 8.23 x 10^-3^ | 74 |
| 219127_at | ATAD4 | ATPase family, AAA domain containing 4 | -1.53 | 6.93 x 10^-8^ | 100 |
| 207367_at | ATP12A | ATPase, H+/K+ transporting, nongastric, alpha polypeptide | -1.63 | 1.19 x 10^-3^ | 100 |
| 1557136_at | ATP13A4 | ATPase type 13A4 | -1.79 | 1.34 x 10^-7^ | 99 |
| 1559571_a_at | ATP13A4 | ATPase type 13A4 | -1.54 | 1.41 x 10^-3^ | 37 |
| 220197_at | ATP6V0A4 | ATPase, H+ transporting, lysosomal V0 subunit a4 | 1.78 | 5.85 x 10^-7^ | 100 |
| 205473_at | ATP6V1B1 | ATPase, H+ transporting, lysosomal 56/58kDa, V1 subunit B1 | -2.07 | 3.11 x 10^-6^ | 47 |
| 26251_s_at | AVPR1A | arginine vasopressin receptor 1A | -2.02 | 2.56 x 10^-3^ | 33 |
| 209309_at | AZGP1 | alpha-2-glycoprotein 1, zinc-binding | 1.62 | 2.35 x 10^-3^ | 98 |
| 214575_s_at | AZU1 | azurocidin 1 | -1.63 | 5.59 x 10^-4^ | 35 |
| 1552834_at | B3GNT6 | UDP-GlcNAc:betaGal beta-1,3-N-acetylglucosaminyltransferase 6 (core 3 synthase) | 3.14 | 1.60 x 10^-6^ | 28 |
| 218899_s_at | BAALC | brain and acute leukemia, cytoplasmic | 1.68 | 5.42 x 10^-3^ | 48 |
| 235155_at | BDH2 | 3-hydroxybutyrate dehydrogenase, type 2 | -1.68 | 6.69 x 10^-3^ | 42 |
| 210538_s_at | BIRC3 | baculoviral IAP repeat-containing 3 | -1.69 | 3.16 x 10^-5^ | 100 |
| 229626_at | C12orf68 | chromosome 12 open reading frame 68 | 1.57 | 8.51 x 10^-5^ | 76 |
| 231859_at | C14orf132 /// LOC100132684 | chromosome 14 open reading frame 132 /// hypothetical protein LOC100132684 | -1.51 | 5.36 x 10^-3^ | 70 |
| 219563_at | C14orf139 | chromosome 14 open reading frame 139 | -1.58 | 1.44 x 10^-6^ | 100 |
| 202357_s_at | C2 /// CFB | complement component 2 /// complement factor B | -1.56 | 5.02 x 10^-4^ | 100 |
| 219958_at | C20orf46 | chromosome 20 open reading frame 46 | 1.73 | 2.40 x 10^-6^ | 72 |
| 217767_at | C3 | complement component 3 | -2.75 | 2.63 x 10^-8^ | 100 |
| 28451_s_at | C4A /// C4B | complement component 4A (Rodgers blood group) /// complement component 4B (Chido blood group) | -1.75 | 5.66 x 10^-3^ | 99 |
| 22324_at | C4orf18 | chromosome 4 open reading frame 18 | -1.85 | 2.00 x 10^-3^ | 90 |
| 1555095_at | C6orf123 /// LOC100133056 | chromosome 6 open reading frame 123 /// similar to HGC6.2 | -1.70 | 1.91 x 10^-7^ | 70 |
| 235377_at | C6orf142 | chromosome 6 open reading frame 142 | -1.59 | 8.28 x 10^-3^ | 33 |
| 23056_at | C6orf164 | chromosome 6 open reading frame 164 | 1.56 | 2.85 x 10^-4^ | 87 |
| 1552448_a_at | C8orf12 | chromosome 8 open reading frame 12 | -2.24 | 1.93 x 10^-5^ | 43 |
| 203963_at | CA12 | carbonic anhydrase XII | 1.57 | 1.10 x 10^-3^ | 97 |
| 219928_s_at | CABYR | calcium binding tyrosine-(Y)-phosphorylation regulated | 4.52 | 3.63 x 10^-19^ | 84 |
| 224279_s_at | CABYR | calcium binding tyrosine-(Y)-phosphorylation regulated | 5.12 | 9.25 x 10^-19^ | 64 |
| 231737_at | CACNG4 | calcium channel, voltage-dependent, gamma subunit 4 | 1.92 | 5.16 x 10^-3^ | 30 |
| 62987_r_at | CACNG4 | calcium channel, voltage-dependent, gamma subunit 4 | 1.64 | 1.25 x 10^-3^ | 22 |
| 217561_at | CALCA | calcitonin-related polypeptide alpha | 1.81 | 2.74 x 10^-3^ | 43 |
| 20623_s_at | CALM1 /// CALM2 /// CALM3 | calmodulin 1 (phosphorylase kinase, delta) /// calmodulin 2 (phosphorylase kinase, delta) /// calmodulin 3 (phosphorylase kinase, delta) | -1.51 | 8.41 x 10^-3^ | 82 |
| 209213_at | CBR1 | carbonyl reductase 1 | 2.55 | 1.15 x 10^-8^ | 100 |
| 205379_at | CBR3 | carbonyl reductase 3 | 2.03 | 9.70 x 10^-8^ | 91 |
| 223495_at | CCDC8 | coiled-coil domain containing 8 | -1.75 | 2.26 x 10^-10^ | 100 |
| 1565868_at | CD44 | CD44 molecule (Indian blood group) | 1.59 | 9.84 x 10^-3^ | 50 |
| 215925_s_at | CD72 | CD72 molecule | -1.79 | 3.25 x 10^-4^ | 23 |
| 203213_at | CDC2 | cell division cycle 2, G1 to S and G2 to M | -1.51 | 1.35 x 10^-3^ | 100 |
| 1564674_a_at | CDC20B | cell division cycle 20 homolog B (S. cerevisiae) | -1.54 | 3.74 x 10^-3^ | 57 |
| 240161_s_at | CDC20B | Cell division cycle 20 homolog B (S. cerevisiae), mRNA (cDNA clone IMAGE:526729) | -1.69 | 2.25 x 10^-3^ | 100 |
| 209287_s_at | CDC42EP3 | CDC42 effector protein (Rho GTPase binding) 3 | -1.50 | 3.23 x 10^-5^ | 100 |
| 227850_ x _at | CDC42EP5 | CDC42 effector protein (Rho GTPase binding) 5 | 1.52 | 2.08 x 10^-3^ | 100 |
| 207173_ x _at | CDH11 | cadherin 11, type 2, OB-cadherin (osteoblast) | -1.72 | 1.19 x 10^-4^ | 88 |
| 203440_at | CDH2 | cadherin 2, type 1, N-cadherin (neuronal) | 1.92 | 1.00 x 10^-2^ | 37 |
| 213182_ x _at | CDKN1C | cyclin-dependent kinase inhibitor 1C (p57, Kip2) | -1.58 | 3.72 x 10^-5^ | 71 |
| 213183_s_at | CDKN1C | Cyclin-dependent kinase inhibitor 1C transcript variant 3 (CDKN1C) mRNA, complete cds, alternatively spliced | -1.57 | 2.39 x 10^-3^ | 48 |
| 213348_at | CDKN1C | cyclin-dependent kinase inhibitor 1C (p57, Kip2) | -1.66 | 1.20 x 10^-12^ | 100 |
| 219534_ x _at | CDKN1C | cyclin-dependent kinase inhibitor 1C (p57, Kip2) | -1.61 | 6.59 x 10^-5^ | 75 |
| 201884_at | CEACAM5 | carcinoembryonic antigen-related cell adhesion molecule 5 | 4.59 | 2.96 x 10^-8^ | 100 |
| 203757_s_at | CEACAM6 | carcinoembryonic antigen-related cell adhesion molecule 6 (non-specific cross reacting antigen) | 1.61 | 1.50 x 10^-5^ | 100 |
| 211657_at | CEACAM6 | carcinoembryonic antigen-related cell adhesion molecule 6 (non-specific cross reacting antigen) | 1.51 | 5.81 x 10^-5^ | 100 |
| 218741_at | CENPM | centromere protein M | -1.55 | 2.24 x 10^-5^ | 99 |
| 205382_s_at | CFD | complement factor D (adipsin) | -1.69 | 1.73 x 10^-4^ | 48 |
| 26932_at | CH25H | cholesterol 25-hydroxylase | 1.70 | 3.31 x 10^-4^ | 100 |
| 26869_at | CHAD | chondroadherin | 1.71 | 5.91 x 10^-3^ | 64 |
| 205394_at | CHEK1 | CHK1 checkpoint homolog (S. pombe) | -1.65 | 9.54 x 10^-3^ | 35 |
| 209395_at | CHI3L1 | chitinase 3-like 1 (cartilage glycoprotein-39) | -2.79 | 5.77 x 10^-5^ | 26 |
| 209396_s_at | CHI3L1 | chitinase 3-like 1 (cartilage glycoprotein-39) | -2.34 | 1.14 x 10^-3^ | 24 |
| 221675_s_at | CHPT1 | choline phosphotransferase 1 | -1.64 | 2.32 x 10^-6^ | 100 |
| 20884_at | CKB | creatine kinase, brain | -1.53 | 5.46 x 10^-4^ | 100 |
| 220026_at | CLCA4 | chloride channel regulator 4 | -1.87 | 8.06 x 10^-3^ | 63 |
| 205328_at | CLDN10 | claudin 10 | 1.89 | 2.34 x 10^-8^ | 100 |
| 214598_at | CLDN8 | claudin 8 | -1.80 | 3.74 x 10^-6^ | 100 |
| 219944_at | CLIP4 | CAP-GLY domain containing linker protein family, member 4 | 1.86 | 1.38 x 10^-12^ | 81 |
| 226425_at | CLIP4 | CAP-GLY domain containing linker protein family, member 4 | 2.02 | 5.00 x 10^-10^ | 100 |
| 210769_at | CNGB1 | cyclic nucleotide gated channel beta 1 | 3.12 | 2.26 x 10^-10^ | 46 |
| 201445_at | CNN3 | calponin 3, acidic | -1.80 | 3.22 x 10^-5^ | 100 |
| 231463_at | CNTD1 | cyclin N-terminal domain containing 1 | -1.56 | 1.17 x 10^-5^ | 97 |
| 233202_at | CNTNAP3 | contactin associated protein-like 3 | 1.55 | 3.08 x 10^-3^ | 75 |
| 213622_at | COL9A2 | collagen, type IX, alpha 2 | -1.50 | 1.53 x 10^-6^ | 100 |
| 21949_at | CSGALNACT1 | chondroitin sulfate N-acetylgalactosaminyltransferase 1 | -3.38 | 6.15 x 10^-14^ | 67 |
| 203687_at | CX3CL1 | chemokine (C-X3-C motif) ligand 1 | -2.79 | 1.37 x 10^-6^ | 43 |
| 823_at | CX3CL1 | chemokine (C-X3-C motif) ligand 1 | -2.40 | 3.72 x 10^-12^ | 100 |
| 207850_at | CXCL3 | chemokine (C-X-C motif) ligand 3 | -1.87 | 2.24 x 10^-3^ | 41 |
| 227909_at | CXorf48 /// NCRNA0086 | chromosome X open reading frame 48 /// non-protein coding RNA 86 | -1.59 | 9.18 x 10^-4^ | 49 |
| 222453_at | CYBRD1 | cytochrome b reductase 1 | -1.55 | 9.05 x 10^-4^ | 100 |
| 205749_at | CYP1A1 | cytochrome P450, family 1, subfamily A, polypeptide 1 | 9.73 | 1.23 x 10^-8^ | 25 |
| 202435_s_at | CYP1B1 | cytochrome P450, family 1, subfamily B, polypeptide 1 | 20.83 | 4.21 x 10^-21^ | 61 |
| 202436_s_at | CYP1B1 | cytochrome P450, family 1, subfamily B, polypeptide 1 | 23.44 | 2.42 x 10^-23^ | 68 |
| 202437_s_at | CYP1B1 | cytochrome P450, family 1, subfamily B, polypeptide 1 | 22.73 | 3.08 x 10^-23^ | 71 |
| 26424_at | CYP26A1 | cytochrome P450, family 26, subfamily A, polypeptide 1 | 1.90 | 4.80 x 10^-3^ | 47 |
| 207244_ x _at | CYP2A6 | cytochrome P450, family 2, subfamily A, polypeptide 6 | -1.69 | 4.78 x 10^-6^ | 28 |
| 26153_at | CYP4F11 | cytochrome P450, family 4, subfamily F, polypeptide 11 | 2.69 | 2.41 x 10^-5^ | 86 |
| 26515_at | CYP4F3 | cytochrome P450, family 4, subfamily F, polypeptide 3 | 2.49 | 5.16 x 10^-9^ | 96 |
| 227702_at | CYP4X1 | cytochrome P450, family 4, subfamily X, polypeptide 1 | -1.93 | 1.09 x 10^-11^ | 100 |
| 228738_at | D2HGDH /// LOC100133930 /// LOC257054 | D-2-hydroxyglutarate dehydrogenase /// similar to D-2-hydroxyglutarate dehydrogenase, mitochondrial /// similar to D-2-hydroxyglutarate dehydrogenase | -1.56 | 5.71 x 10^-4^ | 63 |
| 203139_at | DAPK1 | death-associated protein kinase 1 | -1.74 | 2.80 x 10^-6^ | 100 |
| 210397_at | DEFB1 | defensin, beta 1 | 1.72 | 1.85 x 10^-4^ | 92 |
| 218858_at | DEPDC6 | DEP domain containing 6 | -1.56 | 8.02 x 10^-6^ | 99 |
| 218976_at | DNAJC12 | DnaJ (Hsp40) homolog, subfamily C, member 12 | 1.51 | 7.36 x 10^-3^ | 96 |
| 223721_s_at | DNAJC12 | DnaJ (Hsp40) homolog, subfamily C, member 12 | 1.95 | 8.36 x 10^-3^ | 75 |
| 200762_at | DPYSL2 | dihydropyrimidinase-like 2 | -1.62 | 2.93 x 10^-3^ | 100 |
| 214652_at | DRD1 | dopamine receptor D1 | -2.28 | 8.74 x 10^-4^ | 53 |
| 217901_at | DSG2 | desmoglein 2 | -1.61 | 1.14 x 10^-6^ | 100 |
| 205741_s_at | DTNA | dystrobrevin, alpha | 2.10 | 1.09 x 10^-5^ | 72 |
| 21611_s_at | DTNA | dystrobrevin, alpha | 3.17 | 2.71 x 10^-6^ | 25 |
| 22784_at | DTNA | dystrobrevin, alpha | 3.14 | 8.30 x 10^-12^ | 69 |
| 219727_at | DUOX2 | dual oxidase 2 | 1.82 | 6.51 x 10^-3^ | 70 |
| 20141_s_at | DUSP1 | dual specificity phosphatase 1 | -1.56 | 8.58 x 10^-3^ | 100 |
| 209457_at | DUSP5 | dual specificity phosphatase 5 | -2.20 | 1.26 x 10^-3^ | 76 |
| 209343_at | EFHD1 | EF-hand domain family, member D1 | -1.59 | 8.97 x 10^-6^ | 52 |
| 202668_at | EFNB2 | ephrin-B2 | -1.59 | 3.22 x 10^-6^ | 100 |
| 26254_at | EGF | epidermal growth factor (beta-urogastrone) | 3.19 | 8.25 x 10^-10^ | 57 |
| 219454_at | EGFL6 | EGF-like-domain, multiple 6 | -1.81 | 6.47 x 10^-7^ | 100 |
| 231292_at | EID3 | EP300 interacting inhibitor of differentiation 3 | -1.66 | 1.09 x 10^-4^ | 84 |
| 1559072_a_at | ELFN2 | extracellular leucine-rich repeat and fibronectin type III domain containing 2 | 3.17 | 4.28 x 10^-11^ | 74 |
| 1560713_a_at | ELFN2 | extracellular leucine-rich repeat and fibronectin type III domain containing 2 | 2.42 | 1.41 x 10^-4^ | 22 |
| 231930_at | ELMOD1 | ELMO/CED-12 domain containing 1 | 3.45 | 4.17 x 10^-9^ | 90 |
| 201718_s_at | EPB41L2 | erythrocyte membrane protein band 4.1-like 2 | -2.09 | 1.14 x 10^-7^ | 50 |
| 201719_s_at | EPB41L2 | erythrocyte membrane protein band 4.1-like 2 | -2.21 | 3.95 x 10^-12^ | 88 |
| 26710_s_at | EPB41L3 | erythrocyte membrane protein band 4.1-like 3 | -1.56 | 3.73 x 10^-3^ | 83 |
| 228948_at | EPHA4 | EPH receptor A4 | -1.63 | 9.82 x 10^-3^ | 56 |
| 23425_at | EPHB1 | EPH receptor B1 | 2.39 | 7.37 x 10^-5^ | 38 |
| 227450_at | ERP27 | endoplasmic reticulum protein 27 kDa | -2.4 | 4.60 x 10^-6^ | 100 |
| 219432_at | EVC | Ellis van Creveld syndrome | -1.69 | 5.13 x 10^-4^ | 47 |
| 1566123_at | FABP6 | fatty acid binding protein 6, ileal | -1.52 | 1.84 x 10^-4^ | 70 |
| 209074_s_at | FAM107A | family with sequence similarity 107, member A | -1.76 | 8.89 x 10^-4^ | 84 |
| 214945_at | FAM153A /// FAM153B /// FAM153C | family with sequence similarity 153, member A /// family with sequence similarity 153, member B /// family with sequence similarity 153, member C | 1.81 | 7.27 x 10^-5^ | 98 |
| 1555485_s_at | FAM153B | family with sequence similarity 153, member B | 1.92 | 1.97 x 10^-3^ | 28 |
| 202771_at | FAM38A | family with sequence similarity 38, member A | -1.88 | 1.07 x 10^-7^ | 91 |
| 219602_s_at | FAM38B | family with sequence similarity 38, member B | -1.64 | 2.10 x 10^-4^ | 74 |
| 22298_at | FAM38B | family with sequence similarity 38, member B | -2.22 | 3.33 x 10^-5^ | 65 |
| 227654_at | FAM65C | family with sequence similarity 65, member C | -2.60 | 2.09 x 10^-7^ | 41 |
| 225834_at | FAM72A /// FAM72B /// GCUD2 | family with sequence similarity 72, member A /// family with sequence similarity 72, member B /// gastric cancer up-regulated-2 | -1.50 | 5.96 x 10^-4^ | 87 |
| 202766_s_at | FBN1 | fibrillin 1 | -2.4 | 8.83 x 10^-8^ | 70 |
| 221812_at | FBXO42 | F-box protein 42 | -1.68 | 9.29 x 10^-5^ | 38 |
| 223870_at | FBXW10 | F-box and WD repeat domain containing 10 | -1.57 | 4.48 x 10^-7^ | 99 |
| 203639_s_at | FGFR2 | fibroblast growth factor receptor 2 | -1.50 | 6.69 x 10^-3^ | 97 |
| 211401_s_at | FGFR2 | fibroblast growth factor receptor 2 | -1.73 | 1.26 x 10^-3^ | 68 |
| 24379_s_at | FGFR3 | fibroblast growth factor receptor 3 | -1.57 | 1.77 x 10^-4^ | 99 |
| 218980_at | FHOD3 | formin homology 2 domain containing 3 | -1.53 | 8.70 x 10^-4^ | 100 |
| 1558234_at | FLJ36644 | hypothetical gene supported by AK093963 | -1.85 | 3.40 x 10^-5^ | 73 |
| 23419_at | FLJ37644 /// LOC727973 | hypothetical gene supported by AK094963 /// hypothetical protein LOC727973 | -1.66 | 8.54 x 10^-5^ | 80 |
| 227925_at | FLJ39051 | CDNA FLJ39051 fis, clone NT2RP7011452 | 3.68 | 5.31 x 10^-10^ | 38 |
| 230999_at | FLJ39051 | CDNA FLJ39051 fis, clone NT2RP7011452 | 1.85 | 2.16 x 10^-6^ | 54 |
| 156940_s_at | FLJ40330 | hypothetical LOC645784 | -2.22 | 7.09 x 10^-4^ | 49 |
| 228268_at | FMO2 | flavin containing monooxygenase 2 (non-functional) | -1.68 | 3.58 x 10^-6^ | 100 |
| 202709_at | FMOD | fibromodulin | 1.71 | 1.49 x 10^-6^ | 100 |
| 225922_at | FNIP2 | folliculin interacting protein 2 | -1.53 | 2.93 x 10^-7^ | 100 |
| 24437_s_at | FOLR1 | folate receptor 1 (adult) | -1.64 | 9.56 x 10^-5^ | 100 |
| 210103_s_at | FOXA2 | forkhead box A2 | -1.68 | 3.35 x 10^-3^ | 33 |
| 40284_at | FOXA2 | forkhead box A2 | -1.59 | 1.19 x 10^-3^ | 95 |
| 1560031_at | FRMD4A | FERM domain containing 4A | -1.68 | 1.80 x 10^-4^ | 84 |
| 225163_at | FRMD4A | FERM domain containing 4A | -1.72 | 1.67 x 10^-7^ | 95 |
| 225167_at | FRMD4A | FERM domain containing 4A | -1.51 | 4.83 x 10^-7^ | 98 |
| 225168_at | FRMD4A | FERM domain containing 4A | -1.50 | 4.28 x 10^-5^ | 86 |
| 217897_at | FXYD6 | FXYD domain containing ion transport regulator 6 | -1.53 | 1.41 x 10^-3^ | 41 |
| 224325_at | FZD8 | frizzled homolog 8 (Drosophila) | -1.54 | 2.25 x 10^-5^ | 97 |
| 227405_s_at | FZD8 | frizzled homolog 8 (Drosophila) | -1.58 | 2.43 x 10^-8^ | 98 |
| 213524_s_at | G0S2 | G0/G1switch 2 | -1.62 | 1.77 x 10^-4^ | 98 |
| 202275_at | G6PD | glucose-6-phosphate dehydrogenase | 1.93 | 2.49 x 10^-7^ | 55 |
| 205890_s_at | GABBR1 /// UBD | gamma-aminobutyric acid (GABA) B receptor, 1 /// ubiquitin D | -1.75 | 2.85 x 10^-4^ | 98 |
| 205278_at | GAD1 | glutamate decarboxylase 1 (brain, 67kDa) | 6.27 | 1.62 x 10^-10^ | 59 |
| 26669_at | GAD1 | glutamate decarboxylase 1 (brain, 67kDa) | 1.99 | 8.00 x 10^-3^ | 25 |
| 207574_s_at | GADD45B | growth arrest and DNA-damage-inducible, beta | -1.61 | 4.04 x 10^-6^ | 99 |
| 20934_ x _at | GADD45B | growth arrest and DNA-damage-inducible, beta | -1.55 | 2.37 x 10^-4^ | 62 |
| 209305_s_at | GADD45B | growth arrest and DNA-damage-inducible, beta | -1.57 | 8.49 x 10^-5^ | 94 |
| 229555_at | GALNT5 | UDP-N-acetyl-alpha-D-galactosamine:polypeptide N-acetylgalactosaminyltransferase 5 (GalNAc-T5) | 1.50 | 5.41 x 10^-3^ | 61 |
| 237183_at | GALNT5 | CDNA FLJ75131 complete cds, highly similar to Homo sapiens UDP-N-acetyl-alpha-D-galactosamine:polypeptide N-acetylgalactosaminyltransferase 5 (GalNAc-T5) (GALNT5), mRNA | 1.62 | 9.12 x 10^-7^ | 99 |
| 219956_at | GALNT6 | UDP-N-acetyl-alpha-D-galactosamine:polypeptide N-acetylgalactosaminyltransferase 6 (GalNAc-T6) | 1.67 | 6.61 x 10^-5^ | 100 |
| 1555330_at | GCLC | glutamate-cysteine ligase, catalytic subunit | 1.79 | 1.86 x 10^-5^ | 92 |
| 202922_at | GCLC | glutamate-cysteine ligase, catalytic subunit | 1.52 | 3.01 x 10^-6^ | 100 |
| 202923_s_at | GCLC | glutamate-cysteine ligase, catalytic subunit | 1.62 | 2.13 x 10^-9^ | 100 |
| 224209_s_at | GDA | guanine deaminase | -1.93 | 2.67 x 10^-6^ | 97 |
| 24472_at | GEM | GTP binding protein overexpressed in skeletal muscle | -1.75 | 3.84 x 10^-3^ | 90 |
| 1569886_a_at | GLB1L3 | galactosidase, beta 1-like 3 | 1.89 | 1.21 x 10^-3^ | 49 |
| 227376_at | GLI3 | GLI-Kruppel family member GLI3 | -1.66 | 2.01 x 10^-10^ | 100 |
| 205279_s_at | GLRB | glycine receptor, beta | -1.59 | 4.36 x 10^-6^ | 99 |
| 205280_at | GLRB | glycine receptor, beta | -1.72 | 2.08 x 10^-4^ | 93 |
| 237690_at | GPR115 | G protein-coupled receptor 115 | -2.01 | 1.23 x 10^-3^ | 34 |
| 21473_s_at | GPR125 | G protein-coupled receptor 125 | -1.53 | 1.34 x 10^-6^ | 100 |
| 202831_at | GPX2 | glutathione peroxidase 2 (gastrointestinal) | 5.56 | 4.54 x 10^-24^ | 99 |
| 239595_at | GPX2 | glutathione peroxidase 2 (gastrointestinal) | 3.39 | 6.34 x 10^-14^ | 74 |
| 214217_at | GRM5 | glutamate receptor, metabotropic 5 | -2.8 | 1.37 x 10^-5^ | 89 |
| 225609_at | GSR | glutathione reductase | 1.59 | 2.99 x 10^-7^ | 100 |
| 224646_ x _at | H19 | H19, imprinted maternally expressed transcript (non-protein coding) | 5.25 | 4.86 x 10^-7^ | 84 |
| 224997_ x _at | H19 | H19, imprinted maternally expressed transcript (non-protein coding) | 2.80 | 3.59 x 10^-9^ | 94 |
| 211998_at | H3F3A /// H3F3B /// LOC440926 | H3 histone, family 3A /// H3 histone, family 3B (H3.3B) /// H3 histone, family 3A pseudogene | -1.56 | 1.24 x 10^-7^ | 100 |
| 242601_at | HEPACAM2 | HEPACAM family member 2 | 1.70 | 2.86 x 10^-5^ | 100 |
| 226446_at | HES6 | hairy and enhancer of split 6 (Drosophila) | -2.72 | 1.62 x 10^-6^ | 43 |
| 219743_at | HEY2 | hairy/enhancer-of-split related with YRPW motif 2 | -1.54 | 3.11 x 10^-6^ | 100 |
| 205221_at | HGD /// LOC100132552 /// LOC727722 | homogentisate 1,2-dioxygenase (homogentisate oxidase) /// similar to homogentisate 1,2-dioxygenase /// homogentisate 1,2-dioxygenase like | 2.99 | 1.87 x 10^-7^ | 90 |
| 214307_at | HGD /// LOC100132552 /// LOC727722 | homogentisate 1,2-dioxygenase (homogentisate oxidase) /// similar to homogentisate 1,2-dioxygenase /// homogentisate 1,2-dioxygenase like | 2.15 | 3.31 x 10^-4^ | 27 |
| 21438_s_at | HGD /// LOC100132552 /// LOC727722 | homogentisate 1,2-dioxygenase (homogentisate oxidase) /// similar to homogentisate 1,2-dioxygenase /// homogentisate 1,2-dioxygenase like | 2.02 | 1.36 x 10^-9^ | 96 |
| 205425_at | HIP1 | huntingtin interacting protein 1 | -1.55 | 2.33 x 10^-5^ | 77 |
| 226364_at | HIP1 | Huntingtin interacting protein (HIP1) | -1.52 | 1.82 x 10^-5^ | 93 |
| 235122_at | HIVEP3 | human immunodeficiency virus type I enhancer binding protein 3 | -1.68 | 2.68 x 10^-3^ | 55 |
| 215536_at | HLA-DQB2 | major histocompatibility complex, class II, DQ beta 2 | -1.63 | 9.69 x 10^-4^ | 50 |
| 24753_s_at | HLF | hepatic leukemia factor | -1.52 | 7.62 x 10^-5^ | 94 |
| 24754_at | HLF | hepatic leukemia factor | -1.53 | 7.81 x 10^-5^ | 63 |
| 24111_at | HNMT | histamine N-methyltransferase | -1.72 | 1.14 x 10^-3^ | 26 |
| 228772_at | HNMT | histamine N-methyltransferase | -1.55 | 5.79 x 10^-5^ | 100 |
| 211597_s_at | HOPX | HOP homeobox | -1.60 | 2.60 x 10^-3^ | 60 |
| 214639_s_at | HOXA1 | homeobox A1 | 2.92 | 8.24 x 10^-5^ | 34 |
| 26194_at | HOXC4 | homeobox C4 | -1.65 | 3.17 x 10^-4^ | 62 |
| 26864_s_at | HRK | harakiri, BCL2 interacting protein (contains only BH3 domain) | 1.65 | 1.04 x 10^-3^ | 92 |
| 1552767_a_at | HS6ST2 | heparan sulfate 6-O-sulfotransferase 2 | 1.55 | 9.38 x 10^-4^ | 100 |
| 230030_at | HS6ST2 | heparan sulfate 6-O-sulfotransferase 2 | 1.54 | 1.35 x 10^-3^ | 100 |
| 24818_at | HSD17B2 | hydroxysteroid (17-beta) dehydrogenase 2 | -2.15 | 3.63 x 10^-3^ | 55 |
| 211538_s_at | HSPA2 | heat shock 70kDa protein 2 | -1.51 | 1.30 x 10^-4^ | 100 |
| 209448_at | HTATIP2 | HIV-1 Tat interactive protein 2, 30kDa | 1.75 | 9.62 x 10^-9^ | 100 |
| 210253_at | HTATIP2 | HIV-1 Tat interactive protein 2, 30kDa | 1.61 | 8.33 x 10^-10^ | 100 |
| 21666_at | IDS | iduronate 2-sulfatase | 1.64 | 2.98 x 10^-5^ | 95 |
| 211782_at | IDS | iduronate 2-sulfatase | 1.80 | 3.36 x 10^-4^ | 67 |
| 1553857_at | IGSF22 | immunoglobulin superfamily, member 22 | -1.56 | 4.53 x 10^-4^ | 68 |
| 22262_at | IL27RA | interleukin 27 receptor, alpha | -1.54 | 3.16 x 10^-3^ | 56 |
| 203233_at | IL4R | interleukin 4 receptor | -1.52 | 1.51 x 10^-8^ | 86 |
| 224571_at | IRF2BP2 | interferon regulatory factor 2 binding protein 2 | -1.56 | 1.17 x 10^-7^ | 100 |
| 209185_s_at | IRS2 | insulin receptor substrate 2 | -1.70 | 3.26 x 10^-5^ | 100 |
| 205032_at | ITGA2 | integrin, alpha 2 (CD49B, alpha 2 subunit of VLA-2 receptor) | -1.75 | 5.53 x 10^-5^ | 99 |
| 227314_at | ITGA2 | integrin, alpha 2 (CD49B, alpha 2 subunit of VLA-2 receptor) | -1.62 | 1.98 x 10^-7^ | 100 |
| 227297_at | ITGA9 | integrin, alpha 9 | -1.62 | 1.01 x 10^-4^ | 91 |
| 223597_at | ITLN1 | intelectin 1 (galactofuranose binding) | -4.81 | 2.71 x 10^-6^ | 49 |
| 202746_at | ITM2A | integral membrane protein 2A | -2.17 | 7.97 x 10^-7^ | 99 |
| 202747_s_at | ITM2A | integral membrane protein 2A | -1.79 | 4.93 x 10^-5^ | 88 |
| 233076_at | JAKMIP3 | janus kinase and microtubule interacting protein 3 | 3.62 | 6.37 x 10^-8^ | 30 |
| 28479_at | KCNA1 | potassium voltage-gated channel, shaker-related subfamily, member 1 (episodic ataxia with myokymia) | -1.68 | 2.65 x 10^-3^ | 29 |
| 23849_at | KCNA1 | potassium voltage-gated channel, shaker-related subfamily, member 1 (episodic ataxia with myokymia) | -1.88 | 1.77 x 10^-4^ | 96 |
| 21106_s_at | KCNB1 | potassium voltage-gated channel, Shab-related subfamily, member 1 | -1.73 | 1.04 x 10^-8^ | 87 |
| 235467_s_at | KCNC4 | potassium voltage-gated channel, Shaw-related subfamily, member 4 | -1.62 | 1.74 x 10^-5^ | 27 |
| 221584_s_at | KCNMA1 | potassium large conductance calcium-activated channel, subfamily M, alpha member 1 | -1.68 | 8.65 x 10^-4^ | 53 |
| 223823_at | KCNMB2 | potassium large conductance calcium-activated channel, subfamily M, beta member 2 | -1.50 | 4.20 x 10^-4^ | 100 |
| 228325_at | KIAA0146 | Clone C4E 5.1 (CAC)n/(GTG)n repeat-containing mRNA | -1.57 | 2.83 x 10^-5^ | 95 |
| 227502_at | KIAA1147 | KIAA1147 | 1.52 | 4.96 x 10^-6^ | 99 |
| 1560397_s_at | KLHL6 | kelch-like 6 (Drosophila) | 1.68 | 4.42 x 10^-3^ | 86 |
| 213240_s_at | KRT4 | keratin 4 | -1.75 | 5.41 x 10^-4^ | 94 |
| 209270_at | LAMB3 | laminin, beta 3 | -1.62 | 1.13 x 10^-5^ | 94 |
| 209894_at | LEPR | leptin receptor | -1.72 | 1.83 x 10^-5^ | 91 |
| 211355_ x _at | LEPR | leptin receptor | -1.62 | 8.17 x 10^-3^ | 25 |
| 219884_at | LHX6 | LIM homeobox 6 | 1.69 | 3.70 x 10^-4^ | 52 |
| 203276_at | LMNB1 | lamin B1 | -1.59 | 1.26 x 10^-9^ | 98 |
| 236656_s_at | LOC10013056 | hypothetical protein LOC10013056 | -1.56 | 2.10 x 10^-6^ | 70 |
| 215275_at | LOC100133233 /// TRAF3IP3 | hypothetical protein LOC100133233 /// TRAF3 interacting protein 3 | -1.66 | 7.48 x 10^-3^ | 21 |
| 201463_s_at | LOC100133665 /// TALDO1 | similar to transaldolase /// transaldolase 1 | 1.65 | 5.54 x 10^-10^ | 100 |
| 229930_at | LOC100134361 | similar to hCG1811002 | -1.52 | 2.82 x 10^-5^ | 80 |
| 1556768_at | LOC10014464 | hypothetical LOC10014464 | 1.96 | 6.83 x 10^-9^ | 89 |
| 226809_at | LOC100216479 | hypothetical LOC100216479 | -1.70 | 3.82 x 10^-5^ | 93 |
| 242098_at | LOC202451 | hypothetical protein LOC202451 | -1.50 | 2.71 x 10^-3^ | 99 |
| 1557207_s_at | LOC283177 | hypothetical protein LOC283177 | -1.67 | 1.41 x 10^-4^ | 97 |
| 241370_at | LOC286052 | hypothetical protein LOC286052 | -1.53 | 1.68 x 10^-6^ | 100 |
| 241418_at | LOC344887 | similar to hCG241270 | 3.65 | 1.02 x 10^-8^ | 63 |
| 1558423_at | LOC349114 | Homo sapiens, clone IMAGE:4385460, mRNA | 1.68 | 7.03 x 10^-4^ | 44 |
| 1558722_at | LOC441383 /// ZNF252 | hypothetical gene supported by AF86559; BC65734 /// zinc finger protein 252 | -1.54 | 5.97 x 10^-3^ | 72 |
| 225355_at | LOC54492 | neuralized-2 | -1.61 | 6.62 x 10^-6^ | 100 |
| 229566_at | LOC645638 | similar to WDNM1-like protein | -1.60 | 4.12 x 10^-5^ | 76 |
| 205710_at | LRP2 | low density lipoprotein-related protein 2 | -1.50 | 5.46 x 10^-3^ | 78 |
| 23863_at | LRP2 | low density lipoprotein-related protein 2 | -2.10 | 2.44 x 10^-5^ | 68 |
| 22622_at | LRRC31 | leucine rich repeat containing 31 | 2.27 | 5.99 x 10^-6^ | 84 |
| 226884_at | LRRN1 | leucine rich repeat neuronal 1 | -1.52 | 1.31 x 10^-3^ | 100 |
| 202729_s_at | LTBP1 | latent transforming growth factor beta binding protein 1 | -1.68 | 7.31 x 10^-9^ | 94 |
| 202018_s_at | LTF | lactotransferrin | -3.42 | 1.28 x 10^-9^ | 91 |
| 221261_ x _at | MAGED4 /// MAGED4B | melanoma antigen family D, 4 /// melanoma antigen family D, 4B | 1.82 | 1.88 x 10^-4^ | 54 |
| 209737_at | MAGI2 | membrane associated guanylate kinase, WW and PDZ domain containing 2 | -1.52 | 1.09 x 10^-8^ | 100 |
| 224559_at | MALAT1 | metastasis associated lung adenocarcinoma transcript 1 (non-protein coding) | 1.76 | 6.84 x 10^-3^ | 100 |
| 209373_at | MALL | mal, T-cell differentiation protein-like | -1.64 | 1.48 x 10^-5^ | 85 |
| 2441_at | MAOB | monoamine oxidase B | -2.62 | 1.90 x 10^-12^ | 100 |
| 22684_at | MAP1B | microtubule-associated protein 1B | 1.70 | 2.54 x 10^-7^ | 100 |
| 214786_at | MAP3K1 | mitogen-activated protein kinase kinase kinase 1 | -1.54 | 3.18 x 10^-3^ | 73 |
| 20644_at | MARCKSL1 | MARCKS-like 1 | -1.56 | 2.05 x 10^-3^ | 98 |
| 24179_at | MB | myoglobin | -1.75 | 2.08 x 10^-6^ | 100 |
| 230110_at | MCOLN2 | mucolipin 2 | -1.56 | 4.57 x 10^-4^ | 73 |
| 1557292_a_at | MCOLN3 | mucolipin 3 | -1.54 | 6.67 x 10^-3^ | 52 |
| 20361_s_at | MDC1 | mediator of DNA damage checkpoint 1 | -1.72 | 6.61 x 10^-3^ | 40 |
| 24058_at | ME1 | malic enzyme 1, NADP(+)-dependent, cytosolic | 3.59 | 1.92 x 10^-16^ | 99 |
| 24059_s_at | ME1 | malic enzyme 1, NADP(+)-dependent, cytosolic | 3.62 | 1.24 x 10^-22^ | 100 |
| 209199_s_at | MEF2C | myocyte enhancer factor 2C | -1.63 | 2.19 x 10^-5^ | 61 |
| 225316_at | MFSD2 | major facilitator superfamily domain containing 2 | 1.64 | 3.56 x 10^-3^ | 60 |
| 155583_at | MGC34774 | ribosomal protein L13A pseudogene | -1.59 | 8.48 x 10^-3^ | 41 |
| 244246_at | MIPOL1 | mirror-image polydactyly 1 | 1.55 | 7.40 x 10^-3^ | 92 |
| 24259_at | MMP7 | matrix metallopeptidase 7 (matrilysin, uterine) | -1.73 | 4.17 x 10^-3^ | 29 |
| 205413_at | MPPED2 | metallophosphoesterase domain containing 2 | 1.75 | 2.38 x 10^-3^ | 63 |
| 207430_s_at | MSMB | microseminoprotein, beta- | 1.93 | 2.58 x 10^-6^ | 100 |
| 210297_s_at | MSMB | microseminoprotein, beta- | 1.80 | 4.42 x 10^-6^ | 100 |
| 225782_at | MSRB3 | methionine sulfoxide reductase B3 | -2.31 | 3.88 x 10^-6^ | 87 |
| 225790_at | MSRB3 | methionine sulfoxide reductase B3 | -2.20 | 1.86 x 10^-4^ | 42 |
| 212859_ x _at | MT1E | metallothionein 1E | -1.75 | 5.05 x 10^-8^ | 100 |
| 213629_ x _at | MT1F | metallothionein 1F | -1.85 | 9.63 x 10^-10^ | 100 |
| 217165_ x _at | MT1F | metallothionein 1F | -1.84 | 7.89 x 10^-9^ | 100 |
| 24745_ x _at | MT1G | metallothionein 1G | -1.64 | 1.18 x 10^-10^ | 96 |
| 26461_ x _at | MT1H | metallothionein 1H | -1.66 | 2.65 x 10^-8^ | 71 |
| 211456_ x _at | MT1P2 | metallothionein 1 pseudogene 2 | -1.59 | 2.35 x 10^-5^ | 100 |
| 24326_ x _at | MT1X | metallothionein 1X | -1.55 | 1.87 x 10^-5^ | 100 |
| 28581_ x _at | MT1X | metallothionein 1X | -1.51 | 9.62 x 10^-5^ | 100 |
| 212185_ x _at | MT2A | metallothionein 2A | -1.59 | 8.77 x 10^-6^ | 100 |
| 205970_at | MT3 | metallothionein 3 | -1.70 | 3.08 x 10^-5^ | 27 |
| 239846_at | MTHFD1 | CDNA FLJ38195 fis, clone FCBBF1000314 | -1.60 | 9.09 x 10^-3^ | 30 |
| 219786_at | MTL5 | metallothionein-like 5, testis-specific (tesmin) | -1.56 | 1.19 x 10^-5^ | 56 |
| 214303_ x _at | MUC5AC | mucin 5AC, oligomeric mucus/gel-forming | 3.17 | 1.24 x 10^-7^ | 100 |
| 214385_s_at | MUC5AC | mucin 5AC, oligomeric mucus/gel-forming | 2.80 | 1.34 x 10^-7^ | 100 |
| 217182_at | MUC5AC | mucin 5AC, oligomeric mucus/gel-forming | 3.13 | 2.25 x 10^-5^ | 77 |
| 217187_at | MUC5AC | mucin 5AC, oligomeric mucus/gel-forming | 3.05 | 8.95 x 10^-6^ | 95 |
| 1553602_at | MUCL1 | mucin-like 1 | 6.34 | 4.79 x 10^-23^ | 100 |
| 202555_s_at | MYLK | myosin light chain kinase | -1.54 | 8.84 x 10^-6^ | 97 |
| 224823_at | MYLK | myosin light chain kinase | -1.85 | 8.82 x 10^-10^ | 100 |
| 224771_at | NAV1 | neuron navigator 1 | 2.10 | 9.12 x 10^-6^ | 37 |
| 224772_at | NAV1 | neuron navigator 1 | 1.62 | 5.16 x 10^-9^ | 100 |
| 224774_s_at | NAV1 | neuron navigator 1 | 1.65 | 1.63 x 10^-3^ | 70 |
| 1552658_a_at | NAV3 | neuron navigator 3 | -2.14 | 1.82 x 10^-5^ | 55 |
| 24823_at | NAV3 | neuron navigator 3 | -2.54 | 1.39 x 10^-5^ | 24 |
| 225344_at | NCOA7 | nuclear receptor coactivator 7 | -1.51 | 9.15 x 10^-5^ | 100 |
| 22762_at | NCRNA0084 | non-protein coding RNA 84 | 1.66 | 4.24 x 10^-3^ | 99 |
| 231491_at | NCRNA00113 | non-protein coding RNA 113 | -1.62 | 4.24 x 10^-3^ | 67 |
| 214799_at | NFASC | neurofascin homolog (chicken) | 1.91 | 5.34 x 10^-3^ | 31 |
| 236471_at | NFE2L3 | nuclear factor (erythroid-derived 2)-like 3 | -1.82 | 7.66 x 10^-4^ | 59 |
| 201502_s_at | NFKBIA | nuclear factor of kappa light polypeptide gene enhancer in B-cells inhibitor, alpha | -1.51 | 8.65 x 10^-5^ | 100 |
| 1558738_at | NOL3 | CDNA FLJ58768 complete cds, highly similar to Homo sapiens nucleolar protein 3 (apoptosis repressor with CARD domain) (NOL3), mRNA | 1.74 | 2.10 x 10^-8^ | 86 |
| 205794_s_at | NOVA1 | neuro-oncological ventral antigen 1 | 2.17 | 4.93 x 10^-5^ | 64 |
| 220316_at | NPAS3 | neuronal PAS domain protein 3 | -1.69 | 4.83 x 10^-7^ | 78 |
| 229281_at | NPAS3 | neuronal PAS domain protein 3 | -2.26 | 2.31 x 10^-10^ | 93 |
| 23412_at | NPAS3 | neuronal PAS domain protein 3 | -2.14 | 5.54 x 10^-10^ | 80 |
| 201467_s_at | NQO1 | NAD(P)H dehydrogenase, quinone 1 | 2.93 | 2.35 x 10^-16^ | 100 |
| 201468_s_at | NQO1 | NAD(P)H dehydrogenase, quinone 1 | 3.21 | 6.77 x 10^-18^ | 100 |
| 210519_s_at | NQO1 | NAD(P)H dehydrogenase, quinone 1 | 2.50 | 5.01 x 10^-16^ | 100 |
| 26645_s_at | NR0B1 | nuclear receptor subfamily 0, group B, member 1 | 2.59 | 2.49 x 10^-4^ | 30 |
| 209959_at | NR4A3 | nuclear receptor subfamily 4, group A, member 3 | 2.09 | 8.04 x 10^-3^ | 46 |
| 228547_at | NRXN1 | neurexin 1 | -1.69 | 2.16 x 10^-4^ | 72 |
| 1553995_a_at | NT5E | 5'-nucleotidase, ecto (CD73) | -2.03 | 1.45 x 10^-6^ | 55 |
| 203939_at | NT5E | 5'-nucleotidase, ecto (CD73) | -2.26 | 9.52 x 10^-13^ | 100 |
| 219489_s_at | NXN | nucleoredoxin | -1.81 | 1.90 x 10^-8^ | 100 |
| 226649_at | PANK1 | pantothenate kinase 1 | -1.95 | 1.59 x 10^-12^ | 98 |
| 23967_s_at | PANX2 | pannexin 2 | 1.55 | 3.19 x 10^-3^ | 83 |
| 203058_s_at | PAPSS2 | 3'-phosphoadenosine 5'-phosphosulfate synthase 2 | -1.52 | 4.38 x 10^-4^ | 96 |
| 203059_s_at | PAPSS2 | 3'-phosphoadenosine 5'-phosphosulfate synthase 2 | -1.50 | 2.81 x 10^-3^ | 43 |
| 20360_s_at | PAPSS2 | 3'-phosphoadenosine 5'-phosphosulfate synthase 2 | -1.82 | 9.73 x 10^-6^ | 98 |
| 214401_at | PAX1 | paired box 1 | -1.79 | 2.81 x 10^-5^ | 42 |
| 227289_at | PCDH17 | protocadherin 17 | -1.65 | 8.86 x 10^-4^ | 95 |
| 228863_at | PCDH17 | protocadherin 17 | -1.82 | 1.25 x 10^-3^ | 93 |
| 232054_at | PCDH20 | protocadherin 20 | -1.62 | 5.60 x 10^-5^ | 98 |
| 218952_at | PCSK1N | proprotein convertase subtilisin/kexin type 1 inhibitor | 1.76 | 1.86 x 10^-3^ | 48 |
| 207414_s_at | PCSK6 | proprotein convertase subtilisin/kexin type 6 | -2.01 | 9.52 x 10^-13^ | 78 |
| 242662_at | PCSK6 | PACE4A-II | -1.77 | 4.07 x 10^-5^ | 63 |
| 227759_at | PCSK9 | proprotein convertase subtilisin/kexin type 9 | -1.57 | 7.53 x 10^-3^ | 59 |
| 205960_at | PDK4 | pyruvate dehydrogenase kinase, isozyme 4 | -1.57 | 6.75 x 10^-5^ | 95 |
| 225207_at | PDK4 | pyruvate dehydrogenase kinase, isozyme 4 | -1.63 | 3.03 x 10^-10^ | 100 |
| 211564_s_at | PDLIM4 | PDZ and LIM domain 4 | -1.50 | 4.16 x 10^-3^ | 99 |
| 1553589_a_at | PDZK1IP1 | PDZK1 interacting protein 1 | -1.66 | 1.85 x 10^-4^ | 100 |
| 219630_at | PDZK1IP1 | PDZK1 interacting protein 1 | -1.62 | 4.79 x 10^-5^ | 97 |
| 212092_at | PEG10 | paternally expressed 10 | -1.60 | 1.41 x 10^-3^ | 51 |
| 212094_at | PEG10 | paternally expressed 10 | -1.56 | 4.63 x 10^-4^ | 97 |
| 1558164_s_at | PEX13 | peroxisomal biogenesis factor 13 | -1.61 | 8.11 x 10^-4^ | 60 |
| 2464_at | PFTK1 | PFTAIRE protein kinase 1 | -1.52 | 2.08 x 10^-7^ | 100 |
| 201118_at | PGD | phosphogluconate dehydrogenase | 1.55 | 4.72 x 10^-5^ | 100 |
| 21617_at | PHEX | phosphate regulating endopeptidase homolog, X-linked | 2.62 | 6.29 x 10^-10^ | 47 |
| 217996_at | PHLDA1 | pleckstrin homology-like domain, family A, member 1 | 1.51 | 2.97 x 10^-4^ | 97 |
| 217997_at | PHLDA1 | pleckstrin homology-like domain, family A, member 1 | 1.73 | 1.85 x 10^-5^ | 71 |
| 203691_at | PI3 | peptidase inhibitor 3, skin-derived | -2.22 | 2.51 x 10^-4^ | 70 |
| 41469_at | PI3 | peptidase inhibitor 3, skin-derived | -2.02 | 2.35 x 10^-5^ | 92 |
| 205632_s_at | PIP5K1B | phosphatidylinositol-4-phosphate 5-kinase, type I, beta | -1.54 | 7.54 x 10^-8^ | 100 |
| 207469_s_at | PIR | pirin (iron-binding nuclear protein) | 2.74 | 1.21 x 10^-16^ | 100 |
| 223551_at | PKIB | protein kinase (cAMP-dependent, catalytic) inhibitor beta | -1.52 | 1.76 x 10^-4^ | 100 |
| 231120_ x _at | PKIB | protein kinase (cAMP-dependent, catalytic) inhibitor beta | -1.59 | 1.81 x 10^-4^ | 100 |
| 207222_at | PLA2G10 | phospholipase A2, group X | 1.51 | 3.63 x 10^-4^ | 100 |
| 201860_s_at | PLAT | plasminogen activator, tissue | 1.57 | 3.90 x 10^-3^ | 66 |
| 236255_at | PLEKHG4B | pleckstrin homology domain containing, family G (with RhoGef domain) member 4B | -1.51 | 1.23 x 10^-4^ | 80 |
| 209977_at | PLG | plasminogen | 2.45 | 6.88 x 10^-3^ | 26 |
| 240033_at | PLG | plasminogen | 3.18 | 1.28 x 10^-4^ | 28 |
| 201939_at | PLK2 | polo-like kinase 2 (Drosophila) | -2.42 | 1.71 x 10^-11^ | 98 |
| 24887_s_at | PLK4 | polo-like kinase 4 (Drosophila) | -1.50 | 2.52 x 10^-3^ | 96 |
| 209034_at | PNRC1 | proline-rich nuclear receptor coactivator 1 | -1.51 | 3.01 x 10^-5^ | 100 |
| 1569675_at | POU2AF1 | POU class 2 associating factor 1, mRNA (cDNA clone MGC:45211 IMAGE:5554134) | -1.81 | 6.3 x 10^-8^ | 100 |
| 214858_at | PP14571 | similar to hCG1777210 | 1.70 | 1.12 x 10^-4^ | 95 |
| 209355_s_at | PPAP2B | phosphatidic acid phosphatase type 2B | -1.59 | 3.00 x 10^-5^ | 99 |
| 212750_at | PPP1R16B | protein phosphatase 1, regulatory (inhibitor) subunit 16B | -3.31 | 1.9 x 10^-10^ | 28 |
| 41577_at | PPP1R16B | protein phosphatase 1, regulatory (inhibitor) subunit 16B | -3.07 | 1.46 x 10^-10^ | 58 |
| 23585_at | PRAGMIN | homolog of rat pragma of Rnd2 | -1.66 | 6.55 x 10^-10^ | 99 |
| 224909_s_at | PREX1 | phosphatidylinositol-3,4,5-trisphosphate-dependent Rac exchange factor 1 | -1.54 | 2.18 x 10^-3^ | 89 |
| 203680_at | PRKAR2B | protein kinase, cAMP-dependent, regulatory, type II, beta | -1.76 | 3.11 x 10^-8^ | 100 |
| 20788_s_at | PROS1 | protein S (alpha) | -1.62 | 1.76 x 10^-5^ | 100 |
| 24919_at | PRR4 | proline rich 4 (lacrimal) | 1.90 | 2.37 x 10^-3^ | 96 |
| 24896_s_at | PTGER4 | prostaglandin E receptor 4 (subtype EP4) | -2.09 | 3.62 x 10^-3^ | 22 |
| 24897_at | PTGER4 | prostaglandin E receptor 4 (subtype EP4) | -1.53 | 1.23 x 10^-5^ | 100 |
| 213325_at | PVRL3 | poliovirus receptor-related 3 | -2.00 | 8.18 x 10^-6^ | 46 |
| 203498_at | RCAN2 | regulator of calcineurin 2 | -1.55 | 1.33 x 10^-3^ | 84 |
| 202975_s_at | RHOBTB3 | Rho-related BTB domain containing 3 | -1.60 | 2.70 x 10^-6^ | 100 |
| 202976_s_at | RHOBTB3 | Rho-related BTB domain containing 3 | -1.62 | 6.9 x 10^-8^ | 100 |
| 21648_s_at | RHOBTB3 | Rho-related BTB domain containing 3 | -1.59 | 3.63 x 10^-4^ | 97 |
| 225202_at | RHOBTB3 | Rho-related BTB domain containing 3 | -1.87 | 1.63 x 10^-11^ | 100 |
| 223168_at | RHOU | ras homolog gene family, member U | -1.57 | 7.6 x 10^-7^ | 100 |
| 22198_at | RNFT2 | ring finger protein, transmembrane 2 | 1.73 | 4.27 x 10^-8^ | 41 |
| 221909_at | RNFT2 | ring finger protein, transmembrane 2 | 2.79 | 8.31 x 10^-5^ | 34 |
| 230700_at | RTN4RL1 | reticulon 4 receptor-like 1 | -1.79 | 1.52 x 10^-5^ | 51 |
| 28607_s_at | SAA1 /// SAA2 | serum amyloid A1 /// serum amyloid A2 | -3.34 | 1.01 x 10^-6^ | 87 |
| 214456_ x _at | SAA1 /// SAA2 | serum amyloid A1 /// serum amyloid A2 | -3.57 | 4.15 x 10^-7^ | 50 |
| 207096_at | SAA4 | serum amyloid A4, constitutive | -2.30 | 1.6 x 10^-5^ | 93 |
| 20832_s_at | SCD | stearoyl-CoA desaturase (delta-9-desaturase) | -1.54 | 1.35 x 10^-3^ | 100 |
| 233565_s_at | SDCBP2 | syndecan binding protein (syntenin) 2 | 1.50 | 2.13 x 10^-6^ | 99 |
| 216346_at | SEC14L3 | SEC14-like 3 (S. cerevisiae) | -4.44 | 4.28 x 10^-11^ | 96 |
| 24699_at | SEC14L3 | SEC14-like 3 (S. cerevisiae) | -4.10 | 4.37 x 10^-14^ | 97 |
| 241221_at | SEC14L3 | CDNA FLJ60743 complete cds, highly similar to SEC14-like protein 3 | -3.61 | 7.64 x 10^-8^ | 35 |
| 215641_at | SEC24D | SEC24 family, member D (S. cerevisiae) | 1.59 | 3.16 x 10^-3^ | 76 |
| 1552789_at | SEC62 | SEC62 homolog (S. cerevisiae) | -1.51 | 6.59 x 10^-4^ | 98 |
| 229427_at | SEMA5A | sema domain, seven thrombospondin repeats (type 1 and type 1-like), transmembrane domain (TM) and short cytoplasmic domain, (semaphorin) 5A | -1.55 | 6.6 x 10^-5^ | 80 |
| 229620_at | SEPP1 | Selenoprotein P, plasma, 1, mRNA (cDNA clone MGC:47713 IMAGE:5770225) | -1.76 | 4.02 x 10^-6^ | 69 |
| 24614_at | SERPINB2 | serpin peptidase inhibitor, clade B (ovalbumin), member 2 | -1.83 | 5.95 x 10^-3^ | 93 |
| 209719_ x _at | SERPINB3 | serpin peptidase inhibitor, clade B (ovalbumin), member 3 | -1.80 | 4.45 x 10^-3^ | 100 |
| 209720_s_at | SERPINB3 | serpin peptidase inhibitor, clade B (ovalbumin), member 3 | -1.74 | 6.67 x 10^-3^ | 100 |
| 21413_ x _at | SERPINB3 /// SERPINB4 | serpin peptidase inhibitor, clade B (ovalbumin), member 3 /// serpin peptidase inhibitor, clade B (ovalbumin), member 4 | -1.75 | 4.90 x 10^-3^ | 100 |
| 200986_at | SERPING1 | serpin peptidase inhibitor, clade G (C1 inhibitor), member 1 | -1.63 | 8.48 x 10^-4^ | 100 |
| 223122_s_at | SFRP2 | secreted frizzled-related protein 2 | 4.56 | 1.9 x 10^-10^ | 50 |
| 24688_at | SGCE | sarcoglycan, epsilon | -1.56 | 1.83 x 10^-4^ | 90 |
| 244780_at | SGPP2 | sphingosine-1-phosphate phosphotase 2 | 1.51 | 6.97 x 10^-3^ | 98 |
| 228461_at | SH3RF3 | SH3 domain containing ring finger 3 | -1.67 | 6.44 x 10^-8^ | 100 |
| 227923_at | SHANK3 | SH3 and multiple ankyrin repeat domains 3 | -1.54 | 3.72 x 10^-3^ | 39 |
| 1552910_at | SIGLEC11 | sialic acid binding Ig-like lectin 11 | 2.07 | 5.71 x 10^-4^ | 23 |
| 219159_s_at | SLAMF7 | SLAM family member 7 | -1.69 | 2.90 x 10^-3^ | 38 |
| 222838_at | SLAMF7 | SLAM family member 7 | -1.63 | 2.9 x 10^-3^ | 89 |
| 28389_s_at | SLC1A2 | solute carrier family 1 (glial high affinity glutamate transporter), member 2 | 1.55 | 6.38 x 10^-4^ | 65 |
| 225491_at | SLC1A2 | solute carrier family 1 (glial high affinity glutamate transporter), member 2 | 1.78 | 8.87 x 10^-3^ | 70 |
| 26529_ x _at | SLC26A4 | solute carrier family 26, member 4 | -2.24 | 3.28 x 10^-3^ | 78 |
| 201801_s_at | SLC29A1 | solute carrier family 29 (nucleoside transporters), member 1 | -2.4 | 1.70 x 10^-6^ | 94 |
| 201802_at | SLC29A1 | solute carrier family 29 (nucleoside transporters), member 1 | -1.63 | 1.66 x 10^-6^ | 58 |
| 212907_at | SLC30A1 | Hbc647 mRNA sequence | -1.50 | 1.19 x 10^-5^ | 100 |
| 24124_at | SLC34A2 | solute carrier family 34 (sodium phosphate), member 2 | -1.56 | 2.79 x 10^-3^ | 99 |
| 209921_at | SLC7A11 | solute carrier family 7, (cationic amino acid transporter, y+ system) member 11 | 4.45 | 1.3 x 10^-14^ | 69 |
| 217678_at | SLC7A11 | solute carrier family 7, (cationic amino acid transporter, y+ system) member 11 | 7.07 | 3.71 x 10^-19^ | 85 |
| 209897_s_at | SLIT2 | slit homolog 2 (Drosophila) | -1.97 | 2.22 x 10^-8^ | 100 |
| 232176_at | SLITRK6 | SLIT and NTRK-like family, member 6 | -1.90 | 1.33 x 10^-7^ | 100 |
| 232481_s_at | SLITRK6 | SLIT and NTRK-like family, member 6 | -1.72 | 1.47 x 10^-9^ | 100 |
| 235976_at | SLITRK6 | SLIT and NTRK-like family, member 6 | -2.00 | 2.91 x 10^-9^ | 100 |
| 230782_at | SORD | sorbitol dehydrogenase | -1.63 | 9.97 x 10^-3^ | 74 |
| 202935_s_at | SOX9 | SRY (sex determining region Y)-box 9 | -1.61 | 3.67 x 10^-6^ | 100 |
| 202936_s_at | SOX9 | SRY (sex determining region Y)-box 9 | -1.83 | 5.16 x 10^-8^ | 100 |
| 1552396_at | SPINLW1 /// WFDC6 | serine peptidase inhibitor-like, with Kunitz and WAP domains 1 (eppin) /// WAP four-disulfide core domain 6 | -1.53 | 3.33 x 10^-4^ | 97 |
| 209875_s_at | SPP1 | secreted phosphoprotein 1 | 3.51 | 3.35 x 10^-5^ | 90 |
| 205499_at | SRPX2 | sushi-repeat-containing protein, X-linked 2 | 2.38 | 4.00 x 10^-8^ | 88 |
| 225252_at | SRXN1 | sulfiredoxin 1 homolog (S. cerevisiae) | 1.89 | 2.26 x 10^-10^ | 100 |
| 213355_at | ST3GAL6 | ST3 beta-galactoside alpha-2,3-sialyltransferase 6 | -1.52 | 4.6 x 10^-5^ | 79 |
| 220187_at | STEAP4 | STEAP family member 4 | -1.72 | 6.68 x 10^-4^ | 98 |
| 225987_at | STEAP4 | STEAP family member 4 | -1.62 | 1.68 x 10^-6^ | 100 |
| 212344_at | SULF1 | sulfatase 1 | -1.76 | 5.69 x 10^-5^ | 53 |
| 212353_at | SULF1 | sulfatase 1 | -1.57 | 8.17 x 10^-3^ | 91 |
| 212354_at | SULF1 | sulfatase 1 | -1.71 | 4.58 x 10^-6^ | 72 |
| 227480_at | SUSD2 | sushi domain containing 2 | -1.84 | 3.14 x 10^-5^ | 74 |
| 219389_at | SUSD4 | sushi domain containing 4 | -1.51 | 2.83 x 10^-4^ | 100 |
| 223821_s_at | SUSD4 | sushi domain containing 4 | -1.56 | 3.19 x 10^-5^ | 97 |
| 227662_at | SYNPO2 | synaptopodin 2 | -1.70 | 4.66 x 10^-3^ | 31 |
| 22686_at | SYT13 | synaptotagmin XIII | -1.65 | 9.70 x 10^-3^ | 54 |
| 229991_s_at | SYTL4 | Synaptotagmin-like 4 (SYTL4), transcript variant 2, mRNA | 1.69 | 4.14 x 10^-3^ | 20 |
| 221016_s_at | TCF7L1 | transcription factor 7-like 1 (T-cell specific, HMG-box) | -1.73 | 1.44 x 10^-9^ | 100 |
| 205513_at | TCN1 | transcobalamin I (vitamin B12 binding protein, R binder family) | 2.17 | 1.4 x 10^-4^ | 85 |
| 50221_at | TFEB | transcription factor EB | -1.69 | 1.6 x 10^-5^ | 92 |
| 205009_at | TFF1 | trefoil factor 1 | 2.00 | 7.64 x 10^-4^ | 94 |
| 24623_at | TFF3 | trefoil factor 3 (intestinal) | 1.78 | 5.27 x 10^-4^ | 100 |
| 209676_at | TFPI | tissue factor pathway inhibitor (lipoprotein-associated coagulation inhibitor) | -1.69 | 6.47 x 10^-4^ | 80 |
| 21664_s_at | TFPI | tissue factor pathway inhibitor (lipoprotein-associated coagulation inhibitor) | -1.64 | 1.13 x 10^-3^ | 76 |
| 213258_at | TFPI | tissue factor pathway inhibitor (lipoprotein-associated coagulation inhibitor) | -1.91 | 5.35 x 10^-5^ | 97 |
| 209278_s_at | TFPI2 | tissue factor pathway inhibitor 2 | -2.91 | 1.21 x 10^-6^ | 58 |
| 216262_s_at | TGIF2 | TGFB-induced factor homeobox 2 | -1.70 | 4.26 x 10^-4^ | 20 |
| 209561_at | THBS3 | thrombospondin 3 | -1.52 | 8.23 x 10^-7^ | 76 |
| 24776_at | THBS4 | thrombospondin 4 | -1.53 | 3.91 x 10^-3^ | 45 |
| 213894_at | THSD7A | thrombospondin, type I, domain containing 7A | -2.36 | 1.65 x 10^-6^ | 50 |
| 214920_at | THSD7A | thrombospondin, type I, domain containing 7A | -2.31 | 7.50 x 10^-11^ | 75 |
| 23008_at | THSD7A | thrombospondin, type I, domain containing 7A | -2.16 | 2.65 x 10^-5^ | 36 |
| 28699_ x _at | TKT | transketolase | 1.76 | 7.62 x 10^-5^ | 100 |
| 28700_s_at | TKT | transketolase | 1.73 | 6.13 x 10^-8^ | 100 |
| 224341_ x _at | TLR4 | toll-like receptor 4 | -1.54 | 8.52 x 10^-3^ | 76 |
| 222914_s_at | TMEM121 | transmembrane protein 121 | -1.82 | 4.43 x 10^-3^ | 51 |
| 229302_at | TMEM178 | transmembrane protein 178 | -1.70 | 5.01 x 10^-6^ | 100 |
| 219410_at | TMEM45A | transmembrane protein 45A | -2.40 | 3.7 x 10^-8^ | 100 |
| 209655_s_at | TMEM47 | transmembrane protein 47 | 1.64 | 5.86 x 10^-4^ | 99 |
| 241342_at | TMEM65 | transmembrane protein 65 | -1.71 | 2.20 x 10^-8^ | 100 |
| 209754_s_at | TMPO | thymopoietin | -1.50 | 2.5 x 10^-3^ | 99 |
| 202510_s_at | TNFAIP2 | tumor necrosis factor, alpha-induced protein 2 | -1.62 | 1.23 x 10^-5^ | 100 |
| 1569986_ x _at | TNNT3 | troponin T type 3 (skeletal, fast) | 1.54 | 2.25 x 10^-5^ | 83 |
| 205693_at | TNNT3 | troponin T type 3 (skeletal, fast) | 1.58 | 3.51 x 10^-6^ | 45 |
| 223686_at | TPK1 | thiamin pyrophosphokinase 1 | -1.62 | 6.79 x 10^-3^ | 27 |
| 2483_s_at | TPM2 | tropomyosin 2 (beta) | -1.96 | 1.40 x 10^-7^ | 76 |
| 202241_at | TRIB1 | tribbles homolog 1 (Drosophila) | -1.55 | 1.75 x 10^-4^ | 100 |
| 202478_at | TRIB2 | tribbles homolog 2 (Drosophila) | -1.51 | 1.82 x 10^-5^ | 100 |
| 24341_at | TRIM16 | tripartite motif-containing 16 | 1.88 | 4.60 x 10^-8^ | 100 |
| 227233_at | TSPAN2 | tetraspanin 2 | -1.79 | 4.93 x 10^-5^ | 84 |
| 227236_at | TSPAN2 | tetraspanin 2 | -1.58 | 7.15 x 10^-6^ | 100 |
| 229523_at | TTMA | CDNA clone IMAGE:9093264 | 1.51 | 1.02 x 10^-7^ | 100 |
| 22944_at | TWIST2 | twist homolog 2 (Drosophila) | -1.78 | 1.15 x 10^-3^ | 20 |
| 216609_at | TXN | Full length insert cDNA clone YI46D09 | 1.59 | 5.76 x 10^-4^ | 100 |
| 226747_at | TXNDC16 | thioredoxin domain containing 16 | -1.50 | 2.99 x 10^-7^ | 99 |
| 201266_at | TXNRD1 | thioredoxin reductase 1 | 1.95 | 1.33 x 10^-12^ | 100 |
| 201387_s_at | UCHL1 | ubiquitin carboxyl-terminal esterase L1 (ubiquitin thiolesterase) | 16.37 | 2.42 x 10^-23^ | 55 |
| 26094_ x _at | UGT1A1 /// UGT1A10 /// UGT1A3 /// UGT1A4 /// UGT1A5 /// UGT1A6 /// UGT1A7 /// UGT1A8 /// UGT1A9 | UDP glucuronosyltransferase 1 family, polypeptide A1 /// UDP glucuronosyltransferase 1 family, polypeptide A10 /// UDP glucuronosyltransferase 1 family, polypeptide A3 /// UDP glucuronosyltransferase 1 family, polypeptide A4 /// UDP glucuronosyltransferase 1 family, polypeptide A5 /// UDP glucuronosyltransferase 1 family, polypeptide A6 /// UDP glucuronosyltransferase 1 family, polypeptide A7 /// UDP glucuronosyltransferase 1 family, polypeptide A8 /// UDP glucuronosyltransferase 1 family, polypeptide A9 | 2.87 | 8.31 x 10^-17^ | 99 |
| 28596_s_at | UGT1A1 /// UGT1A10 /// UGT1A3 /// UGT1A4 /// UGT1A5 /// UGT1A6 /// UGT1A7 /// UGT1A8 /// UGT1A9 | UDP glucuronosyltransferase 1 family, polypeptide A1 /// UDP glucuronosyltransferase 1 family, polypeptide A10 /// UDP glucuronosyltransferase 1 family, polypeptide A3 /// UDP glucuronosyltransferase 1 family, polypeptide A4 /// UDP glucuronosyltransferase 1 family, polypeptide A5 /// UDP glucuronosyltransferase 1 family, polypeptide A6 /// UDP glucuronosyltransferase 1 family, polypeptide A7 /// UDP glucuronosyltransferase 1 family, polypeptide A8 /// UDP glucuronosyltransferase 1 family, polypeptide A9 | 3.94 | 1.17 x 10^-19^ | 99 |
| 215125_s_at | UGT1A1 /// UGT1A10 /// UGT1A3 /// UGT1A4 /// UGT1A5 /// UGT1A6 /// UGT1A7 /// UGT1A8 /// UGT1A9 | UDP glucuronosyltransferase 1 family, polypeptide A1 /// UDP glucuronosyltransferase 1 family, polypeptide A10 /// UDP glucuronosyltransferase 1 family, polypeptide A3 /// UDP glucuronosyltransferase 1 family, polypeptide A4 /// UDP glucuronosyltransferase 1 family, polypeptide A5 /// UDP glucuronosyltransferase 1 family, polypeptide A6 /// UDP glucuronosyltransferase 1 family, polypeptide A7 /// UDP glucuronosyltransferase 1 family, polypeptide A8 /// UDP glucuronosyltransferase 1 family, polypeptide A9 | 3.34 | 1.63 x 10^-16^ | 90 |
| 24532_ x _at | UGT1A1 /// UGT1A10 /// UGT1A4 /// UGT1A6 /// UGT1A8 /// UGT1A9 | UDP glucuronosyltransferase 1 family, polypeptide A1 /// UDP glucuronosyltransferase 1 family, polypeptide A10 /// UDP glucuronosyltransferase 1 family, polypeptide A4 /// UDP glucuronosyltransferase 1 family, polypeptide A6 /// UDP glucuronosyltransferase 1 family, polypeptide A8 /// UDP glucuronosyltransferase 1 family, polypeptide A9 | 2.79 | 1.10 x 10^-18^ | 100 |
| 207126_ x _at | UGT1A1 /// UGT1A10 /// UGT1A4 /// UGT1A6 /// UGT1A8 /// UGT1A9 | UDP glucuronosyltransferase 1 family, polypeptide A1 /// UDP glucuronosyltransferase 1 family, polypeptide A10 /// UDP glucuronosyltransferase 1 family, polypeptide A4 /// UDP glucuronosyltransferase 1 family, polypeptide A6 /// UDP glucuronosyltransferase 1 family, polypeptide A8 /// UDP glucuronosyltransferase 1 family, polypeptide A9 | 2.99 | 1.67 x 10^-18^ | 97 |
| 232654_s_at | UGT1A6 | UDP glucuronosyltransferase 1 family, polypeptide A6 | 1.89 | 2.49 x 10^-7^ | 55 |
| 28358_s_at | UGT8 | UDP glycosyltransferase 8 | -1.81 | 2.90 x 10^-3^ | 21 |
| 228956_at | UGT8 | Ceramide UDPgalactosyltransferase | -1.60 | 2.46 x 10^-3^ | 98 |
| 205356_at | USP13 | ubiquitin specific peptidase 13 (isopeptidase T-3) | -1.85 | 1.38 x 10^-15^ | 92 |
| 226902_at | USP13 | Isopeptidase T-3 (ISOT-3) | -1.75 | 2.22 x 10^-8^ | 51 |
| 232122_s_at | VEPH1 | ventricular zone expressed PH domain homolog 1 (zebrafish) | -1.62 | 4.17 x 10^-4^ | 89 |
| 215729_s_at | VGLL1 | vestigial like 1 (Drosophila) | -2.46 | 9.28 x 10^-4^ | 30 |
| 220327_at | VGLL3 | vestigial like 3 (Drosophila) | -2.36 | 1.30 x 10^-3^ | 57 |
| 227399_at | VGLL3 | vestigial like 3 (Drosophila) | -1.97 | 1.68 x 10^-3^ | 94 |
| 235751_s_at | VMO1 | vitelline membrane outer layer 1 homolog (chicken) | -1.62 | 3.64 x 10^-3^ | 97 |
| 212324_s_at | VPS13D | vacuolar protein sorting 13 homolog D (S. cerevisiae) | 1.56 | 9.15 x 10^-7^ | 43 |
| 212326_at | VPS13D | vacuolar protein sorting 13 homolog D (S. cerevisiae) | 1.56 | 7.7 x 10^-8^ | 99 |
| 227174_at | WDR72 | WD repeat domain 72 | 1.70 | 1.22 x 10^-3^ | 87 |
| 24712_at | WIF1 | WNT inhibitory factor 1 | -1.71 | 6.16 x 10^-3^ | 97 |
| 229158_at | WNK4 | WNK lysine deficient protein kinase 4 | -2.48 | 2.15 x 10^-9^ | 82 |
| 1552737_s_at | WWP2 | WW domain containing E3 ubiquitin protein ligase 2 | -1.90 | 5.93 x 10^-3^ | 41 |
| 223519_at | ZAK | sterile alpha motif and leucine zipper containing kinase AZK | -1.63 | 7.97 x 10^-7^ | 100 |
| 225662_at | ZAK | sterile alpha motif and leucine zipper containing kinase AZK | -1.53 | 4.41 x 10^-4^ | 100 |
| 225665_at | ZAK | sterile alpha motif and leucine zipper containing kinase AZK | -1.83 | 2.94 x 10^-10^ | 100 |
| 205883_at | ZBTB16 | zinc finger and BTB domain containing 16 | -1.83 | 7.6 x 10^-7^ | 58 |
| 1555793_a_at | ZFP82 | zinc finger protein 82 homolog (mouse) | -1.51 | 8.69 x 10^-7^ | 99 |
| 223589_at | ZNF416 | zinc finger protein 416 | -1.72 | 5.9 x 10^-4^ | 29 |
| 214761_at | ZNF423 | zinc finger protein 423 | -1.75 | 1.44 x 10^-4^ | 85 |
| 214746_s_at | ZNF467 | zinc finger protein 467 | 1.50 | 5.23 x 10^-5^ | 93 |
| 1553911_at | ZNF663 | zinc finger protein 663 | -1.55 | 8.69 x 10^-3^ | 44 |
| 235052_at | ZNF792 | zinc finger protein 792 | -1.61 | 1.31 x 10^-3^ | 26 |
| 1554636_at | --- | --- | -1.67 | 1.81 x 10^-3^ | 96 |
| 1555854_at | --- | --- | 3.93 | 5.01 x 10^-16^ | 100 |
| 1555929_s_at | --- | --- | 1.70 | 2.63 x 10^-4^ | 99 |
| 1556185_a_at | --- | --- | -1.61 | 5.21 x 10^-4^ | 96 |
| 1556602_at | --- | --- | -1.50 | 2.56 x 10^-3^ | 95 |
| 1556879_at | --- | --- | 2.39 | 5.9 x 10^-13^ | 100 |
| 1558687_a_at | --- | --- | -1.53 | 4.13 x 10^-4^ | 59 |
| 1559910_at | --- | --- | 1.66 | 3.56 x 10^-5^ | 25 |
| 156652_at | --- | --- | 1.60 | 4.59 x 10^-3^ | 98 |
| 1562472_at | --- | --- | 1.99 | 3.58 x 10^-4^ | 71 |
| 1563473_at | --- | --- | -1.67 | 1.23 x 10^-4^ | 52 |
| 1564358_at | --- | --- | 1.70 | 5.00 x 10^-3^ | 100 |
| 156640_at | --- | --- | 2.61 | 5.83 x 10^-4^ | 28 |
| 1566482_at | --- | --- | -2.51 | 1.85 x 10^-4^ | 29 |
| 1566597_at | --- | --- | 1.60 | 5.53 x 10^-3^ | 94 |
| 210524_ x _at | --- | --- | -1.63 | 1.02 x 10^-8^ | 100 |
| 215149_at | --- | --- | 2.07 | 1.96 x 10^-3^ | 21 |
| 217637_at | --- | --- | -1.62 | 1.77 x 10^-3^ | 25 |
| 222098_s_at | --- | --- | -1.68 | 6.36 x 10^-3^ | 22 |
| 224340_at | --- | --- | 1.57 | 2.29 x 10^-4^ | 70 |
| 224346_at | --- | --- | 1.96 | 2.73 x 10^-3^ | 29 |
| 226186_at | --- | --- | -1.53 | 2.33 x 10^-4^ | 30 |
| 226480_at | --- | --- | -1.57 | 3.59 x 10^-9^ | 100 |
| 227762_at | --- | --- | -1.68 | 6.35 x 10^-5^ | 66 |
| 227943_at | --- | --- | -1.51 | 1.77 x 10^-4^ | 100 |
| 228850_s_at | --- | --- | -2.02 | 7.56 x 10^-11^ | 98 |
| 228854_at | --- | --- | -2.51 | 7.97 x 10^-7^ | 89 |
| 22904_at | --- | --- | -1.70 | 2.27 x 10^-7^ | 73 |
| 22941_s_at | --- | --- | -1.88 | 1.60 x 10^-3^ | 37 |
| 229156_s_at | --- | --- | -1.90 | 1.47 x 10^-3^ | 33 |
| 229569_at | --- | --- | -1.56 | 5.64 x 10^-3^ | 40 |
| 229654_at | --- | --- | -2.57 | 1.37 x 10^-13^ | 95 |
| 230130_at | --- | --- | -2.16 | 8.92 x 10^-9^ | 100 |
| 230744_at | --- | --- | -1.69 | 4.01 x 10^-5^ | 39 |
| 230944_at | --- | --- | -1.87 | 2.06 x 10^-4^ | 22 |
| 231464_at | --- | --- | 2.36 | 5.81 x 10^-4^ | 32 |
| 231512_at | --- | --- | -1.85 | 9.74 x 10^-4^ | 28 |
| 232277_at | --- | --- | -1.74 | 4.04 x 10^-6^ | 87 |
| 232779_at | --- | --- | 1.77 | 6.83 x 10^-3^ | 27 |
| 232927_at | --- | --- | 1.53 | 6.76 x 10^-3^ | 88 |
| 233276_at | --- | --- | 1.95 | 1.58 x 10^-4^ | 49 |
| 23482_at | --- | --- | -2.72 | 5.77 x 10^-4^ | 57 |
| 234113_at | --- | --- | 1.58 | 8.86 x 10^-3^ | 29 |
| 234148_at | --- | --- | 1.64 | 1.56 x 10^-4^ | 77 |
| 235229_at | --- | --- | -1.64 | 8.01 x 10^-4^ | 94 |
| 235919_at | --- | --- | -1.64 | 1.45 x 10^-7^ | 100 |
| 235939_at | --- | --- | -1.57 | 1.82 x 10^-3^ | 42 |
| 23665_at | --- | --- | -1.95 | 1.44 x 10^-3^ | 26 |
| 236168_at | --- | --- | 1.64 | 5.02 x 10^-3^ | 73 |
| 236261_at | --- | --- | -1.57 | 3.82 x 10^-7^ | 100 |
| 236796_at | --- | --- | 1.52 | 1.43 x 10^-3^ | 76 |
| 237124_at | --- | --- | 1.70 | 7.23 x 10^-3^ | 81 |
| 237157_at | --- | --- | 1.60 | 4.55 x 10^-4^ | 99 |
| 237189_at | --- | --- | 1.56 | 4.98 x 10^-3^ | 67 |
| 237329_at | --- | --- | -1.64 | 2.45 x 10^-3^ | 43 |
| 237351_at | --- | --- | 4.79 | 1.40 x 10^-19^ | 71 |
| 237364_at | --- | --- | -1.70 | 2.81 x 10^-3^ | 21 |
| 237749_at | --- | --- | 3.01 | 1.49 x 10^-5^ | 20 |
| 238180_at | --- | --- | 2.38 | 7.27 x 10^-5^ | 62 |
| 238282_at | --- | --- | -1.55 | 1.13 x 10^-3^ | 62 |
| 238531_ x _at | --- | --- | -1.70 | 3.21 x 10^-3^ | 64 |
| 238835_at | --- | --- | -2.79 | 9.07 x 10^-3^ | 55 |
| 238861_at | --- | --- | -1.52 | 2.57 x 10^-5^ | 97 |
| 239021_at | --- | --- | 1.88 | 5.13 x 10^-3^ | 24 |
| 239171_at | --- | --- | 1.51 | 7.03 x 10^-4^ | 96 |
| 239229_at | --- | --- | 3.6 | 3.23 x 10^-10^ | 91 |
| 239557_at | --- | --- | 1.59 | 3.66 x 10^-3^ | 95 |
| 239986_at | --- | --- | 1.65 | 4.84 x 10^-4^ | 41 |
| 240242_at | --- | --- | -1.50 | 4.36 x 10^-7^ | 100 |
| 24454_at | --- | --- | 1.58 | 1.13 x 10^-3^ | 58 |
| 240788_at | --- | --- | 3.18 | 1.54 x 10^-9^ | 69 |
| 24869_at | --- | --- | -1.64 | 9.77 x 10^-8^ | 85 |
| 24160_ x _at | --- | --- | 1.95 | 6.98 x 10^-3^ | 22 |
| 241242_at | --- | --- | 1.65 | 3.53 x 10^-3^ | 90 |
| 241457_at | --- | --- | 1.86 | 2.11 x 10^-3^ | 95 |
| 241764_at | --- | --- | 6.44 | 7.56 x 10^-11^ | 43 |
| 242054_s_at | --- | --- | 2.49 | 2.64 x 10^-4^ | 45 |
| 242478_at | --- | --- | 1.67 | 9.09 x 10^-8^ | 100 |
| 242710_at | --- | --- | 2.00 | 2.78 x 10^-8^ | 97 |
| 242718_at | --- | --- | 1.54 | 8.08 x 10^-3^ | 34 |
| 242769_at | --- | --- | 1.81 | 1.01 x 10^-3^ | 67 |
| 242986_at | --- | --- | 1.67 | 7.66 x 10^-3^ | 43 |
| 243252_at | --- | --- | 1.67 | 1.88 x 10^-6^ | 100 |
| 243421_at | --- | --- | -3.97 | 3.07 x 10^-8^ | 56 |
| 243588_at | --- | --- | 1.79 | 1.79 x 10^-3^ | 58 |
| 243636_s_at | --- | --- | 1.56 | 4.33 x 10^-3^ | 90 |
| 243735_at | --- | --- | -1.52 | 1.38 x 10^-3^ | 84 |
| 243756_at | --- | --- | -2.09 | 6.61 x 10^-5^ | 50 |
| 243989_at | --- | --- | 1.59 | 6.14 x 10^-3^ | 43 |
| 244260_at | --- | --- | 2.01 | 2.19 x 10^-5^ | 90 |
| 244387_at | --- | --- | 1.56 | 2.21 x 10^-3^ | 100 |
| 244480_at | --- | --- | 1.70 | 5.33 x 10^-3^ | 30 |
| 244503_at | --- | --- | 2.34 | 6.21 x 10^-4^ | 34 |
| 244600_at | --- | --- | -1.90 | 7.64 x 10^-8^ | 76 |

^1^ Data obtained using the Affymetrix HG-U133 Plus 2.0 microarray chip.

^2^ Fold-change represents ratio of average expression value in healthy smokers to average expression value in healthy nonsmokers. Positive fold-changes represent genes upregulated by smoking; negative fold-changes represent genes down-regulated by smoking.

^3^ p value obtained using Benjamini-Hochberg correction to limit the false positive rate.

^4^ P call represents the % of healthy nonsmoker and healthy smoker samples in which the Affymetrix detection call for that probe set was “P” or “Present,” meaning that the gene was expressed in that sample.
