# Supplementary material for: Biologic Phenotyping of the Human Small Airway Epithelial Response to Cigarette Smoking
Source: PLoS One. 2011 Jul 28;6(7):e22798. doi: 10.1371/journal.pone.0022798 (PMC3145669; doi:10.1371/journal.pone.0022798)
Supplement: Table S3 — TaqMan confirmation of selected genes. (DOC) [file pone.0022798.s006.doc]

| **Gene symbol** | **Gene name** | **TaqMan fold-change (smoker/nonsmoker)^2^** | **TaqMan**  **p value^3^** | **Microarray fold-change (smoker/nonsmoker)^4^** | | **Microarray**  **p value^5^** | |  |
| --- | --- | --- | --- | --- | --- | --- | --- | --- |
| AKR1B10 | aldo-keto reductase family 1, member B10 | 65.06 | 4.41 x 10^-2^ | | 16.62 | | 1.55 x 10^-22^ | |
| CALCA | calcitonin-related polypeptide alpha | 9.50 | 3.04 x 10^-2^ | | 1.81 | | 2.74 x 10^-3^ | |
| CYP1B1 | cytochrome P450, family 1, subfamily B, polypeptide 1 | 89.39 | 2.27 x 10^-2^ | | 23.44 | | 2.42 x 10^-23^ | |
| NQO1 | NAD(P)H dehydrogenase, quinone 1 | 4.65 | 6.32 x 10^-4^ | | 3.21 | | 6.77 x 10^-18^ | |
| TCF7L1 | transcription factor 7-like 1 | -4.00 | 1.25 x 10^-3^ | | -1.73 | | 1.44 x 10^-9^ | |
| UCHL1 | ubiquitin carboxyl-terminal esterase L1 | 69.32 | 3.01 x 10^-2^ | | 16.37 | | 2.42 x 10^-23^ | |

^1^ Data obtained by TaqMan real-time RT-PCR for selected genes in n=14 healthy nonsmokers and n=14 healthy smokers, except CALCA (n=13 healthy nonsmokers and n=14 healthy smokers) and NQO1 (n=14 healthy nonsmokers and n=12 healthy smokers).

^2^ Fold-change represents ratio of average expression value in healthy smokers to average expression value in healthy nonsmokers as evaluated by TaqMan PCR. Positive fold-changes represent genes up-regulated by smoking; negative fold-changes represent genes down-regulated by smoking.

^3^ p value obtained via t-test.

^4^ Fold-change represents ratio of average expression value in healthy smokers to average expression value in healthy nonsmokers as evaluated by Affymetrix HG-U133 Plus 2.0 microarray analysis. Positive fold-changes represent genes up-regulated by smoking; negative fold-changes represent genes down-regulated by smoking.

^5^ p value obtained using Benjamini-Hochberg correction to limit the false positive rate
