# Supplementary material for: Biologic Phenotyping of the Human Small Airway Epithelial Response to Cigarette Smoking
Source: PLoS One. 2011 Jul 28;6(7):e22798. doi: 10.1371/journal.pone.0022798 (PMC3145669; doi:10.1371/journal.pone.0022798)
Supplement: Table S4 — Overlap of genes differentially expressed in the SAE of healthy smokers vs nonsmokers with other reported smoking responsive genes. (DOC) [file pone.0022798.s007.doc]

| **Genes differentially expressed in the SAE of healthy smokers *vs* nonsmokers** | **Identified in Spira smoker/nonsmoker analysis^2^** | **Identified in Zhang current smoker/former smoker analysis^3^** | **Identified in Zhang current smoker/never smoker analysis^4^** |
| --- | --- | --- | --- |
|  |  |  |  |
| AADAT |  |  |  |
| ABCA13 |  |  |  |
| ABCB6 |  |  |  |
| ABCC3 |  |  |  |
| ABHD2 | + |  |  |
| ABHD9 |  |  |  |
| ADAMTS3 |  |  |  |
| ADARB1 |  |  |  |
| ADH7 | + | + | + |
| AFAP1L1 |  |  |  |
| AGPAT9 |  |  |  |
| AGR2 |  | + | + |
| AJAP1 |  |  |  |
| AKAP7 |  |  |  |
| AKR1B1 | + | + | + |
| AKR1B10 |  | + | + |
| AKR1C1 | + | + | + |
| AKR1C1 | + | + | + |
| AKR1C2 | + | + | + |
| AKR1C3 | + | + | + |
| ALDH3A1 | + | + | + |
| ANGPT1 |  |  |  |
| ANO3 |  |  |  |
| ANO4 |  |  |  |
| ANPEP |  |  |  |
| ANTXR2 |  |  |  |
| AOC3 |  |  |  |
| APPL2 |  |  |  |
| ARHGAP4 |  |  |  |
| ARNT |  |  |  |
| ARRB1 |  |  |  |
| ASCL3 |  |  |  |
| ATAD4 |  |  |  |
| ATP12A |  |  |  |
| ATP13A4 |  |  |  |
| ATP6V0A4 |  |  |  |
| ATP6V1B1 |  |  |  |
| AVPR1A |  |  |  |
| AZGP1 |  |  |  |
| AZU1 |  |  |  |
| B3GNT6 |  |  |  |
| BAALC |  |  |  |
| BDH2 |  |  |  |
| BIRC3 |  |  |  |
| C3 |  | + | + |
| CA12 | + |  |  |
| CABYR |  | + | + |
| CACNG4 |  |  |  |
| CALCA |  |  |  |
| CBR1 | + | + | + |
| CBR3 |  | + | + |
| CCDC8 |  |  |  |
| CD44 |  |  |  |
| CD72 |  |  |  |
| CDC2 |  |  |  |
| CDC20B |  |  |  |
| CDC42EP3 |  |  |  |
| CDC42EP5 |  |  |  |
| CDH11 |  |  |  |
| CDH2 |  |  |  |
| CDKN1C |  |  |  |
| CEACAM5 |  | + | + |
| CEACAM6 | + |  |  |
| CENPM |  |  |  |
| CFD |  |  |  |
| CH25H |  | + |  |
| CHAD |  |  |  |
| CHEK1 |  |  |  |
| CHI3L1 |  |  |  |
| CHPT1 |  |  |  |
| CKB |  |  |  |
| CLCA4 |  |  |  |
| CLDN10 | + | + | + |
| CLDN8 |  |  |  |
| CLIP4 |  |  |  |
| CNGB1 |  |  |  |
| CNN3 |  |  |  |
| CNTD1 |  |  |  |
| CNTNAP3 |  |  |  |
| COL9A2 |  |  |  |
| CSGALNACT1 |  |  |  |
| CX3CL1 | + |  |  |
| CX3CL1 | + |  |  |
| CXCL3 |  |  |  |
| CYBRD1 |  |  |  |
| CYP1A1 |  | + | + |
| CYP1B1 | + | + | + |
| CYP26A1 |  |  |  |
| CYP2A6 |  | + | + |
| CYP4F11 | + | + | + |
| CYP4F3 |  | + |  |
| CYP4X1 |  |  |  |
| DAPK1 |  |  |  |
| DEFB1 |  | + | + |
| DEPDC6 |  |  |  |
| DNAJC12 |  |  |  |
| DPYSL2 |  |  |  |
| DRD1 |  |  |  |
| DSG2 |  |  |  |
| DTNA |  |  |  |
| DUOX2 |  |  |  |
| DUSP1 |  |  |  |
| DUSP5 |  |  |  |
| EFHD1 |  |  |  |
| EFNB2 |  |  |  |
| EGF |  |  |  |
| EGFL6 |  |  |  |
| EID3 |  |  |  |
| ELFN2 |  |  |  |
| ELMOD1 |  |  |  |
| EPB41L2 |  |  |  |
| EPB41L3 |  |  |  |
| EPHA4 |  |  |  |
| EPHB1 |  |  |  |
| ERP27 |  |  |  |
| EVC |  |  |  |
| FABP6 |  | + | + |
| FBN1 |  |  |  |
| FBXO42 |  |  |  |
| FBXW10 |  |  |  |
| FGFR2 |  |  |  |
| FGFR3 |  |  |  |
| FHOD3 |  |  |  |
| FMO2 |  |  |  |
| FMOD |  |  |  |
| FNIP2 |  |  |  |
| FOLR1 |  |  |  |
| FOXA2 |  |  |  |
| FRMD4A |  |  |  |
| FXYD6 |  |  |  |
| FZD8 |  |  |  |
| G0S2 |  |  |  |
| G6PD |  |  |  |
| GAD1 |  |  |  |
| GADD45B |  |  |  |
| GALNT5 |  |  |  |
| GALNT6 | + |  |  |
| GCLC | + | + | + |
| GDA |  |  |  |
| GEM |  |  |  |
| GLB1L3 |  |  |  |
| GLI3 |  |  |  |
| GLRB |  |  |  |
| GPR115 |  |  |  |
| GPR125 |  |  |  |
| GPX2 | + | + |  |
| GRM5 |  |  |  |
| GSR |  |  |  |
| H19 |  |  |  |
| HEPACAM2 |  |  |  |
| HES6 |  |  |  |
| HEY2 |  |  |  |
| HIP1 |  |  |  |
| HIVEP3 |  |  |  |
| HLA-DQB2 |  | + | + |
| HLF | + |  |  |
| HLF | + |  |  |
| HNMT |  |  |  |
| HOPX |  |  |  |
| HOXA1 |  |  |  |
| HOXC4 |  |  |  |
| HRK |  |  |  |
| HS6ST2 |  |  |  |
| HSD17B2 |  |  |  |
| HSPA2 | + |  |  |
| HTATIP2 | + | + | + |
| IDS |  |  |  |
| IGSF22 |  |  |  |
| IL27RA |  |  |  |
| IL4R |  |  |  |
| IRF2BP2 |  |  |  |
| IRS2 |  |  |  |
| ITGA2 |  |  |  |
| ITGA9 |  |  |  |
| ITLN1 |  |  |  |
| ITM2A |  | + | + |
| JAKMIP3 |  |  |  |
| KCNA1 |  |  |  |
| KCNB1 |  |  |  |
| KCNC4 |  |  |  |
| KCNMA1 |  |  |  |
| KCNMB2 |  |  |  |
| KLHL6 |  |  |  |
| KRT4 |  |  |  |
| LAMB3 |  |  |  |
| LEPR |  |  |  |
| LHX6 |  |  |  |
| LMNB1 |  |  |  |
| LOC54492 |  |  |  |
| LRP2 |  |  |  |
| LRRC31 |  |  |  |
| LRRN1 |  |  |  |
| LTBP1 |  |  |  |
| LTF |  |  |  |
| MAGI2 |  |  |  |
| MALAT1 |  |  |  |
| MALL |  |  |  |
| MAOB |  | + | + |
| MAP1B |  |  |  |
| MAP3K1 |  |  |  |
| MARCKSL1 |  |  |  |
| MB |  | + | + |
| MCOLN2 |  |  |  |
| MCOLN3 |  |  |  |
| MDC1 |  |  |  |
| ME1 | + | + | + |
| ME1 | + | + | + |
| MEF2C |  |  |  |
| MFSD2 |  |  |  |
| MGC34774 |  |  |  |
| MIPOL1 |  |  |  |
| MMP7 |  |  |  |
| MPPED2 |  |  |  |
| MSMB | + | + | + |
| MSRB3 |  |  |  |
| MT1E |  |  |  |
| MT1F | + |  |  |
| MT1G | + |  |  |
| MT1H |  |  |  |
| MT1P2 |  |  |  |
| MT1X | + |  |  |
| MT2A |  |  |  |
| MT3 |  |  |  |
| MTHFD1 |  |  |  |
| MTL5 |  |  |  |
| MUC5AC |  | + | + |
| MUCL1 |  |  |  |
| MYLK |  |  |  |
| NAV1 |  |  |  |
| NAV3 |  |  |  |
| NCOA7 |  |  |  |
| NFASC |  |  |  |
| NFE2L3 |  |  |  |
| NFKBIA |  |  |  |
| NOL3 |  |  |  |
| NOVA1 |  |  |  |
| NPAS3 |  |  |  |
| NQO1 |  | + | + |
| NR0B1 |  |  |  |
| NR4A3 |  |  |  |
| NRXN1 |  |  |  |
| NT5E |  |  |  |
| NXN |  |  |  |
| PANK1 |  |  |  |
| PANX2 |  |  |  |
| PAPSS2 |  |  |  |
| PAX1 |  |  |  |
| PCDH17 |  |  |  |
| PCDH20 |  |  |  |
| PCSK1N |  |  |  |
| PCSK6 |  |  |  |
| PCSK9 |  |  |  |
| PDK4 |  |  |  |
| PDLIM4 |  |  |  |
| PDZK1IP1 |  |  |  |
| PEG10 |  |  |  |
| PEX13 |  |  |  |
| PFTK1 |  |  |  |
| PGD | + | + | + |
| PHEX |  |  |  |
| PHLDA1 |  |  |  |
| PI3 |  |  |  |
| PIP5K1B |  | + |  |
| PIR | + | + | + |
| PKIB |  |  |  |
| PLA2G10 | + |  |  |
| PLAT |  |  |  |
| PLEKHG4B |  |  |  |
| PLG |  |  |  |
| PLK2 |  |  |  |
| PLK4 |  |  |  |
| PNRC1 |  |  |  |
| POU2AF1 |  |  |  |
| PPAP2B |  |  |  |
| PPP1R16B |  |  |  |
| PRAGMIN |  |  |  |
| PREX1 |  |  |  |
| PRKAR2B |  |  |  |
| PROS1 |  | + | + |
| PRR4 |  |  |  |
| PTGER4 |  | + |  |
| PVRL3 |  |  |  |
| RCAN2 |  |  |  |
| RHOBTB3 |  |  |  |
| RHOU |  |  |  |
| RNFT2 |  |  |  |
| RTN4RL1 |  |  |  |
| SAA4 |  | + |  |
| SCD |  |  |  |
| SDCBP2 |  |  |  |
| SEC14L3 |  |  |  |
| SEC24D |  |  |  |
| SEC62 |  |  |  |
| SEMA5A |  |  |  |
| SEPP1 |  |  |  |
| SERPINB2 |  |  |  |
| SERPINB3 |  |  |  |
| SERPING1 |  | + | + |
| SFRP2 |  |  |  |
| SGCE |  |  |  |
| SGPP2 |  |  |  |
| SH3RF3 |  |  |  |
| SHANK3 |  |  |  |
| SIGLEC11 |  |  |  |
| SLAMF7 |  |  |  |
| SLC1A2 |  |  |  |
| SLC26A4 |  |  |  |
| SLC29A1 |  |  |  |
| SLC30A1 |  |  |  |
| SLC34A2 |  |  |  |
| SLC7A11 |  | + | + |
| SLIT2 | + | + | + |
| SLITRK6 |  |  |  |
| SORD |  |  |  |
| SOX9 |  | + |  |
| SPP1 |  |  |  |
| SRPX2 |  |  |  |
| SRXN1 |  |  |  |
| ST3GAL6 |  |  |  |
| STEAP4 |  |  |  |
| SULF1 |  |  |  |
| SUSD2 |  |  |  |
| SUSD4 |  |  |  |
| SYNPO2 |  |  |  |
| SYT13 |  |  |  |
| SYTL4 |  |  |  |
| TCF7L1 |  |  |  |
| TCN1 | + | + | + |
| TFEB |  |  |  |
| TFF1 |  | + | + |
| TFF3 |  | + | + |
| TFPI |  |  |  |
| TFPI2 |  |  |  |
| TGIF2 |  |  |  |
| THBS3 |  |  |  |
| THBS4 |  |  |  |
| THSD7A |  |  |  |
| TKT | + | + | + |
| TLR4 |  |  |  |
| TMEM121 |  |  |  |
| TMEM178 |  |  |  |
| TMEM45A |  |  |  |
| TMEM47 |  |  |  |
| TMEM65 |  |  |  |
| TMPO |  |  |  |
| TNFAIP2 |  |  |  |
| TNNT3 |  |  |  |
| TPK1 |  |  |  |
| TPM2 |  |  |  |
| TRIB1 |  |  |  |
| TRIB2 |  |  |  |
| TRIM16 | + | + | + |
| TSPAN2 |  |  |  |
| TTMA |  |  |  |
| TWIST2 |  |  |  |
| TXN | + | + | + |
| TXNDC16 |  |  |  |
| TXNRD1 | + | + | + |
| UCHL1 |  | + | + |
| UGT1A6 |  |  |  |
| UGT8 |  |  |  |
| USP13 |  |  |  |
| VEPH1 |  |  |  |
| VGLL1 |  |  |  |
| VGLL3 |  |  |  |
| VMO1 |  |  |  |
| VPS13D |  |  |  |
| WDR72 |  |  |  |
| WIF1 |  |  |  |
| WNK4 |  |  |  |
| WWP2 |  |  |  |
| ZAK |  |  |  |
| ZBTB16 |  |  |  |
| ZFP82 |  |  |  |
| ZNF416 |  |  |  |
| ZNF423 |  |  |  |
| ZNF467 |  |  |  |
| ZNF663 |  |  |  |
| ZNF792 |  |  |  |

^1^ All unique gene symbols identified in the analysis of SAE gene expression in healthy smokers *vs* nonsmokers were compared with the smoking-responsive gene lists reported by Spira et al^1^ and Zhang et al^2^.

^2^ “+” indicates a gene that was also identified as a smoking-responsive gene by Spira et al^1^.

^3^ “+” indicates a gene that was identified as a smoking-responsive gene in the analysis of current *vs* former smokers reported by Zhang et al^2^.

^4^ “+” indicates a gene that was identified as a smoking-responsive gene in the analysis of current *vs* never smokers reported by Zhang et al^2^.

**References**

1. A. Spira, J. Beane, V. Shah, G. Liu, F. Schembri, X. Yang, J. Palma, J. S. Brody, Effects of cigarette smoke on the human airway epithelial cell transcriptome. *Proc. Natl. Acad. Sci U. S. A* 101, 10143-10148 (2004).

2. L. I. Zhang, J. Lee, H. Tang, Y. H. Fan, L. Xiao, H. Ren, J. Kurie, R. C. Morice, W. K. Hong, H. Mao, Impact of smoking cessation on global gene expression in the bronchial epithelium of chronic smokers. *Cancer Prev Res* 1, 112-118 (2008).
