# Supplementary material for: Biologic Phenotyping of the Human Small Airway Epithelial Response to Cigarette Smoking
Source: PLoS One. 2011 Jul 28;6(7):e22798. doi: 10.1371/journal.pone.0022798 (PMC3145669; doi:10.1371/journal.pone.0022798)
Supplement: Table S5 — Characteristics of the ISAE among the study population. (DOC) [file pone.0022798.s008.doc]

**Table S5. Characteristics of the I_SAE_ Among the Study Population^1^**

| **Category** | **Median I_SAE_** | **Variance** | **Interquartile range^2^** | **p value *vs* NS^3^** |
| --- | --- | --- | --- | --- |
| Healthy nonsmokers | 1.1 | 5.8 | 0.7 - 2.9 | - |
| Healthy smokers | 23.6 | 109.4 | 16.8 - 29.6 | <0.0001 |
| Low responders | 16.8 | 39.9 | 9.9 - 19.7 | <0.0001 |
| High responders | 29.6 | 38.8 | 26.9 - 34.7 | <0.0001 |

^1^ Statistics were calculated for the I_SAE_ within the phenotypic groups of healthy nonsmokers and healthy smokers, as well as for the high responder

and low responder subgroups of healthy smokers based on the median I_SAE_ value.

^2^ The interquartile range represents the range from the 25^th^ percentile to the 75^th^ percentile of values.

^3^ p values for differences in the medians were calculated using a Mann Whitney U test for pairwise comparisons.
