# Supplementary material for: Biologic Phenotyping of the Human Small Airway Epithelial Response to Cigarette Smoking
Source: PLoS One. 2011 Jul 28;6(7):e22798. doi: 10.1371/journal.pone.0022798 (PMC3145669; doi:10.1371/journal.pone.0022798)
Supplement: Table S6 — Demographics of low responder and high responder healthy smokers. (DOC) [file pone.0022798.s009.doc]

| **Parameter** | **Low Responders** | **High Responders** | **p value^2^** |
| --- | --- | --- | --- |
| n | 29 | 29 |  |
| Sex (male/female) | 19/10 | 19/10 | 1.000 |
| Age | 41.6 ± 7.7 | 44.2 ± 6.7 | 0.182 |
| Race (B/W/O)^3^ | 20/3/6 | 15/11/3 | 0.111 |
| Smoking history (pack-yr) | 24.1 ± 17.0 | 30.9 ± 15.9 | 0.117 |
| Yr of smoking | 22.4 ± 10.5 | 26.5 ± 10.4 | 0.225 |
| Urine nicotine (ng/ml) | 754 ± 1165 | 1840 ± 1975 | 0.017 |
| Urine cotinine (ng/ml) | 958 ± 912 | 1533 ± 954 | 0.025 |
| Blood carboxyhemoglobin (%) | 1 ± 1 | 2 ± 2 | 0.012 |
| Pulmonary function parameters^4^ |  |  |  |
| FVC | 109 ± 13 | 108 ± 14 | 0.820 |
| FEV1 % predicted | 109 ± 12 | 105 ± 16 | 0.324 |
| FEV1/FVC % observed | 82 ± 5 | 79 ± 5 | 0.070 |
| FEF25-75 | 91 ± 19 | 85 ± 19 | 0.271 |
| TLC | 100 ± 13 | 100 ± 11 | 0.975 |
| DLCO %predicted | 96 ± 14 | 92 ± 7 | 0.118 |
| Epithelial cells^5^ |  |  |  |
| Number recovered x 10^6^ | 7.0 ± 2.9 | 7.5 ± 3.2 | 0.541 |
| % epithelial cells | 99.3 ± 1.3 | 99.0 ± 1.3 | 0.273 |
| % inflammatory cells | 0.7 ± 1.3 | 1.0 ± 1.3 | 0.277 |
| Differential cell count^6^ |  |  |  |
| Ciliated (%) | 68.2 ± 11.3 | 63.0 ± 13.4 | 0.130 |
| Secretory (%) | 8.1 ± 3.5 | 10.1 ± 5.3 | 0.105 |
| Basal (%) | 12.6 ± 7.6 | 12.9 ± 5.7 | 0.840 |
| Undifferentiated columnar (%) | 10.6 ± 4.9 | 13.2 ± 8.1 | 0.161 |

^1^ Data are presented as mean ± standard deviation. Low responders are healthy smokers with I_SAE_ below the median and high responders are those with I_SAE_ above the median.

^2^ p values were calculated by Student’s t test for continuous variables and chi square for categorial variables. When corrected for multiple testing, p=0.002 represents a significant difference.

^3^ B = black, W = white, O = other.

^4^ Pulmonary function testing parameters are given as % of predicted value with the exception of FEV1/FVC, which is reported as % observed; FVC - forced vital capacity, FEV1 - forced expiratory volume in 1 sec, TLC - total lung capacity, DLCO - diffusing capacity.

^5^ Small airway epithelium.

^6^ As a % of small airway epithelium recovered.
