# Supplementary material for: Biologic Phenotyping of the Human Small Airway Epithelial Response to Cigarette Smoking
Source: PLoS One. 2011 Jul 28;6(7):e22798. doi: 10.1371/journal.pone.0022798 (PMC3145669; doi:10.1371/journal.pone.0022798)
Supplement: Table S7 — Genes differentially expressed in the small airway epithelium of high responder healthy smokers vs low responder healthy smokers. (DOC) [file pone.0022798.s010.doc]

| **Probe set ID** | **Gene symbol** | **Gene title** | **Fold-change (high responder healthy smokers/low responder healthy smokers)^2^** | **p value^3^** | **Smoking-responsive probe set?**^4^ |
| --- | --- | --- | --- | --- | --- |
|  |  |  |  |  |  |
| 206561_s_at | AKR1B10 | aldo-keto reductase family 1, member B10 (aldose reductase) | 4.10 | 4.76 x 10^-3^ | yes |
| 208498_s_at | AMY1A /// AMY1B /// AMY1C /// AMY2A /// AMY2B | amylase, alpha 1A (salivary) /// amylase, alpha 1B (salivary) /// amylase, alpha 1C (salivary) /// amylase, alpha 2A (pancreatic) /// amylase, alpha 2B (pancreatic) | -1.60 | 8.08 x 10^-3^ | yes |
| 206164_at | CLCA2 | chloride channel regulator 2 | -1.82 | 9.06 x 10^-3^ | no |
| 218002_s_at | CXCL14 | chemokine (C-X-C motif) ligand 14 | 4.13 | 3.65 x 10^-3^ | no |
| 206515_at | CYP4F3 | cytochrome P450, family 4, subfamily F, polypeptide 3 | 2.26 | 9.06 x 10^-3^ | yes |
| 227702_at | CYP4X1 | cytochrome P450, family 4, subfamily X, polypeptide 1 | -1.50 | 5.78 x 10^-3^ | yes |
| 214652_at | DRD1 | dopamine receptor D1 | -3.41 | 8.73 x 10^-3^ | yes |
| 226432_at | ETNK1 | ethanolamine kinase 1 | -1.51 | 5.78 x 10^-3^ | no |
| 22306_at | FAM46C | family with sequence similarity 46, member C | -1.61 | 9.62 x 10^-3^ | no |
| 229623_at | FLJ12993 | Hypothetical LOC441027 (FLJ12993), mRNA | -1.52 | 8.08 x 10^-3^ | no |
| 209305_s_at | GADD45B | growth arrest and DNA-damage-inducible, beta | -1.56 | 5.84 x 10^-3^ | yes |
| 214290_s_at | HIST2H2AA3 /// HIST2H2AA4 | histone cluster 2, H2aa3 /// histone cluster 2, H2aa4 | -1.69 | 4.76 x 10^-3^ | no |
| 218280_x_at | HIST2H2AA3 /// HIST2H2AA4 | histone cluster 2, H2aa3 /// histone cluster 2, H2aa4 | -1.52 | 4.76 x 10^-3^ | no |
| 223597_at | ITLN1 | intelectin 1 (galactofuranose binding) | -3.90 | 7.33 x 10^-3^ | yes |
| 230849_at | KCNA1 | potassium voltage-gated channel, shaker-related subfamily, member 1 (episodic ataxia with myokymia) | -1.86 | 8.82 x 10^-3^ | yes |
| 1553674_at | LRRIQ3 | leucine-rich repeats and IQ motif containing 3 | -1.53 | 7.61 x 10^-3^ | no |
| 204041_at | MAOB | monoamine oxidase B | -2.05 | 1.97 x 10^-3^ | yes |
| 229281_at | NPAS3 | neuronal PAS domain protein 3 | -1.88 | 4.78 x 10^-3^ | yes |
| 1553995_a_at | NT5E | 5'-nucleotidase, ecto (CD73) | -2.16 | 8.08 x 10^-3^ | yes |
| 23939_at | NT5E | 5'-nucleotidase, ecto (CD73) | -1.73 | 3.81 x 10^-3^ | yes |
| 224046_s_at | PDE7A | phosphodiesterase 7A | -1.64 | 6.80 x 10^-3^ | no |
| 212094_at | PEG10 | paternally expressed 10 | -1.70 | 7.78 x 10^-3^ | yes |
| 201120_s_at | PGRMC1 | progesterone receptor membrane component 1 | -1.64 | 8.61 x 10^-3^ | no |
| 217996_at | PHLDA1 | pleckstrin homology-like domain, family A, member 1 | 1.62 | 7.33 x 10^-3^ | yes |
| 23680_at | PRKAR2B | protein kinase, cAMP-dependent, regulatory, type II, beta | -1.63 | 5.3 x 10^-3^ | yes |
| 209921_at | SLC7A11 | solute carrier family 7, (cationic amino acid transporter, y+ system) member 11 | 2.60 | 7.33 x 10^-3^ | yes |
| 217678_at | SLC7A11 | solute carrier family 7, (cationic amino acid transporter, y+ system) member 11 | 3.07 | 5.15 x 10^-3^ | yes |
| 232176_at | SLITRK6 | SLIT and NTRK-like family, member 6 | -1.90 | 1.97 x 10^-3^ | yes |
| 232481_s_at | SLITRK6 | SLIT and NTRK-like family, member 6 | -1.57 | 2.75 x 10^-3^ | yes |
| 1552396_at | SPINLW1 /// WFDC6 | serine peptidase inhibitor-like, with Kunitz and WAP domains 1 (eppin) /// WAP four-disulfide core domain 6 | -1.68 | 7.78 x 10^-3^ | yes |
| 205499_at | SRPX2 | sushi-repeat-containing protein, X-linked 2 | 1.98 | 3.81 x 10^-3^ | yes |
| 220187_at | STEAP4 | STEAP family member 4 | -2.07 | 4.76 x 10^-3^ | yes |
| 225987_at | STEAP4 | STEAP family member 4 | -1.62 | 4.76 x 10^-3^ | yes |
| 221727_at | SUB1 | SUB1 homolog (S. cerevisiae) | -1.58 | 5.84 x 10^-3^ | no |
| 219410_at | TMEM45A | transmembrane protein 45A | -2.01 | 9.19 x 10^-3^ | yes |
| 225665_at | ZAK | sterile alpha motif and leucine zipper containing kinase AZK | -1.59 | 1.97 x 10^-3^ | yes |
| 1554007_at | --- | --- | -1.60 | 8.08 x 10^-3^ | no |
| 229654_at | --- | --- | -2.04 | 1.49 x 10^-3^ | yes |

^1^ Data obtained using the Affymetrix HG-U133 Plus 2.0 microarray chip.

^2^ Fold-change represents ratio of average expression value in high responder healthy smokers to average expression value in low responder healthy smokers. Positive fold-changes represent genes more highly expressed in high responders; negative fold-changes represent genes more highly expressed in low responders.

^3^ p value obtained using Benjamini-Hochberg correction to limit the false positive rate.

^4^ “Yes” indicates a probe set that was found to be significantly modulated by smoking (i.e., one of the 647 probe sets significantly differentially expressed in healthy smokers *vs* healthy nonsmokers in Table S1). “No” indicates a probe set that was not identified as smoking-responsive.
