# Supplementary material for: Biologic Phenotyping of the Human Small Airway Epithelial Response to Cigarette Smoking
Source: PLoS One. 2011 Jul 28;6(7):e22798. doi: 10.1371/journal.pone.0022798 (PMC3145669; doi:10.1371/journal.pone.0022798)
Supplement: Table S8 — Genes differentially expressed in the small airway epithelium of COPD smokers vs low responder healthy smokers. (DOC) [file pone.0022798.s011.doc]

| **Probe set ID** | **Gene symbol** | **Gene title** | **Fold-change (COPD smokers/low responder healthy smokers)^2^** | **p value^3^** |
| --- | --- | --- | --- | --- |
|  |  |  |  |  |
| 202912_at | ADM | adrenomedullin | 2.67 | 2.21 x 10^-3^ |
| 229354_at | AHRR /// PDCD6 | aryl-hydrocarbon receptor repressor /// programmed cell death 6 | 2.80 | 2.30 x 10^-3^ |
| 227530_at | AKAP12 | A kinase (PRKA) anchor protein 12 | 2.18 | 8.07 x 10^-3^ |
| 26561_s_at | AKR1B10 | aldo-keto reductase family 1, member B10 (aldose reductase) | 3.74 | 8.07 x 10^-3^ |
| 209369_at | ANXA3 | annexin A3 | 1.58 | 1.31 x 10^-3^ |
| 221161_at | ASCL3 | achaete-scute complex homolog 3 (Drosophila) | 1.88 | 3.88 x 10^-3^ |
| 214070_s_at | ATP10B | ATPase, class V, type 10B | 1.86 | 5.45 x 10^-5^ |
| 208836_at | ATP1B3 | ATPase, Na+/K+ transporting, beta 3 polypeptide | 1.56 | 4.37 x 10^-3^ |
| 231126_at | C2orf70 | chromosome 2 open reading frame 70 | 1.52 | 6.17 x 10^-3^ |
| 216598_s_at | CCL2 | chemokine (C-C motif) ligand 2 | 5.98 | 1.23 x 10^-3^ |
| 226545_at | CD109 | CD109 molecule | 1.96 | 5.45 x 10^-5^ |
| 229900_at | CD109 | CD109 molecule | 2.62 | 1.93 x 10^-4^ |
| 208653_s_at | CD164 | CD164 molecule, sialomucin | -1.61 | 8.27 x 10^-3^ |
| 21263_at | CD44 | CD44 molecule (Indian blood group) | 1.64 | 2.39 x 10^-3^ |
| 201884_at | CEACAM5 | carcinoembryonic antigen-related cell adhesion molecule 5 | 3.10 | 1.43 x 10^-6^ |
| 202712_s_at | CKMT1A /// CKMT1B /// LOC100133623 | creatine kinase, mitochondrial 1A /// creatine kinase, mitochondrial 1B /// similar to Creatine kinase, ubiquitous mitochondrial precursor (U-MtCK) (Mia-CK) (Acidic-type mitochondrial creatine kinase) | 1.57 | 2.39 x 10^-3^ |
| 26164_at | CLCA2 | chloride channel regulator 2 | -1.88 | 3.11 x 10^-3^ |
| 25328_at | CLDN10 | claudin 10 | 1.76 | 1.43 x 10^-3^ |
| 22332_at | CLDN16 | claudin 16 | -1.88 | 8.58 x 10^-3^ |
| 219890_at | CLEC5A | C-type lectin domain family 5, member A | 4.33 | 8.27 x 10^-4^ |
| 24971_at | CSTA | cystatin A (stefin A) | 1.82 | 1.48 x 10^-3^ |
| 218002_s_at | CXCL14 | chemokine (C-X-C motif) ligand 14 | 7.70 | 2.47 x 10^-5^ |
| 222484_s_at | CXCL14 | chemokine (C-X-C motif) ligand 14 | 6.67 | 1.48 x 10^-3^ |
| 25749_at | CYP1A1 | cytochrome P450, family 1, subfamily A, polypeptide 1 | 11.58 | 4.08 x 10^-3^ |
| 202435_s_at | CYP1B1 | cytochrome P450, family 1, subfamily B, polypeptide 1 | 4.52 | 1.86 x 10^-3^ |
| 202436_s_at | CYP1B1 | cytochrome P450, family 1, subfamily B, polypeptide 1 | 4.87 | 1.27 x 10^-3^ |
| 202437_s_at | CYP1B1 | cytochrome P450, family 1, subfamily B, polypeptide 1 | 4.09 | 6.75 x 10^-3^ |
| 21397_at | DEFB1 | defensin, beta 1 | 2.11 | 6.36 x 10^-3^ |
| 24687_at | DKFZP564O0823 | DKFZP564O0823 protein | 1.72 | 9.09 x 10^-3^ |
| 226863_at | FAM110C | family with sequence similarity 110, member C | 1.60 | 1.79 x 10^-3^ |
| 227194_at | FAM3B | family with sequence similarity 3, member B | 2.15 | 5.45 x 10^-5^ |
| 210889_s_at | FCGR2B | Fc fragment of IgG, low affinity IIb, receptor (CD32) | 3.42 | 3.22 x 10^-3^ |
| 25014_at | FGFBP1 | fibroblast growth factor binding protein 1 | 2.6 | 9.28 x 10^-4^ |
| 218980_at | FHOD3 | formin homology 2 domain containing 3 | -2.20 | 2.21 x 10^-3^ |
| 25278_at | GAD1 | glutamate decarboxylase 1 (brain, 67kDa) | 2.67 | 2.78 x 10^-3^ |
| 26669_at | GAD1 | glutamate decarboxylase 1 (brain, 67kDa) | 2.27 | 8.41 x 10^-3^ |
| 217755_at | HN1 | hematological and neurological expressed 1 | 1.52 | 4.20 x 10^-3^ |
| 230849_at | KCNA1 | potassium voltage-gated channel, shaker-related subfamily, member 1 (episodic ataxia with myokymia) | -2.36 | 8.41 x 10^-3^ |
| 209125_at | KRT6A | keratin 6A | 5.12 | 1.46 x 10^-4^ |
| 1557207_s_at | LOC283177 | hypothetical protein LOC283177 | -2.00 | 1.48 x 10^-3^ |
| 202018_s_at | LTF | lactotransferrin | -3.64 | 8.07 x 10^-3^ |
| 202291_s_at | MGP | matrix Gla protein | -2.88 | 8.07 x 10^-3^ |
| 214385_s_at | MUC5AC | mucin 5AC, oligomeric mucus/gel-forming | 1.72 | 9.16 x 10^-3^ |
| 224772_at | NAV1 | neuron navigator 1 | 1.51 | 4.60 x 10^-3^ |
| 224774_s_at | NAV1 | neuron navigator 1 | 1.61 | 6.36 x 10^-3^ |
| 229281_at | NPAS3 | neuronal PAS domain protein 3 | -2.11 | 2.87 x 10^-3^ |
| 23939_at | NT5E | 5'-nucleotidase, ecto (CD73) | -1.93 | 8.49 x 10^-4^ |
| 238575_at | OSBPL6 | oxysterol binding protein-like 6 | -1.55 | 7.86 x 10^-4^ |
| 229230_at | OSTalpha | organic solute transporter alpha | 1.55 | 6.59 x 10^-3^ |
| 228863_at | PCDH17 | protocadherin 17 | -2.67 | 1.43 x 10^-3^ |
| 22446_s_at | PDE7A | phosphodiesterase 7A | -1.90 | 1.48 x 10^-3^ |
| 212094_at | PEG10 | paternally expressed 10 | -2.4 | 3.96 x 10^-3^ |
| 217996_at | PHLDA1 | pleckstrin homology-like domain, family A, member 1 | 2.27 | 4.26 x 10^-6^ |
| 217997_at | PHLDA1 | pleckstrin homology-like domain, family A, member 1 | 2.23 | 1.08 x 10^-4^ |
| 225842_at | PHLDA1 | pleckstrin homology-like domain, family A, member 1 | 2.36 | 1.46 x 10^-4^ |
| 23680_at | PRKAR2B | protein kinase, cAMP-dependent, regulatory, type II, beta | -1.74 | 1.63 x 10^-3^ |
| 207808_s_at | PROS1 | protein S (alpha) | -2.36 | 8.64 x 10^-4^ |
| 208300_at | PTPRH | protein tyrosine phosphatase, receptor type, H | 3.46 | 7.21 x 10^-4^ |
| 202148_s_at | PYCR1 | pyrroline-5-carboxylate reductase 1 | 1.53 | 5.00 x 10^-3^ |
| 26884_s_at | SCEL | sciellin | 4.58 | 7.21 x 10^-4^ |
| 228782_at | SCGB3A2 | secretoglobin, family 3A, member 2 | -14.08 | 5.95 x 10^-3^ |
| 217272_s_at | SERPINB13 | serpin peptidase inhibitor, clade B (ovalbumin), member 13 | 1.72 | 1.48 x 10^-3^ |
| 2634_at | SERPINB8 | serpin peptidase inhibitor, clade B (ovalbumin), member 8 | 1.75 | 2.87 x 10^-3^ |
| 33322_i_at | SFN | stratifin | 1.90 | 9.63 x 10^-6^ |
| 33323_r_at | SFN | stratifin | 1.98 | 5.43 x 10^-6^ |
| 237746_at | SFRS11 | Arginine-rich nuclear protein | -1.96 | 9.83 x 10^-3^ |
| 24688_at | SGCE | sarcoglycan, epsilon | -1.92 | 7.86 x 10^-4^ |
| 239435_x_at | SHROOM1 | shroom family member 1 | 1.55 | 8.27 x 10^-4^ |
| 232481_s_at | SLITRK6 | SLIT and NTRK-like family, member 6 | -1.59 | 3.68 x 10^-3^ |
| 235976_at | SLITRK6 | SLIT and NTRK-like family, member 6 | -1.99 | 3.56 x 10^-3^ |
| 1552396_at | SPINLW1 /// WFDC6 | serine peptidase inhibitor-like, with Kunitz and WAP domains 1 (eppin) /// WAP four-disulfide core domain 6 | -1.83 | 6.61 x 10^-3^ |
| 209875_s_at | SPP1 | secreted phosphoprotein 1 | 3.99 | 1.77 x 10^-3^ |
| 2564_at | SPRR1B | small proline-rich protein 1B (cornifin) | 4.4 | 3.68 x 10^-3^ |
| 218990_s_at | SPRR3 | small proline-rich protein 3 | 6.08 | 5.45 x 10^-5^ |
| 25499_at | SRPX2 | sushi-repeat-containing protein, X-linked 2 | 2.75 | 3.81 x 10^-5^ |
| 220187_at | STEAP4 | STEAP family member 4 | -2.72 | 7.15 x 10^-4^ |
| 225987_at | STEAP4 | STEAP family member 4 | -1.66 | 3.73 x 10^-3^ |
| 229991_s_at | SYTL4 | Synaptotagmin-like 4 (SYTL4), transcript variant 2, mRNA | 2.26 | 1.48 x 10^-3^ |
| 25513_at | TCN1 | transcobalamin I (vitamin B12 binding protein, R binder family) | 3.66 | 5.43 x 10^-6^ |
| 201666_at | TIMP1 | TIMP metallopeptidase inhibitor 1 | 1.69 | 6.36 x 10^-3^ |
| 219410_at | TMEM45A | transmembrane protein 45A | -2.50 | 2.30 x 10^-3^ |
| 23961_at | TPRXL | tetra-peptide repeat homeobox-like | 3.39 | 1.46 x 10^-4^ |
| 223694_at | TRIM7 | tripartite motif-containing 7 | 1.88 | 7.21 x 10^-4^ |
| 239694_at | TRIM7 | tripartite motif-containing 7 | 1.63 | 6.36 x 10^-3^ |
| 21065_s_at | UPK1B | uroplakin 1B | 2.02 | 3.97 x 10^-3^ |
| 227399_at | VGLL3 | vestigial like 3 (Drosophila) | -2.25 | 9.16 x 10^-3^ |
| 219478_at | WFDC1 | WAP four-disulfide core domain 1 | 2.09 | 1.48 x 10^-3^ |
| 228850_s_at | --- | --- | -1.77 | 8.07 x 10^-3^ |
| 230130_at | --- | --- | -2.32 | 5.37 x 10^-3^ |
| 23620_at | --- | --- | -1.61 | 6.85 x 10^-4^ |
| 236261_at | --- | --- | -1.62 | 4.41 x 10^-3^ |
| 238755_at | --- | --- | -1.50 | 8.07 x 10^-3^ |

^1^ Data obtained using the Affymetrix HG-U133 Plus 2.0 microarray chip.

^2^ Fold-change represents ratio of average expression value in COPD smokers to average expression value in low responder healthy smokers. Positive fold-changes represent genes more highly expressed in COPD smokers; negative fold-changes represent genes more highly expressed in low responder healthy smokers.

^3^ p value obtained using Benjamini-Hochberg correction to limit the false positive rate.
