# Supplementary material for: Biologic Phenotyping of the Human Small Airway Epithelial Response to Cigarette Smoking
Source: PLoS One. 2011 Jul 28;6(7):e22798. doi: 10.1371/journal.pone.0022798 (PMC3145669; doi:10.1371/journal.pone.0022798)
Supplement: Table S9 — Genes differentially expressed in the small airway epithelium of COPD smokers and high responder smokers vs low responder smokers. (DOC) [file pone.0022798.s012.doc]

| **Probe set ID** | **Gene symbol** | **Gene title** | **Fold-change (COPD smokers/low responder healthy smokers)^2^** | **p value^3^** |
| --- | --- | --- | --- | --- |
|  |  |  |  |  |
| 202912_at | ADM | adrenomedullin | 2.67 | 2.21 x 10^-3^ |
| 229354_at | AHRR /// PDCD6 | aryl-hydrocarbon receptor repressor /// programmed cell death 6 | 2.80 | 2.30 x 10^-3^ |
| 227530_at | AKAP12 | A kinase (PRKA) anchor protein 12 | 2.18 | 8.07 x 10^-3^ |
| 26561_s_at | AKR1B10 | aldo-keto reductase family 1, member B10 (aldose reductase) | 3.74 | 8.07 x 10^-3^ |
| 209369_at | ANXA3 | annexin A3 | 1.58 | 1.31 x 10^-3^ |
| 221161_at | ASCL3 | achaete-scute complex homolog 3 (Drosophila) | 1.88 | 3.88 x 10^-3^ |
| 214070_s_at | ATP10B | ATPase, class V, type 10B | 1.86 | 5.45 x 10^-5^ |
| 208836_at | ATP1B3 | ATPase, Na+/K+ transporting, beta 3 polypeptide | 1.56 | 4.37 x 10^-3^ |
| 231126_at | C2orf70 | chromosome 2 open reading frame 70 | 1.52 | 6.17 x 10^-3^ |
| 216598_s_at | CCL2 | chemokine (C-C motif) ligand 2 | 5.98 | 1.23 x 10^-3^ |
| 226545_at | CD109 | CD109 molecule | 1.96 | 5.45 x 10^-5^ |
| 229900_at | CD109 | CD109 molecule | 2.62 | 1.93 x 10^-4^ |
| 208653_s_at | CD164 | CD164 molecule, sialomucin | -1.61 | 8.27 x 10^-3^ |
| 21263_at | CD44 | CD44 molecule (Indian blood group) | 1.64 | 2.39 x 10^-3^ |
| 201884_at | CEACAM5 | carcinoembryonic antigen-related cell adhesion molecule 5 | 3.10 | 1.43 x 10^-6^ |
| 202712_s_at | CKMT1A /// CKMT1B /// LOC100133623 | creatine kinase, mitochondrial 1A /// creatine kinase, mitochondrial 1B /// similar to Creatine kinase, ubiquitous mitochondrial precursor (U-MtCK) (Mia-CK) (Acidic-type mitochondrial creatine kinase) | 1.57 | 2.39 x 10^-3^ |
| 26164_at | CLCA2 | chloride channel regulator 2 | -1.88 | 3.11 x 10^-3^ |
| 25328_at | CLDN10 | claudin 10 | 1.76 | 1.43 x 10^-3^ |
| 22332_at | CLDN16 | claudin 16 | -1.88 | 8.58 x 10^-3^ |
| 219890_at | CLEC5A | C-type lectin domain family 5, member A | 4.33 | 8.27 x 10^-4^ |
| 24971_at | CSTA | cystatin A (stefin A) | 1.82 | 1.48 x 10^-3^ |
| 218002_s_at | CXCL14 | chemokine (C-X-C motif) ligand 14 | 7.70 | 2.47 x 10^-5^ |
| 222484_s_at | CXCL14 | chemokine (C-X-C motif) ligand 14 | 6.67 | 1.48 x 10^-3^ |
| 25749_at | CYP1A1 | cytochrome P450, family 1, subfamily A, polypeptide 1 | 11.58 | 4.08 x 10^-3^ |
| 202435_s_at | CYP1B1 | cytochrome P450, family 1, subfamily B, polypeptide 1 | 4.52 | 1.86 x 10^-3^ |
| 202436_s_at | CYP1B1 | cytochrome P450, family 1, subfamily B, polypeptide 1 | 4.87 | 1.27 x 10^-3^ |
| 202437_s_at | CYP1B1 | cytochrome P450, family 1, subfamily B, polypeptide 1 | 4.09 | 6.75 x 10^-3^ |
| 21397_at | DEFB1 | defensin, beta 1 | 2.11 | 6.36 x 10^-3^ |
| 24687_at | DKFZP564O0823 | DKFZP564O0823 protein | 1.72 | 9.09 x 10^-3^ |
| 226863_at | FAM110C | family with sequence similarity 110, member C | 1.60 | 1.79 x 10^-3^ |
| 227194_at | FAM3B | family with sequence similarity 3, member B | 2.15 | 5.45 x 10^-5^ |
| 210889_s_at | FCGR2B | Fc fragment of IgG, low affinity IIb, receptor (CD32) | 3.42 | 3.22 x 10^-3^ |
| 25014_at | FGFBP1 | fibroblast growth factor binding protein 1 | 2.6 | 9.28 x 10^-4^ |
| 218980_at | FHOD3 | formin homology 2 domain containing 3 | -2.20 | 2.21 x 10^-3^ |
| 25278_at | GAD1 | glutamate decarboxylase 1 (brain, 67kDa) | 2.67 | 2.78 x 10^-3^ |
| 26669_at | GAD1 | glutamate decarboxylase 1 (brain, 67kDa) | 2.27 | 8.41 x 10^-3^ |
| 217755_at | HN1 | hematological and neurological expressed 1 | 1.52 | 4.20 x 10^-3^ |
| 230849_at | KCNA1 | potassium voltage-gated channel, shaker-related subfamily, member 1 (episodic ataxia with myokymia) | -2.36 | 8.41 x 10^-3^ |
| 209125_at | KRT6A | keratin 6A | 5.12 | 1.46 x 10^-4^ |
| 1557207_s_at | LOC283177 | hypothetical protein LOC283177 | -2.00 | 1.48 x 10^-3^ |
| 202018_s_at | LTF | lactotransferrin | -3.64 | 8.07 x 10^-3^ |
| 202291_s_at | MGP | matrix Gla protein | -2.88 | 8.07 x 10^-3^ |
| 214385_s_at | MUC5AC | mucin 5AC, oligomeric mucus/gel-forming | 1.72 | 9.16 x 10^-3^ |
| 224772_at | NAV1 | neuron navigator 1 | 1.51 | 4.60 x 10^-3^ |
| 224774_s_at | NAV1 | neuron navigator 1 | 1.61 | 6.36 x 10^-3^ |
| 229281_at | NPAS3 | neuronal PAS domain protein 3 | -2.11 | 2.87 x 10^-3^ |
| 23939_at | NT5E | 5'-nucleotidase, ecto (CD73) | -1.93 | 8.49 x 10^-4^ |
| 238575_at | OSBPL6 | oxysterol binding protein-like 6 | -1.55 | 7.86 x 10^-4^ |
| 229230_at | OSTalpha | organic solute transporter alpha | 1.55 | 6.59 x 10^-3^ |
| 228863_at | PCDH17 | protocadherin 17 | -2.67 | 1.43 x 10^-3^ |
| 22446_s_at | PDE7A | phosphodiesterase 7A | -1.90 | 1.48 x 10^-3^ |
| 212094_at | PEG10 | paternally expressed 10 | -2.4 | 3.96 x 10^-3^ |
| 217996_at | PHLDA1 | pleckstrin homology-like domain, family A, member 1 | 2.27 | 4.26 x 10^-6^ |
| 217997_at | PHLDA1 | pleckstrin homology-like domain, family A, member 1 | 2.23 | 1.08 x 10^-4^ |
| 225842_at | PHLDA1 | pleckstrin homology-like domain, family A, member 1 | 2.36 | 1.46 x 10^-4^ |
| 23680_at | PRKAR2B | protein kinase, cAMP-dependent, regulatory, type II, beta | -1.74 | 1.63 x 10^-3^ |
| 207808_s_at | PROS1 | protein S (alpha) | -2.36 | 8.64 x 10^-4^ |
| 208300_at | PTPRH | protein tyrosine phosphatase, receptor type, H | 3.46 | 7.21 x 10^-4^ |
| 202148_s_at | PYCR1 | pyrroline-5-carboxylate reductase 1 | 1.53 | 5.00 x 10^-3^ |
| 26884_s_at | SCEL | sciellin | 4.58 | 7.21 x 10^-4^ |
| 228782_at | SCGB3A2 | secretoglobin, family 3A, member 2 | -14.08 | 5.95 x 10^-3^ |
| 217272_s_at | SERPINB13 | serpin peptidase inhibitor, clade B (ovalbumin), member 13 | 1.72 | 1.48 x 10^-3^ |
| 2634_at | SERPINB8 | serpin peptidase inhibitor, clade B (ovalbumin), member 8 | 1.75 | 2.87 x 10^-3^ |
| 33322_i_at | SFN | stratifin | 1.90 | 9.63 x 10^-6^ |
| 33323_r_at | SFN | stratifin | 1.98 | 5.43 x 10^-6^ |
| 237746_at | SFRS11 | Arginine-rich nuclear protein | -1.96 | 9.83 x 10^-3^ |
| 24688_at | SGCE | sarcoglycan, epsilon | -1.92 | 7.86 x 10^-4^ |
| 239435_x_at | SHROOM1 | shroom family member 1 | 1.55 | 8.27 x 10^-4^ |
| 232481_s_at | SLITRK6 | SLIT and NTRK-like family, member 6 | -1.59 | 3.68 x 10^-3^ |
| 235976_at | SLITRK6 | SLIT and NTRK-like family, member 6 | -1.99 | 3.56 x 10^-3^ |
| 1552396_at | SPINLW1 /// WFDC6 | serine peptidase inhibitor-like, with Kunitz and WAP domains 1 (eppin) /// WAP four-disulfide core domain 6 | -1.83 | 6.61 x 10^-3^ |
| 209875_s_at | SPP1 | secreted phosphoprotein 1 | 3.99 | 1.77 x 10^-3^ |
| 2564_at | SPRR1B | small proline-rich protein 1B (cornifin) | 4.4 | 3.68 x 10^-3^ |
| 218990_s_at | SPRR3 | small proline-rich protein 3 | 6.08 | 5.45 x 10^-5^ |
| 25499_at | SRPX2 | sushi-repeat-containing protein, X-linked 2 | 2.75 | 3.81 x 10^-5^ |
| 220187_at | STEAP4 | STEAP family member 4 | -2.72 | 7.15 x 10^-4^ |
| 225987_at | STEAP4 | STEAP family member 4 | -1.66 | 3.73 x 10^-3^ |
| 229991_s_at | SYTL4 | Synaptotagmin-like 4 (SYTL4), transcript variant 2, mRNA | 2.26 | 1.48 x 10^-3^ |
| 25513_at | TCN1 | transcobalamin I (vitamin B12 binding protein, R binder family) | 3.66 | 5.43 x 10^-6^ |
| 201666_at | TIMP1 | TIMP metallopeptidase inhibitor 1 | 1.69 | 6.36 x 10^-3^ |
| 219410_at | TMEM45A | transmembrane protein 45A | -2.50 | 2.30 x 10^-3^ |
| 23961_at | TPRXL | tetra-peptide repeat homeobox-like | 3.39 | 1.46 x 10^-4^ |
| 223694_at | TRIM7 | tripartite motif-containing 7 | 1.88 | 7.21 x 10^-4^ |
| 239694_at | TRIM7 | tripartite motif-containing 7 | 1.63 | 6.36 x 10^-3^ |
| 21065_s_at | UPK1B | uroplakin 1B | 2.02 | 3.97 x 10^-3^ |
| 227399_at | VGLL3 | vestigial like 3 (Drosophila) | -2.25 | 9.16 x 10^-3^ |
| 219478_at | WFDC1 | WAP four-disulfide core domain 1 | 2.09 | 1.48 x 10^-3^ |
| 228850_s_at | --- | --- | -1.77 | 8.07 x 10^-3^ |
| 230130_at | --- | --- | -2.32 | 5.37 x 10^-3^ |
| 23620_at | --- | --- | -1.61 | 6.85 x 10^-4^ |
| 236261_at | --- | --- | -1.62 | 4.41 x 10^-3^ |
| 238755_at | --- | --- | -1.50 | 8.07 x 10^-3^ |

^1^ Data obtained using the Affymetrix HG-U133 Plus 2.0 microarray chip.

^2^ Fold-change represents ratio of average expression value in COPD smokers to average expression value in low responder healthy smokers. Positive fold-changes represent genes more highly expressed in COPD smokers; negative fold-changes represent genes more highly expressed in low responder healthy smokers.

^3^ p value obtained using Benjamini-Hochberg correction to limit the false positive rate.

| **Probe set ID** | **Gene symbol** | **Gene title** | | **Fold-change (COPD & high responder/low responder)^2^** | **p value^3^** | **P call (%)^4^** |
| --- | --- | --- | --- | --- | --- | --- |
| 1554878_a_at | ABCD3 | ATP-binding cassette, sub-family D (ALD), member 3 | | 1.60 | 1.81x10^-03^ | 100 |
| 214895_s_at | ADAM10 | ADAM metallopeptidase domain 10 | | 1.55 | 2.30x10^-03^ | 96 |
| 202912_at | ADM | adrenomedullin | | -1.75 | 5.78x10^-03^ | 70 |
| 226955_at | AFAP1L1 | hypothetical protein FLJ36748 | | 1.61 | 2.17x10^-03^ | 88 |
| 243937_x_at | AGAP10 /// AGAP4 /// AGAP9 /// BMS1P1 /// BMS1P5 /// LOC399753 | hypothetical gene supported by AK093334; AL833330; BC020871; BC032492 ; centaurin, gamma-like family, member 2 | | 1.60 | 5.86x10^-03^ | 76 |
| 229354_at | AHRR | programmed cell death 6 ; aryl-hydrocarbon receptor repressor | | -2.46 | 1.38x10^-03^ | 81 |
| 206561_s_at | AKR1B10 | aldo-keto reductase family 1, member B10 (aldose reductase) | | -3.68 | 1.14x10^-03^ | 99 |
| 208498_s_at | AMY1A /// AMY1B /// AMY1C /// AMY2A /// AMY2B | amylase, alpha 1A; salivary ; amylase, alpha 1B; salivary ; amylase, alpha 1C; salivary ; amylase, alpha 2A; pancreatic ; amylase, alpha 2B; pancreatic | | 1.59 | 2.06x10^-04^ | 100 |
| 1555731_a_at | AP1S3 | adaptor-related protein complex 1, sigma 3 subunit | | 1.62 | 3.97x10^-03^ | 90 |
| 1552627_a_at | ARHGAP5 | Rho GTPase activating protein 5 | | 1.55 | 1.38x10^-03^ | 95 |
| 230141_at | ARID4A | AT rich interactive domain 4A (RBP1-like) | | 1.56 | 5.76x10^-04^ | 96 |
| 221161_at | ASCL3 | achaete-scute complex (Drosophila) homolog-like 3 | | -1.58 | 7.88x10^-03^ | 81 |
| 205446_s_at | ATF2 | activating transcription factor 2 | | 1.53 | 2.27x10^-03^ | 96 |
| 237968_at | ATL2 | ADP-ribosylation factor-like 6 interacting protein 2 | | 1.70 | 7.24x10^-04^ | 65 |
| 214070_s_at | ATP10B | ATPase, Class V, type 10B | | -1.54 | 2.21x10^-04^ | 80 |
| 214575_s_at | AZU1 | azurocidin 1 (cationic antimicrobial protein 37) | | 1.75 | 1.02x10^-03^ | 20 |
| 238924_at | BMS1P1 /// BMS1P5 | Hypothetical protein LOC255326 | | 1.78 | 3.48x10^-03^ | 90 |
| 221003_s_at | CAB39L | calcium binding protein 39-like ; calcium binding protein 39-like | | 2.04 | 2.25x10^-03^ | 29 |
| 219928_s_at | CABYR | calcium binding tyrosine-(Y)-phosphorylation regulated (fibrousheathin 2) | | -2.05 | 6.59x10^-03^ | 100 |
| 224279_s_at | CABYR | calcium binding tyrosine-(Y)-phosphorylation regulated (fibrousheathin 2) | | -2.15 | 7.40x10^-03^ | 93 |
| 204811_s_at | CACNA2D2 | calcium channel, voltage-dependent, alpha 2/delta subunit 2 | | -1.50 | 8.89x10^-03^ | 30 |
| 1560458_s_at | CAPS2 | calcyphosine 2 | | 1.53 | 3.21x10^-03^ | 99 |
| 206788_s_at | CBFB | core-binding factor, beta subunit | | 1.56 | 2.40x10^-04^ | 88 |
| 216598_s_at | CCL2 | chemokine (C-C motif) ligand 2 | | -3.60 | 3.63x10^-03^ | 58 |
| 214638_s_at | CCNT2 | cyclin T2 |  | 1.50 | 3.04x10^-03^ | 59 |

| **Probe set ID** | **Gene symbol** | **Gene title** | | **Fold-change (COPD & high responder/low responder)** | **p value** | **P call (%)** |
| --- | --- | --- | --- | --- | --- | --- |
| 1552612_at | CDC42SE2 | CDC42 small effector 2 | | 1.56 | 9.28x10^-03^ | 96 |
| 1552613_s_at | CDC42SE2 | CDC42 small effector 2 | | 1.52 | 5.92x10^-03^ | 90 |
| 216894_x_at | CDKN1C | cyclin-dependent kinase inhibitor 1C (p57, Kip2) | | 1.76 | 3.06x10^-03^ | 40 |
| 201884_at | CEACAM5 | carcinoembryonic antigen-related cell adhesion molecule 5 | | -2.10 | 5.59x10^-04^ | 100 |
| 1555564_a_at | CFI | I factor (complement) | | 1.62 | 5.77x10^-03^ | 93 |
| 206164_at | CLCA2 | chloride channel, calcium activated, family member 2 | | 1.86 | 5.20x10^-04^ | 100 |
| 206165_s_at | CLCA2 | chloride channel, calcium activated, family member 2 | | 1.88 | 2.67x10^-04^ | 100 |
| 206166_s_at | CLCA2 | chloride channel, calcium activated, family member 2 | | 1.88 | 1.29x10^-03^ | 100 |
| 217528_at | CLCA2 | chloride channel, calcium activated, family member 2 | | 1.71 | 3.28x10^-03^ | 100 |
| 220332_at | CLDN16 | claudin 16 | | 1.84 | 2.06x10^-04^ | 60 |
| 214598_at | CLDN8 | claudin 8 |  | 1.58 | 4.37x10^-03^ | 100 |
| 219890_at | CLEC5A | C-type lectin domain family 5, member A | | -3.28 | 9.15x10^-04^ | 23 |
| 1568836_at | CLK4 | CDC-like kinase 4 | | 1.83 | 4.75x10^-03^ | 83 |
| 210769_at | CNGB1 | cyclic nucleotide gated channel beta 1 | | -1.57 | 7.61x10^-03^ | 75 |
| 201445_at | CNN3 | calponin 3, acidic | | 1.72 | 1.21x10^-03^ | 100 |
| 201117_s_at | CPE | carboxypeptidase E | | -1.72 | 5.92x10^-03^ | 98 |
| 1555106_a_at | CTDSPL2 | CTD (carboxy-terminal domain, RNA polymerase II, polypeptide A) small phosphatase like 2 | | 1.55 | 3.69x10^-03^ | 86 |
| 1555716_a_at | CXADR | coxsackie virus and adenovirus receptor | | 1.54 | 3.94x10^-04^ | 93 |
| 218002_s_at | CXCL14 | chemokine (C-X-C motif) ligand 14 | | -5.13 | 4.61x10^-05^ | 34 |
| 222484_s_at | CXCL14 | chemokine (C-X-C motif) ligand 14 | | -3.77 | 2.52x10^-03^ | 41 |
| 222453_at | CYBRD1 | cytochrome b reductase 1 | | 1.56 | 4.39x10^-03^ | 100 |
| 205749_at | CYP1A1 | cytochrome P450, family 1, subfamily A, polypeptide 1 | | -8.13 | 1.45x10^-03^ | 58 |
| 202435_s_at | CYP1B1 | cytochrome P450, family 1, subfamily B, polypeptide 1 | | -3.53 | 1.88x10^-03^ | 98 |
| 202436_s_at | CYP1B1 | cytochrome P450, family 1, subfamily B, polypeptide 1 | | -3.69 | 1.63x10^-03^ | 99 |
| 202437_s_at | CYP1B1 | cytochrome P450, family 1, subfamily B, polypeptide 1 | | -3.26 | 4.96x10^-03^ | 99 |
| 206515_at | CYP4F3 | cytochrome P450, family 4, subfamily F, polypeptide 3 | | -2.18 | 2.23x10^-03^ | 98 |
| 227702_at | CYP4X1 | cytochrome P450, family 4, subfamily X, polypeptide 1 | | 1.55 | 2.06x10^-04^ | 100 |
| 216607_s_at | CYP51A1 | cytochrome P450, family 51, subfamily A, polypeptide 1 | | 1.65 | 2.23x10^-03^ | 100 |
| 222678_s_at | DCUN1D1 | DCN1, defective in cullin neddylation 1, domain containing 1 (S. cerevisiae) | | 1.71 | 3.20x10^-03^ | 65 |
| 1553565_s_at | DDAH1 | dimethylarginine dimethylaminohydrolase 1 | | 2.05 | 3.60x10^-03^ | 39 |
| 210397_at | DEFB1 | defensin, beta 1 | | -1.69 | 8.94x10^-03^ | 99 |
| 203716_s_at | DPP4 | dipeptidylpeptidase 4 (CD26, adenosine deaminase complexing protein 2) | | 2.79 | 7.03x10^-04^ | 21 |
| 203717_at | DPP4 | dipeptidylpeptidase 4 (CD26, adenosine deaminase complexing protein 2) | | 1.72 | 3.23x10^-04^ | 89 |
| 211478_s_at | DPP4 | dipeptidylpeptidase 4 (CD26, adenosine deaminase complexing protein 2) | | 2.22 | 2.71x10^-04^ | 76 |
| 214652_at | DRD1 | dopamine receptor D1 | | 2.25 | 4.96x10^-03^ | 36 |
| 219727_at | DUOX2 | dual oxidase 2 | | -2.33 | 8.06x10^-03^ | 89 |
| 222932_at | EHF | ets homologous factor | | 1.51 | 4.30x10^-03^ | 98 |
| 1555996_s_at | EIF4A2 | eukaryotic translation initiation factor 4A, isoform 2 | | 1.56 | 5.24x10^-03^ | 99 |
| 220120_s_at | EPB41L4A | erythrocyte membrane protein band 4.1 like 4A | | 1.54 | 7.27x10^-03^ | 68 |
| 396_f_at | EPOR | erythropoietin receptor | | 1.52 | 7.14x10^-03^ | 93 |
| 227450_at | ERP27 | hypothetical protein FLJ32115 | | 1.62 | 4.27x10^-03^ | 99 |
| 226432_at | ETNK1 | MRNA; cDNA DKFZp566C034 (from clone DKFZp566C034) | | 1.53 | 2.04x10^-04^ | 100 |
| 231576_at | ETNK1 | MRNA; cDNA DKFZp566C034 (from clone DKFZp566C034) | | 1.82 | 3.64x10^-04^ | 100 |
| 219432_at | EVC | Ellis van Creveld syndrome | | 1.78 | 1.51x10^-03^ | 31 |
| 220285_at | FAM108B1 | chromosome 9 open reading frame 77 | | 1.61 | 3.23x10^-04^ | 100 |
| 227194_at | FAM3B | family with sequence similarity 3, member B | | -1.60 | 2.20x10^-03^ | 99 |
| 220306_at | FAM46C | family with sequence similarity 46, member C | | 1.66 | 3.34x10^-04^ | 100 |
| 226811_at | FAM46C | family with sequence similarity 46, member C | | 1.50 | 7.77x10^-04^ | 100 |
| 222013_x_at | FAM86A | amyloid beta (A4) precursor protein (peptidase nexin-II, Alzheimer disease) | | 1.54 | 2.28x10^-03^ | 28 |
| 205014_at | FGFBP1 | fibroblast growth factor binding protein 1 | | -1.53 | 3.65x10^-03^ | 75 |
| 203639_s_at | FGFR2 | fibroblast growth factor receptor 2 (bacteria-expressed kinase, keratinocyte growth factor receptor, craniofacial dysostosis 1, Crouzon syndrome, Pfeiffer syndrome, Jackson-Weiss syndrome) | | 1.57 | 6.47x10^-03^ | 94 |
| 218980_at | FHOD3 | formin homology 2 domain containing 3 | | 1.83 | 7.06x10^-04^ | 99 |
| 228268_at | FMO2 | flavin containing monooxygenase 2 | | 1.52 | 9.83x10^-03^ | 100 |
| 215910_s_at | FNDC3A | fibronectin type III domain containing 3A | | 1.59 | 1.40x10^-03^ | 83 |
| 204437_s_at | FOLR1 | folate receptor 1 (adult) | | 1.72 | 1.94x10^-04^ | 100 |
| 225163_at | FRMD4A | FERM domain containing 4A | | 1.51 | 3.23x10^-03^ | 89 |
| 206669_at | GAD1 | glutamate decarboxylase 1 (brain, 67kDa) | | -2.17 | 3.04x10^-03^ | 38 |
| 211810_s_at | GALC | galactosylceramidase (Krabbe disease) | | 1.53 | 6.85x10^-03^ | 98 |
| 1555125_at | GCFC1 | chromosome 21 open reading frame 66 | | 1.58 | 4.33x10^-03^ | 84 |
| 223079_s_at | GLS | glutaminase | | 1.55 | 2.21x10^-03^ | 85 |
| 227214_at | GOPC | golgi associated PDZ and coiled-coil motif containing | | 1.66 | 7.00x10^-04^ | 85 |
| 212950_at | GPR116 | G protein-coupled receptor 116 | | 1.51 | 5.47x10^-03^ | 100 |
| 1555122_at | GPR125 | G protein-coupled receptor 125 | | 1.55 | 7.94x10^-03^ | 59 |
| 239595_at | GPX2 | Glutathione peroxidase 2 (gastrointestinal) | | -1.63 | 7.95x10^-03^ | 95 |
| 242336_at | GSK3B | Glycogen synthase kinase 3 beta | | 2.21 | 4.51x10^-04^ | 50 |
| 231955_s_at | HIBADH | 3-hydroxyisobutyrate dehydrogenase | | 1.50 | 5.92x10^-03^ | 100 |
| 214455_at | HIST1H2BC | histone 1, H2bc | | 1.53 | 4.27x10^-03^ | 100 |
| 215779_s_at | HIST1H2BG | histone 1, H2bg | | 1.54 | 4.89x10^-03^ | 84 |
| 214616_at | HIST1H3E | histone 1, H3e | | 1.53 | 3.25x10^-03^ | 55 |
| 214290_s_at | HIST2H2AA3 /// HIST2H2AA4 | histone 2, H2aa | | 1.51 | 1.72x10^-03^ | 100 |
| 228772_at | HNMT | histamine N-methyltransferase | | 1.55 | 4.96x10^-03^ | 99 |
| 219985_at | HS3ST3A1 | heparan sulfate (glucosamine) 3-O-sulfotransferase 3A1 | | -2.98 | 1.71x10^-03^ | 26 |
| 214059_at | IFI44 | Interferon-induced protein 44 | | 1.74 | 3.04x10^-03^ | 86 |
| 236478_at | IFNAR1 | Transcribed locus | | 1.52 | 4.08x10^-03^ | 53 |
| 243358_at | IGF1R | hypothetical protein MGC18216 | | 1.96 | 6.17x10^-03^ | 63 |
| 202948_at | IL1R1 | interleukin 1 receptor, type I | | 1.51 | 1.73x10^-03^ | 100 |
| 210744_s_at | IL5RA | interleukin 5 receptor, alpha | | 1.54 | 6.89x10^-04^ | 96 |
| 204864_s_at | IL6ST | interleukin 6 signal transducer (gp130, oncostatin M receptor) | | 1.98 | 1.12x10^-03^ | 86 |
| 223597_at | ITLN1 | intelectin 1 (galactofuranose binding) | | 2.76 | 4.37x10^-03^ | 28 |
| 223585_x_at | KBTBD2 | kelch repeat and BTB (POZ) domain containing 2 | | 1.50 | 4.34x10^-03^ | 91 |
| 230849_at | KCNA1 | Potassium voltage-gated channel, shaker-related subfamily, member 1 (episodic ataxia with myokymia) | | 2.04 | 2.32x10^-04^ | 89 |
| 238001_at | KCTD6 | potassium channel tetramerisation domain containing 6 | | 1.63 | 3.37x10^-03^ | 68 |
| 242098_at | KIAA1244 | hypothetical protein LOC202451 | | 1.72 | 1.06x10^-03^ | 93 |
| 231869_at | KIAA1586 | KIAA1586 |  | 1.61 | 9.71x10^-03^ | 38 |
| 221986_s_at | KLHL24 | kelch-like 24 (Drosophila) | | 1.52 | 3.32x10^-03^ | 96 |
| 242088_at | KLHL24 | kelch-like 24 (Drosophila) | | 1.70 | 9.00x10^-04^ | 80 |
| 206241_at | KPNA5 | karyopherin alpha 5 (importin alpha 6) | | 1.65 | 2.21x10^-04^ | 100 |
| 209125_at | KRT6A | keratin 6A ; keratin 6C ; keratin 6E | | -2.51 | 8.25x10^-03^ | 28 |
| 219399_at | LIN7C | lin-7 homolog C (C. elegans) | | 1.55 | 1.30x10^-03^ | 99 |
| 1565936_a_at | LMO3 | LIM domain only 3 (rhombotin-like 2) | | 1.55 | 5.38x10^-03^ | 78 |
| 1558308_at | LOC100292680 | hypothetical gene supported by AK090616 | | -2.25 | 7.40x10^-03^ | 50 |
| 214385_s_at | LOC100293983 /// MUC5AC | mucin 5, subtypes A and C, tracheobronchial/gastric | | -1.60 | 3.13x10^-03^ | 100 |
| 1557207_s_at | LOC283177 | hypothetical protein LOC283177 | | 1.68 | 3.13x10^-03^ | 95 |
| 1553674_at | LRRIQ3 | leucine rich repeat containing 44 | | 1.57 | 3.23x10^-04^ | 99 |
| 202018_s_at | LTF | lactotransferrin | | 2.67 | 1.53x10^-03^ | 81 |
| 235106_at | MAML2 | mastermind-like 2 (Drosophila) | | 1.53 | 9.27x10^-04^ | 98 |
| 204041_at | MAOB | monoamine oxidase B | | 1.86 | 5.63x10^-04^ | 99 |
| 214786_at | MAP3K1 | mitogen-activated protein kinase kinase kinase 1 | | 1.68 | 5.91x10^-03^ | 61 |
| 215512_at | MARCH6 | membrane-associated ring finger (C3HC4) 6 | | 1.81 | 6.73x10^-04^ | 90 |
| 1555594_a_at | MBNL1 | muscleblind-like (Drosophila) | | 1.67 | 8.66x10^-03^ | 83 |
| 205017_s_at | MBNL2 | muscleblind-like 2 (Drosophila) | | 1.76 | 1.25x10^-03^ | 93 |
| 205018_s_at | MBNL2 | muscleblind-like 2 (Drosophila) | | 1.70 | 5.98x10^-04^ | 99 |
| 211599_x_at | MET | met proto-oncogene (hepatocyte growth factor receptor) ; met proto-oncogene (hepatocyte growth factor receptor) | | 1.51 | 7.08x10^-03^ | 100 |
| 1552312_a_at | MFAP3 | microfibrillar-associated protein 3 | | 1.71 | 2.77x10^-03^ | 78 |
| 202291_s_at | MGP | matrix Gla protein | | 2.51 | 3.48x10^-04^ | 89 |
| 225997_at | MOBKL1A | MOB1, Mps One Binder kinase activator-like 1A (yeast) | | 1.53 | 6.89x10^-04^ | 100 |
| 238451_at | MPP7 | membrane protein, palmitoylated 7 (MAGUK p55 subfamily member 7) | | 1.86 | 2.26x10^-04^ | 100 |
| 243219_x_at | MRPL50 | Mitochondrial ribosomal protein L50 | | 1.64 | 5.06x10^-03^ | 63 |
| 214303_x_at | MUC5AC | mucin 5, subtypes A and C, tracheobronchial/gastric | | -1.71 | 4.54x10^-03^ | 100 |
| 213432_at | MUC5B | mucin 5, subtype B, tracheobronchial | | 1.83 | 7.40x10^-03^ | 100 |
| 1553602_at | MUCL1 | small breast epithelial mucin | | -1.90 | 6.45x10^-03^ | 100 |
| 211139_s_at | NAB1 | NGFI-A binding protein 1 (EGR1 binding protein 1) | | 1.50 | 1.10x10^-04^ | 100 |
| 1552658_a_at | NAV3 | neuron navigator 3 | | 2.04 | 1.67x10^-03^ | 35 |
| 205732_s_at | NCOA2 | nuclear receptor coactivator 2 | | 1.52 | 5.24x10^-03^ | 99 |
| 1553194_at | NEGR1 | neuronal growth regulator 1 | | 1.85 | 7.71x10^-03^ | 50 |
| 234299_s_at | NIN | ninein (GSK3B interacting protein) | | 1.54 | 2.05x10^-03^ | 94 |
| 229281_at | NPAS3 | Transcribed locus, weakly similar to XP_472593.1 OSJNBa0006B20.16 [Oryza sativa (japonica cultivar-group)] | | 1.93 | 2.08x10^-04^ | 84 |
| 230412_at | NPAS3 | Neuronal PAS domain protein 3 | | 1.76 | 3.48x10^-03^ | 59 |
| 1558775_s_at | NSMAF | neutral sphingomyelinase (N-SMase) activation associated factor | | 1.75 | 1.77x10^-03^ | 68 |
| 1553995_a_at | NT5E | 5'-nucleotidase, ecto (CD73) | | 2.08 | 2.89x10^-04^ | 35 |
| 203939_at | NT5E | 5'-nucleotidase, ecto (CD73) | | 1.85 | 6.14x10^-05^ | 100 |
| 224582_s_at | NUCKS1 | Nuclear casein kinase and cyclin-dependent kinase substrate 1 | | 1.68 | 9.00x10^-04^ | 100 |
| 209629_s_at | NXT2 | nuclear transport factor 2-like export factor 2 | | 1.64 | 8.29x10^-04^ | 96 |
| 207564_x_at | OGT | O-linked N-acetylglucosamine (GlcNAc) transferase (UDP-N-acetylglucosamine:polypeptide-N-acetylglucosaminyl transferase) | | 1.51 | 8.56x10^-03^ | 99 |
| 223879_s_at | OXR1 | oxidation resistance 1 | | 1.71 | 9.00x10^-04^ | 100 |
| 214401_at | PAX1 | paired box gene 1 | | 1.68 | 7.14x10^-03^ | 21 |
| 228863_at | PCDH17 | Protocadherin 17 | | 2.05 | 2.29x10^-03^ | 84 |
| 232054_at | PCDH20 | protocadherin 20 | | 1.75 | 1.37x10^-04^ | 98 |
| 235331_x_at | PCGF5 | polycomb group ring finger 5 | | 1.50 | 4.65x10^-03^ | 98 |
| 202731_at | PDCD4 | programmed cell death 4 (neoplastic transformation inhibitor) | | 1.54 | 2.29x10^-03^ | 100 |
| 224046_s_at | PDE7A | phosphodiesterase 7A | | 1.80 | 4.18x10^-05^ | 96 |
| 205960_at | PDK4 | pyruvate dehydrogenase kinase, isoenzyme 4 | | 1.51 | 7.70x10^-03^ | 88 |
| 212092_at | PEG10 | paternally expressed 10 | | 1.63 | 7.74x10^-03^ | 31 |
| 212094_at | PEG10 | paternally expressed 10 | | 1.77 | 3.94x10^-04^ | 91 |
| 1555131_a_at | PER3 | period homolog 3 (Drosophila) | | 1.60 | 3.94x10^-04^ | 53 |
| 201120_s_at | PGRMC1 | progesterone receptor membrane component 1 | | 1.71 | 2.06x10^-04^ | 100 |
| 217996_at | PHLDA1 | pleckstrin homology-like domain, family A, member 1 | | -1.82 | 4.18 x10^-05^ | 99 |
| 225842_at | PHLDA1 | CDNA clone IMAGE:5531727 | | -1.73 | 5.24x10^-03^ | 38 |
| 231120_x_at | PKIB | protein kinase (cAMP-dependent, catalytic) inhibitor beta | | 1.64 | 3.13x10^-03^ | 100 |
| 222699_s_at | PLEKHF2 | pleckstrin homology domain containing, family F (with FYVE domain) member 2 | | 1.56 | 4.77x10^-04^ | 100 |
| 209598_at | PNMA2 | paraneoplastic antigen MA2 | | 2.76 | 4.07x10^-03^ | 69 |
| 222406_s_at | PNRC2 | proline-rich nuclear receptor coactivator 2 | | 1.57 | 1.39x10^-03^ | 95 |
| 1569675_at | POU2AF1 | Homo sapiens, clone IMAGE:4694422, mRNA | | 1.51 | 5.81x10^-03^ | 100 |
| 1552670_a_at | PPP1R3B | hypothetical protein LOC286044 | | 1.65 | 3.71x10^-04^ | 76 |
| 231966_at | PPP1R9A | protein phosphatase 1, regulatory (inhibitor) subunit 9A | | 1.56 | 2.54x10^-03^ | 43 |
| 235502_at | PPP2CA | Protein phosphatase 2 (formerly 2A), catalytic subunit, alpha isoform | | 1.52 | 6.71x10^-03^ | 75 |
| 200604_s_at | PRKAR1A | protein kinase, cAMP-dependent, regulatory, type I, alpha (tissue specific extinguisher 1) | | 1.59 | 4.73x10^-03^ | 100 |
| 203680_at | PRKAR2B | protein kinase, cAMP-dependent, regulatory, type II, beta | | 1.69 | 2.38x10^-04^ | 100 |
| 207808_s_at | PROS1 | protein S (alpha) | | 1.95 | 3.94x10^-05^ | 100 |
| 1555097_a_at | PTGFR | prostaglandin F receptor (FP) | | 1.95 | 1.80x10^-04^ | 99 |
| 207177_at | PTGFR | prostaglandin F receptor (FP) | | 1.60 | 2.41x10^-03^ | 100 |
| 242458_at | RALGPS2 | Ral GEF with PH domain and SH3 binding motif 2 | | 1.56 | 3.23x10^-04^ | 96 |
| 211540_s_at | RB1 | retinoblastoma 1 (including osteosarcoma) | | 1.81 | 6.81x10^-03^ | 40 |
| 235570_at | RBMS3 | RNA binding motif, single stranded interacting protein | | 1.53 | 2.43x10^-04^ | 96 |
| 236911_at | RIMKLB | Family with sequence similarity 80, member B | | 1.57 | 2.66x10^-03^ | 94 |
| 242870_at | RIMKLB | Family with sequence similarity 80, member B | | 1.78 | 2.88x10^-03^ | 96 |
| 242985_x_at | RNF180 | ring finger protein 180 | | 1.72 | 7.31x10^-04^ | 70 |
| 230700_at | RTN4RL1 | reticulon 4 receptor-like 1 | | 1.68 | 7.89x10^-03^ | 26 |
| 210790_s_at | SAR1A | SAR1 gene homolog A (S. cerevisiae) | | 1.72 | 8.48x10^-03^ | 54 |
| 215064_at | SC5DL | Sterol-C5-desaturase (ERG3 delta-5-desaturase homolog, fungal)-like | | 1.88 | 1.07x10^-03^ | 28 |
| 228782_at | SCGB3A2 | secretoglobin, family 3A, member 2 | | 7.81 | 1.41x10^-03^ | 68 |
| 241436_at | SCNN1G | sodium channel, nonvoltage-gated 1, gamma | | 1.68 | 9.00x10^-04^ | 99 |
| 204344_s_at | SEC23A | Sec23 homolog A (S. cerevisiae) | | 1.55 | 6.04x10^-03^ | 39 |
| 220735_s_at | SENP7 | SUMO1/sentrin specific peptidase 7 | | 1.67 | 9.18x10^-04^ | 93 |
| 235337_at | SERTAD4 | SERTA domain containing 4 | | 1.51 | 2.18x10^-04^ | 100 |
| 33322_i_at | SFN | stratifin |  | -1.53 | 3.23x10^-04^ | 100 |
| 33323_r_at | SFN | stratifin |  | -1.52 | 1.41x10^-03^ | 100 |
| 237746_at | SFRS11 | Splicing factor, arginine/serine-rich 11 | | 1.76 | 2.75x10^-03^ | 78 |
| 210077_s_at | SFRS5 | splicing factor, arginine/serine-rich 5 | | 1.57 | 6.01x10^-03^ | 73 |
| 204688_at | SGCE | sarcoglycan, epsilon | | 1.65 | 3.96x10^-04^ | 85 |
| 243141_at | SGMS2 | hypothetical protein MGC26963 | | 1.56 | 1.69x10^-03^ | 99 |
| 235294_at | SIKE1 |  |  | 1.64 | 7.12x10^-03^ | 26 |
| 205317_s_at | SLC15A2 | solute carrier family 15 (H+/peptide transporter), member 2 | | 1.50 | 8.96x10^-04^ | 100 |
| 203908_at | SLC4A4 | solute carrier family 4, sodium bicarbonate cotransporter, member 4 | | 1.53 | 3.25x10^-04^ | 100 |
| 210738_s_at | SLC4A4 | solute carrier family 4, sodium bicarbonate cotransporter, member 4 | | 2.02 | 4.77x10^-04^ | 94 |
| 237058_x_at | SLC6A13 | solute carrier family 6 (neurotransmitter transporter, GABA), member 13 | | 1.90 | 5.02x10^-03^ | 96 |
| 209921_at | SLC7A11 | solute carrier family 7, (cationic amino acid transporter, y+ system) member 11 | | -2.69 | 9.33x10^-04^ | 96 |
| 217678_at | SLC7A11 | solute carrier family 7, (cationic amino acid transporter, y+ system) member 11 | | -3.11 | 5.94x10^-04^ | 99 |
| 222071_s_at | SLCO4C1 | solute carrier organic anion transporter family, member 4C1 | | 1.52 | 2.35x10^-03^ | 100 |
| 209897_s_at | SLIT2 | slit homolog 2 (Drosophila) | | 1.58 | 7.40x10^-03^ | 100 |
| 232176_at | SLITRK6 | SLIT and NTRK-like family, member 6 | | 1.82 | 1.28x10^-04^ | 100 |
| 232481_s_at | SLITRK6 | SLIT and NTRK-like family, member 6 | | 1.59 | 4.18E-05 | 100 |
| 235976_at | SLITRK6 | SLIT and NTRK-like family, member 6 | | 1.85 | 3.23x10^-04^ | 100 |
| 215294_s_at | SMARCA1 | SWI/SNF related, matrix associated, actin dependent regulator of chromatin, subfamily a, member 1 | | 1.59 | 5.25x10^-04^ | 98 |
| 220369_at | SMEK1 | KIAA2010 |  | 1.63 | 6.11x10^-03^ | 58 |
| 208608_s_at | SNTB1 | syntrophin, beta 1 (dystrophin-associated protein A1, 59kDa, basic component 1) | | 1.84 | 3.82x10^-03^ | 79 |
| 209875_s_at | SPP1 | secreted phosphoprotein 1 (osteopontin, bone sialoprotein I, early T-lymphocyte activation 1) | | -3.50 | 1.07x10^-03^ | 98 |
| 205064_at | SPRR1B | small proline-rich protein 1B (cornifin) | | -2.65 | 3.13x10^-03^ | 26 |
| 200672_x_at | SPTBN1 | spectrin, beta, non-erythrocytic 1 | | 1.56 | 2.54x10^-03^ | 100 |
| 213562_s_at | SQLE | squalene epoxidase | | 1.53 | 8.94x10^-03^ | 100 |
| 205499_at | SRPX2 | sushi-repeat-containing protein, X-linked 2 | | -2.15 | 2.06x10^-04^ | 98 |
| 202817_s_at | SS18 | synovial sarcoma translocation, chromosome 18 | | 1.51 | 5.38x10^-03^ | 100 |
| 220187_at | STEAP4 | STEAP family member 4 | | 2.50 | 8.94E-06 | 96 |
| 225987_at | STEAP4 | STEAP family member 4 | | 1.75 | 3.28E-05 | 100 |
| 221727_at | SUB1 | SUB1 homolog (S. cerevisiae) ; SUB1 homolog (S. cerevisiae) pseudogene 1 | | 1.55 | 1.80x10^-04^ | 100 |
| 212353_at | SULF1 | sulfatase 1 | | 1.64 | 9.64x10^-03^ | 81 |
| 223821_s_at | SUSD4 | sushi domain containing 4 ; YHGM196 | | 1.55 | 7.24x10^-04^ | 94 |
| 221618_s_at | TAF9B | TAF9-like RNA polymerase II, TATA box binding protein (TBP)-associated factor, 31kDa | | 1.58 | 1.36x10^-03^ | 100 |
| 221428_s_at | TBL1XR1 | transducin (beta)-like 1X-linked receptor 1 ; transducin (beta)-like 1X-linked receptor 1 | | 1.56 | 6.59x10^-03^ | 100 |
| 205513_at | TCN1 | transcobalamin I (vitamin B12 binding protein, R binder family) | | -2.38 | 2.06x10^-04^ | 93 |
| 1553322_s_at | TEAD1 | TEA domain family member 1 (SV40 transcriptional enhancer factor) | | 1.69 | 9.14x10^-03^ | 54 |
| 1554493_s_at | THADA | thyroid adenoma associated | | 1.50 | 2.30x10^-03^ | 94 |
| 214920_at | THSD7A | hypothetical protein LOC221981 | | 1.76 | 3.25x10^-03^ | 48 |
| 204426_at | TMED2 | transmembrane emp24 domain trafficking protein 2 | | 1.65 | 7.90x10^-03^ | 100 |
| 204427_s_at | TMED2 | transmembrane emp24 domain trafficking protein 2 | | 1.56 | 8.66x10^-03^ | 100 |
| 229623_at | TMEM150C | Similar to hypothetical protein LOC231503 | | 1.64 | 1.95x10^-04^ | 100 |
| 229302_at | TMEM178 | hypothetical protein MGC33926 | | 1.58 | 1.37x10^-03^ | 100 |
| 219410_at | TMEM45A | transmembrane protein 45A | | 2.36 | 4.61 x10^-05^ | 100 |
| 209754_s_at | TMPO | thymopoietin | | 1.60 | 1.50x10^-03^ | 98 |
| 208097_s_at | TMX1 | thioredoxin domain containing ; thioredoxin domain containing | | 1.58 | 2.13x10^-03^ | 100 |
| 216100_s_at | TOR1AIP1 | torsin A interacting protein 1 | | 1.61 | 2.91x10^-03^ | 94 |
| 210995_s_at | TRIM23 | tripartite motif-containing 23 | | 1.60 | 9.58x10^-03^ | 75 |
| 211602_s_at | TRPC1 | transient receptor potential cation channel, subfamily C, member 1 ; transient receptor potential cation channel, subfamily C, member 1 | | 1.70 | 2.17x10^-03^ | 59 |
| 201387_s_at | UCHL1 | ubiquitin carboxyl-terminal esterase L1 (ubiquitin thiolesterase) | | -2.64 | 4.71x10^-03^ | 94 |
| 222502_s_at | UFM1 | ubiquitin-fold modifier 1 | | 1.72 | 1.61x10^-03^ | 99 |
| 206094_x_at | UGT1A1 /// UGT1A10 /// UGT1A3 /// UGT1A4 /// UGT1A5 /// UGT1A6 /// UGT1A7 /// UGT1A8 /// UGT1A9 | UDP glucuronosyltransferase 1 family, polypeptide A6 | | -1.62 | 3.10x10^-03^ | 100 |
| 215125_s_at | UGT1A1 /// UGT1A10 /// UGT1A3 /// UGT1A4 /// UGT1A5 /// UGT1A6 /// UGT1A7 /// UGT1A8 /// UGT1A9 | UDP glucuronosyltransferase 1 family, polypeptide A10 ; UDP glucuronosyltransferase 1 family, polypeptide A8 ; UDP glucuronosyltransferase 1 family, polypeptide A7 ; UDP glucuronosyltransferase 1 family, polypeptide A6 ; UDP glucuronosyltransferase 1 family, polypeptide A5 ; UDP glucuronosyltransferase 1 family, polypeptide A9 ; UDP glucuronosyltransferase 1 family, polypeptide A4 ; UDP glucuronosyltransferase 1 family, polypeptide A1 ; UDP glucuronosyltransferase 1 family, polypeptide A3 | | -1.55 | 7.52x10^-03^ | 99 |
| 204532_x_at | UGT1A1 /// UGT1A10 /// UGT1A4 /// UGT1A6 /// UGT1A8 /// UGT1A9 | UDP glucuronosyltransferase 1 family, polypeptide A10 ; UDP glucuronosyltransferase 1 family, polypeptide A8 ; UDP glucuronosyltransferase 1 family, polypeptide A7 ; UDP glucuronosyltransferase 1 family, polypeptide A6 ; UDP glucuronosyltransferase 1 family, polypeptide A5 ; UDP glucuronosyltransferase 1 family, polypeptide A9 ; UDP glucuronosyltransferase 1 family, polypeptide A4 ; UDP glucuronosyltransferase 1 family, polypeptide A1 ; UDP glucuronosyltransferase 1 family, polypeptide A3 | | -1.56 | 3.04x10^-03^ | 100 |
| 207126_x_at | UGT1A1 /// UGT1A10 /// UGT1A4 /// UGT1A6 /// UGT1A8 /// UGT1A9 | UDP glucuronosyltransferase 1 family, polypeptide A10 ; UDP glucuronosyltransferase 1 family, polypeptide A8 ; UDP glucuronosyltransferase 1 family, polypeptide A7 ; UDP glucuronosyltransferase 1 family, polypeptide A6 ; UDP glucuronosyltransferase 1 family, polypeptide A5 ; UDP glucuronosyltransferase 1 family, polypeptide A9 ; UDP glucuronosyltransferase 1 family, polypeptide A4 ; UDP glucuronosyltransferase 1 family, polypeptide A1 ; UDP glucuronosyltransferase 1 family, polypeptide A3 | | -1.58 | 3.10x10^-03^ | 100 |
| 213022_s_at | UTRN | utrophin (homologous to dystrophin) | | 1.60 | 1.53x10^-03^ | 98 |
| 213023_at | UTRN | utrophin (homologous to dystrophin) | | 1.55 | 3.59x10^-03^ | 98 |
| 218806_s_at | VAV3 | vav 3 oncogene | | 1.54 | 5.41x10^-04^ | 100 |
| 224221_s_at | VAV3 | vav 3 oncogene | | 1.77 | 2.12x10^-03^ | 95 |
| 232122_s_at | VEPH1 | ventricular zone expressed PH domain homolog 1 (zebrafish) | | 1.55 | 9.37x10^-03^ | 76 |
| 222799_at | WDR91 | HSPC049 protein | | 1.57 | 3.94x10^-04^ | 44 |
| 215711_s_at | WEE1 | WEE1 homolog (S. pombe) | | 1.64 | 5.98x10^-04^ | 79 |
| 1552396_at | WFDC6 | WAP four-disulfide core domain 6 | | 1.75 | 3.47x10^-04^ | 98 |
| 213425_at | WNT5A | wingless-type MMTV integration site family, member 5A ; wingless-type MMTV integration site family, member 5A | | 1.56 | 4.40x10^-03^ | 99 |
| 227910_at | XPNPEP3 | hypothetical protein LOC63929 | | 2.10 | 2.70x10^-03^ | 43 |
| 215150_at | YOD1 | YOD1 OTU deubiquinating enzyme 1 homolog ( yeast) | | 1.76 | 6.88x10^-03^ | 59 |
| 225665_at | ZAK | sterile alpha motif and leucine zipper containing kinase AZK | | 1.54 | 3.94x10^-05^ | 100 |
| 219312_s_at | ZBTB10 | zinc finger and BTB domain containing 10 | | 1.57 | 5.41x10^-04^ | 100 |
| 217486_s_at | ZDHHC17 | zinc finger, DHHC-type containing 17 | | 1.55 | 4.10x10^-03^ | 43 |
| 213286_at | ZFR | zinc finger RNA binding protein | | 1.57 | 9.66x10^-03^ | 80 |
| 214900_at | ZKSCAN1 | zinc finger with KRAB and SCAN domains 1 | | 1.52 | 5.54x10^-03^ | 28 |
| 1554159_a_at | ZMYND11 | zinc finger, MYND domain containing 11 | | 1.88 | 6.24x10^-03^ | 70 |
| 206579_at | ZNF192 | zinc finger protein 192 | | 1.56 | 7.49x10^-03^ | 63 |
| 244462_at | ZNF224 | Zinc finger protein 224 | | 1.53 | 4.01x10^-03^ | 95 |
| 238454_at | ZNF540 | zinc finger protein 540 | | 1.59 | 3.37x10^-03^ | 68 |
| 1553225_s_at | ZNF75D | zinc finger protein 75 (D8C6) | | 1.59 | 8.92x10^-03^ | 74 |
| 238907_at | ZNF780A | CDNA FLJ16334 fis, clone TESOP2006865, moderately similar to ZINC FINGER PROTEIN MFG-3 | | 1.58 | 1.13x10^-03^ | 90 |
| 239441_at | ZNF780A |  |  | 2.10 | 2.20x10^-03^ | 66 |
| 1554007_at | --- |  |  | 1.60 | 7.03x10^-04^ | 100 |
| 1556185_a_at | --- | CDNA clone IMAGE:5260162 | | 1.77 | 9.94x10^-04^ | 95 |
| 1558445_at | --- | CDNA clone IMAGE:5277883 | | 1.52 | 5.38x10^-03^ | 83 |
| 1560226_at | --- | Myosin, heavy polypeptide 15 | | 1.69 | 2.28x10^-03^ | 70 |
| 1561937_x_at | --- | Truncated IgM chain mRNA, 5' end | | 1.96 | 5.08x10^-03^ | 60 |
| 228734_at | --- | Ubiquitin-conjugating enzyme E2 variant 2 | | 1.56 | 2.65x10^-03^ | 79 |
| 228850_s_at | --- |  |  | 1.65 | 1.11x10^-03^ | 96 |
| 228963_at | --- | MRNA; cDNA DKFZp434L201 (from clone DKFZp434L201) | | 1.50 | 3.46x10^-04^ | 99 |
| 229654_at | --- | Transcribed locus | | 2.05 | 3.94x10^-05^ | 90 |
| 230130_at | --- | Transcribed locus | | 1.87 | 2.93x10^-03^ | 99 |
| 230951_at | --- | Erythrocyte membrane protein band 4.1 like 5 | | 1.52 | 2.26x10^-04^ | 100 |
| 232064_at | --- | CDNA FLJ35001 fis, clone OCBBF2011887 | | 1.55 | 1.64x10^-03^ | 78 |
| 237351_at | --- | Hypothetical protein LOC284825 | | -1.90 | 3.37x10^-03^ | 95 |
| 238537_at | --- | Carbonic anhydrase VIII | | 2.12 | 2.54x10^-03^ | 34 |
| 238745_at | --- | Chromosome 8 open reading frame 35 | | 1.54 | 7.77x10^-03^ | 76 |
| 238861_at | --- | MRNA; clone CD 43T7 | | 1.53 | 1.14x10^-03^ | 93 |
| 239136_at | --- |  |  | 1.50 | 6.50x10^-03^ | 41 |
| 239184_at | --- | Transcribed locus, moderately similar to NP_009083.1 zinc finger protein 195 [Homo sapiens] | | 1.57 | 5.14x10^-03^ | 73 |
| 239262_at | --- | CDNA FLJ26242 fis, clone DMC00770 | | 1.66 | 8.98x10^-03^ | 59 |
| 239465_at | --- | Ubiquinol-cytochrome c reductase core protein II | | 1.82 | 5.54x10^-03^ | 61 |
| 239822_at | --- | CDNA clone IMAGE:4816129 | | 1.99 | 8.61x10^-03^ | 20 |
| 240125_at | --- | Dystrobrevin, alpha | | -1.85 | 1.05x10^-03^ | 79 |
| 240222_at | --- | Transcribed locus, weakly similar to XP_371841.1 PREDICTED: similar to hypothetical protein (L1H 3 region) - human [Homo sapiens] | | 1.59 | 4.07x10^-03^ | 25 |
| 243421_at | --- | Tachykinin receptor 1 | | 2.82 | 4.69x10^-03^ | 28 |
| 243735_at | --- | Signal transducer and activator of transcription 3 interacting protein 1 | | 1.56 | 4.11x10^-03^ | 80 |
| 243958_at | --- | Transcribed locus | | 1.81 | 9.19x10^-03^ | 25 |

^1^ Data obtained using the Affymetrix HG-U133 Plus 2.0 microarray chip.

^2^  Fold-change represents ratio of average expression value in healthy smokers to average expression value in healthy nonsmokers. Positive fold-changes represent genes upregulated by smoking; negative fold-changes represent genes down-regulated by smoking.

^3^ p value obtained using Benjamini-Hochberg correction to limit the false positive rate.

^4^ P call represents the % of healthy nonsmoker and healthy smoker samples in which the Affymetrix detection call for that probe set was “P” or “Present,” meaning that the gene was expressed in that sample.

**References**

1. A. Spira, J. Beane, V. Shah, G. Liu, F. Schembri, X. Yang, J. Palma, J. S. Brody, Effects of cigarette smoke on the human airway epithelial cell transcriptome. *Proc. Natl. Acad. Sci U. S. A* 101, 10143-10148 (2004).

2. L. I. Zhang, J. Lee, H. Tang, Y. H. Fan, L. Xiao, H. Ren, J. Kurie, R. C. Morice, W. K. Hong, H. Mao, Impact of smoking cessation on global gene expression in the bronchial epithelium of chronic smokers. *Cancer Prev Res* 1, 112-118 (2008).
