# Supplementary material for: Biologic Phenotyping of the Human Small Airway Epithelial Response to Cigarette Smoking
Source: PLoS One. 2011 Jul 28;6(7):e22798. doi: 10.1371/journal.pone.0022798 (PMC3145669; doi:10.1371/journal.pone.0022798)
Supplement: Text S1 — (DOC) [file pone.0022798.s013.doc]

**Supporting Methods**

**Inclusion and Exclusion Criteria**

**Healthy nonsmokers**

**Inclusion criteria**

1. Must be capable of providing informed consent
2. Males and females, age 18 or older
3. Never-smokers by history, with current smoking status validated by the absence of nicotine metabolites in urine [1]
4. Good overall health without history of chronic lung disease, including asthma, and without recurrent or recent (within 3 months) acute pulmonary disease
5. Normal physical examination
6. Normal routine laboratory evaluation, including general hematologic studies, general serologic/immunologic studies, general biochemical analyses, and urine analysis
7. Negative HIV serology
8. Normal chest X-ray (PA and lateral)
9. Normal electrocardiogram (sinus bradycardia, premature atrial contractions are permissible)
10. Females - not pregnant
11. No history of allergies to medications to be used in the bronchoscopy procedure
12. Not taking any medications relevant to lung disease or having an effect on the airway epithelium
13. Willingness to participate in the study

**Exclusion criteria**

 Unable to meet the inclusion criteria

 Pregnancy

 Current active infection or acute illness of any kind

 Current alcohol or drug abuse

 Evidence of malignancy within the past 5 years

**Healthy smokers**

**Inclusion criteria**

1. Must be capable of providing informed consent
2. Males and females, age 18 or older
3. Current daily smokers with any number of pack-yr, validated by any of the following: urine nicotine >30 ng/ml or urine cotinine >50 ng/ml [1]
4. Good overall health without history of chronic lung disease, including asthma, and without recurrent or recent (within 3 months) acute pulmonary disease
5. Normal physical examination
6. Normal routine laboratory evaluation, including general hematologic studies, general serologic/immunologic studies, general biochemical analyses, and urine analysis
7. Negative HIV serology
8. Normal chest X-ray (PA and lateral)
9. Normal electrocardiogram (sinus bradycardia, premature atrial contractions are permissible)
10. Females - not pregnant
11. No history of allergies to medications to be used in the bronchoscopy procedure
12. Not taking any medications relevant to lung disease or having an effect on the airway epithelium
13. Willingness to participate in the study

**Exclusion criteria**

 Unable to meet the inclusion criteria

 Pregnancy

 Current active infection or acute illness of any kind

 Current alcohol or drug abuse

 Evidence of malignancy within the past 5 years

**Smokers with COPD**

**Inclusion criteria**

1. Must be capable of providing informed consent
2. Males and females, age 18 or older
3. Current daily smokers with any number of pack-yr, validated by any of the following: urine nicotine >30 ng/ml or urine cotinine >50 ng/ml [1]
4. Meeting GOLD stages I-III criteria for chronic obstructive lung disease (COPD) based on post-bronchodilator spirometry
5. Taking any or no pulmonary-related medication, including beta-agonists, anticholinergics, or inhaled corticosteroids
6. Normal routine laboratory evaluation, including general hematologic studies, general serologic/immunologic studies, general biochemical analyses, and urine analysis

 Females - not pregnant

 Negative HIV serology

1. Normal electrocardiogram (sinus bradycardia, premature atrial contractions are permissible)
2. All individuals have chest X-ray (PA and lateral) and chest CT
3. No history of allergies to medications to be used in the bronchoscopy procedure
4. Willingness to participate in the study

**Exclusion criteria**

 Unable to meet the inclusion criteria

 Individuals in whom participation in the study would compromise the normal care and expected progression of their disease

 Current active infection or acute illness of any kind

 Current alcohol or drug abuse

 Evidence of malignancy within the past 5 years

**Sampling Airway Epithelium**

Flexible bronchoscopy was used to collect 10th to 12th generation small airway epithelial cells by brushing the epithelium as previously described [2]. Cells were detached from the brush by flicking into 5 ml of ice-cold LHC8 medium (GIBCO, Grand Island, NY). An aliquot of 0.5 ml was used for differential cell count (typically 2x10^4^ cells per slide). The remainder (4.5 ml) was processed immediately for RNA extraction. The number of cells recovered by brushing was determined by counting on a hemocytometer. To quantify the percentage of epithelial and inflammatory cells and the proportions of basal, ciliated, secretory and undifferentiated cells recovered, cells were prepared by centrifugation (Cytospin 11, Shandon Instruments, Pittsburgh, PA) and stained with Diff-Quik (Baxter Healthcare, Miami, FL), and differential cell counts were performed. Samples were confirmed to be bona fide small airway samples by expression of genes encoding surfactant proteins and Clara cell secretory protein [2].

**RNA and Microarray Processing**

The HG-U133 Plus 2.0 array (Affymetrix, Santa Clara, CA), which includes probes for over 47,000 transcripts genome-wide, was used to evaluate gene expression. Total RNA was extracted using a modified version of the TRIzol method (InVitrogen, Carlsbad, CA), in which RNA is purified directly from the aqueous phase (RNeasy MinElute RNA purification kit, Qiagen, Valencia, CA). RNA samples were stored in RNA Secure (Ambion, Austin, TX) at -80C. RNA integrity was determined by running an aliquot of each RNA sample on an Agilent Bioanalyzer (Agilent Technologies, Palo Alto, CA). The concentration was determined using a NanoDrop ND-1000 spectrophotometer (NanoDrop Technologies, Wilmington, DE). Double stranded cDNA was synthesized from 1 to 2 μg total RNA using the GeneChip One-Cycle cDNA Synthesis Kit, followed by cleanup with GeneChip Sample Cleanup Module, in vitro transcription (IVT) reaction using the GeneChip IVT Labeling Kit, and cleanup and quantification of the biotin-labeled cDNA yield by spectrophotometry. All kits were from Affymetrix (Santa Clara, CA). All HG-U133 Plus 2.0 microarrays were processed according to Affymetrix protocols, hardware and software, including being processed by the Affymetrix fluidics station 450 and hybridization oven 640 and scanned with an Affymetrix Gene Array Scanner 3000 7G. Overall microarray quality was verified by the following criteria [3-6]: (1) RNA Integrity Number (RIN) >7.0; (2) 3'/5' ratio for GAPDH <3; and (3) scaling factor <10.0.

**Singular Value Decomposition and Prediction Analysis of Microarrays**

Singular value decomposition was performed as described by Alter et al [7] to identify genes that were differentially expressed between healthy smokers and nonsmokers. The expression values of all probes with a P call of “Present” in 20% of samples were assembled into a matrix of 31,067 probes by 105 smoker and nonsmoker samples. Each column and row was normalized to unit variance by dividing each row or column by the L-2 norm (the square root of the sum of the squares) of the row or column, respectively. The eigengene that corresponded to the smoking response was identified by a t test and the top loading genes were identified by the corresponding eigenarray.

Prediction analysis of microarrays (PAM) was performed using the R package supplied by Tibshirani et al [8] (http://www-stat.stanford.edu/~tibs/PAM/) using nearest shrunken centroid classification to identify genes differentially expressed between healthy smokers and nonsmokers using the genome-wide expression data for all probes with a P call of “Present” in 20% of samples.

**TaqMan Real-Time RT-PCR Confirmation of Microarray Expression Changes**

TaqMan real-time RT-PCR was performed on RNA samples from the small airways of 14 healthy nonsmokers and 14 healthy smokers (except as noted) to confirm the expression levels of examples of significantly changed genes. For each individual, cDNA was synthesized from 2 μg RNA in a 100 l reaction volume, using the TaqMan Reverse Transcriptase Reaction Kit (Applied Biosystems), with random hexamers as primers. Dilutions of 1:10 and 1:100 were made from each sample and triplicate wells were run for each dilution. TaqMan PCR reactions were carried out using pre-made assays from Applied Biosystems using 2 μl of cDNA in each 25 l reaction volume. The endogenous control was 18S ribosomal RNA and relative expression levels were determined using the Ct method (Applied Biosystems). The average relative expression level for nonsmokers (i.e., average Ct) was used as the calibrator. The rRNA probe was labeled with VIC and the probe for each gene of interest was labeled with FAM. The PCR reactions were run in an Applied Biosystems Sequence Detection System 7500.

**Analyses Using the I_SAE_**

The I_SAE_ was calculated for each individual. The median values for healthy nonsmokers, healthy smokers and the subgroups of low and high responder smokers were determined. Significant differences in the medians were evaluated using a Mann-Whitney U test. The spread of the index values within each group was described by the variance and by the interquartile range. F tests were used to compare the variances for significant differences.

A variety of tests were carried out to ensure that the I_SAE_ was not confounded by factors other than the inherent responses of the small airway epithelium. A Kruskal-Wallis test was used to assess for possible effects of gender, ancestry, age, FEV1, FEV1/FVC, FEF25-75, and date of the hybridization to the microarray. The effect of pack-yr and smoking duration in years on the healthy smoker group was tested using a linear regression analysis. Clinical parameters including demographics, smoking related parameters, lung function parameters, and procedure-related parameters were compared for high *vs* low responder smokers. Significance was assessed using a t test for continuous variables and chi-square test for categorial variables, and the significant p value of 0.05 was adjusted for multiple comparisons.

To evaluate the robustness and sampling properties of the I_SAE_, we used a K-fold cross-validation approach. Since the I_SAE_ for each individual depends on the normal range of gene expression values derived from the nonsmokers (and not the smokers), the K-fold analysis focused on the nonsmokers. For the analysis, 20 of the nonsmokers were randomly selected, means and standard deviations for the 375 smoking-responsive genes were recalculated, and I_SAE_ was recalculated for the remaining nonsmokers and the healthy smokers. The distribution of I_SAE_ values was then assessed as before. This approach was repeated for 500 random samples.

**Validation of Classification of High and Low Responder Smokers**

Three independent methods were used to verify the significance of the signal separating high from low responder smokers. First, a singular value decomposition was performed on the 647 smoking-responsive probe sets that were identified in the initial analysis, on the 58 healthy smokers as a regular principal components analysis, i.e., by mean centering and normalizing each probe set and array to unit variance and by evaluating the ability of the 647 genes to discriminate between high and low responder smokers in this method. Second, prediction analysis of microarrays (PAM) was performed using the R package as described above to classify the 58 healthy smokers into high and low responders using the 647 probe sets. Third, a support vector machine was constructed using LIBSVM [9] (“Library for Support Vector Machines”), an integrated software for support vector classification. The support vector machine was constructed using a linear kernal and the ability to separate high responders from low responders was verified using three-way cross-validation.

**References**

1. Moyer TP, Charlson JR, Enger RJ, Dale LC, Ebbert JO, Schroeder DR, Hurt RD. Simultaneous analysis of nicotine, nicotine metabolites, and tobacco alkaloids in serum or urine by tandem mass spectrometry, with clinically relevant metabolic profiles. *Clin Chem* 2002; 48: 1460-1471

2. Harvey BG, Heguy A, Leopold PL, Carolan BJ, Ferris B, Crystal RG. Modification of gene expression of the small airway epithelium in response to cigarette smoking. *J Mol Med* 2007; 85: 39-53

3. Jung M, Ramankulov A, Roigas J, Johannsen M, Ringsdorf M, Kristiansen G, Jung K. In search of suitable reference genes for gene expression studies of human renal cell carcinoma by real-time PCR. *BMC Mol Biol* 2007; 8: 47

4. Stan AD, Ghose S, Gao XM, Roberts RC, Lewis-Amezcua K, Hatanpaa KJ, Tamminga CA. Human postmortem tissue: what quality markers matter? *Brain Res* 2006; 1123: 1-11

5. Thompson KL, Pine PS, Rosenzweig BA, Turpaz Y, Retief J. Characterization of the effect of sample quality on high density oligonucleotide microarray data using progressively degraded rat liver RNA. *BMC Biotechnol* 2007; 7: 57

6. Tumor Analysis Best Practices Working Group. Expression profiling--best practices for data generation and interpretation in clinical trials. *Nat Rev Genet* 2004; 5: 229-237

7. Alter O, Golub GH. Singular value decomposition of genome-scale mRNA lengths distribution reveals asymmetry in RNA gel electrophoresis band broadening. *Proc Natl Acad Sci U S A* 2006; 103: 11828-11833

8. Tibshirani R, Hastie T, Narasimhan B, Chu G. Diagnosis of multiple cancer types by shrunken centroids of gene expression. *Proc Natl Acad Sci U S A* 2002; 99: 6567-6572

9. Chang CC, Lin CJ. A library for support vector machines. *www csie ntu edu tw/~cjlin/libsvm* 2001
